# Compound heterozygous variants in *OTULIN* are associated with fulminant atypical late‐onset ORAS

**DOI:** 10.15252/emmm.202114901

**Published:** 2022-02-16

**Authors:** Julia Zinngrebe, Barbara Moepps, Thomas Monecke, Peter Gierschik, Ferdinand Schlichtig, Thomas F E Barth, Gudrun Strauß, Elena Boldrin, Carsten Posovszky, Ansgar Schulz, Ortraud Beringer, Eva Rieser, Eva‐Maria Jacobsen, Myriam Ricarda Lorenz, Klaus Schwarz, Ulrich Pannicke, Henning Walczak, Dierk Niessing, Catharina Schuetz, Pamela Fischer‐Posovszky, Klaus‐Michael Debatin

**Affiliations:** ^1^ Department of Pediatrics and Adolescent Medicine Ulm University Medical Center Ulm Germany; ^2^ Institute of Pharmacology and Toxicology Ulm University Ulm Germany; ^3^ Institute of Pharmaceutical Biotechnology Ulm University Ulm Germany; ^4^ Department of Pathology University Medical Center Ulm Ulm Germany; ^5^ Institute of Biochemistry I & CECAD Research Center University of Cologne Cologne Germany; ^6^ Institute for Transfusion Medicine Ulm University Ulm Germany; ^7^ Institute for Clinical Transfusion Medicine and Immunogenetics Ulm German Red Cross Blood Service Baden‐Wuerttemberg – Hessen Ulm Germany; ^8^ UCL Cancer Institute London UK; ^9^ Pediatric Immunology Technical University Dresden Dresden Germany

**Keywords:** autoinflammation, linear ubiquitin, LUBAC, ORAS, OTULIN, Genetics, Gene Therapy & Genetic Disease

## Abstract

Autoinflammatory diseases are a heterogenous group of disorders defined by fever and systemic inflammation suggesting involvement of genes regulating innate immune responses. Patients with homozygous loss‐of‐function variants in the OTU‐deubiquitinase *OTULIN* suffer from neonatal‐onset OTULIN‐related autoinflammatory syndrome (ORAS) characterized by fever, panniculitis, diarrhea, and arthritis. Here, we describe an atypical form of ORAS with distinct clinical manifestation of the disease caused by two new compound heterozygous variants (c.258G>A (p.M86I)/c.500G>C (p.W167S)) in the *OTULIN* gene in a 7‐year‐old affected by a life‐threatening autoinflammatory episode with sterile abscess formation. On the molecular level, we find binding of OTULIN to linear ubiquitin to be compromised by both variants; however, protein stability and catalytic activity is most affected by OTULIN variant p.W167S. These molecular changes together lead to increased levels of linear ubiquitin linkages in patient‐derived cells triggering the disease. Our data indicate that the spectrum of ORAS patients is more diverse than previously thought and, thus, supposedly asymptomatic individuals might also be affected. Based on our results, we propose to subdivide the ORAS into classical and atypical entities.

## Graphical abstract

The OTULIN‐Related Autoinflammatory Syndrome (ORAS) is known to be caused by homozygous variants in the OTULIN gene. This study discovers that two new compound‐heterozygous variants in OTULIN are associated with an atypical, but potentially fatal, late‐onset form of ORAS.

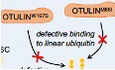

**The paper explained**

**Problem**

Ubiquitination and its reversal process, deubiquitination, are crucial for the regulation of immune signaling pathways. Thus, it is not surprising that defective (de)ubiquitination has been identified as the underlying cause of a new category of autoinflammatory disorders. One of these disorders, caused by homozygous variants in the deubiquitinase OTULIN, is known as OTULIN‐Related Autoinflammatory Syndrome (ORAS) or Otulipenia. The ORAS is characterized by neonatal‐onset fever, panniculitis, diarrhea, and arthritis. How compound heterozygous mutations in OTULIN manifest clinically is currently unknown.

**Results**

In this study, we identify two new compound heterozygous variants in OTULIN in a patient without overt signs of autoinflammation until the age of seven who then encountered a severe autoinflammatory episode with multiorgan abscess formation. In‐depth biochemical analysis revealed how both OTULIN variants contribute to diminished OTULIN protein expression and defective hydrolysis of linear ubiquitin linkages observed in patient‐derived cells: whereas binding of OTULIN to linear ubiquitin was compromised by both variants, protein stability and catalytic activity was most affected by the OTULIN variant p.W167S. Although this patient differs clinically from published patients with ORAS, the pathogenesis of the disease seems to be similar and also triggered by perturbed TNF signaling.

**Impact**

The number of patients with ORAS, first described in 2016, is still very limited. Thus, we can learn a lot from each additional ORAS patient. This study indicates that the clinical spectrum of ORAS patients is more diverse than previously thought and multiorgan sterile abscess formation and potential clinical inapparency should be added to the list of ORAS symptoms. Due to the differences in clinical presentation between this patient with compound heterozygous OTULIN variants and previously identified patients with homozygous OTULIN variants, we suggest to divide the ORAS into classical and atypical entities.

## Introduction

Autoinflammation describes a group of inherited, mostly monogenic disorders with recurrent fever and systemic inflammation in the absence of identifiable infectious agents (Manthiram *et al*, 2017). The post‐translational modification of proteins by ubiquitin plays an essential role in the regulation of immune signaling pathways, in particular in the innate immune response (Zinngrebe *et al*, 2014). Variants in genes involved in ubiquitination and its reversal process, deubiquitination, have been identified as underlying cause of a new category of autoinflammatory diseases (Aksentijevich & Zhou, 2017; Beck & Aksentijevich, 2019).

Ubiquitination links ubiquitin molecules to substrate proteins, or to one another via the C‐terminal carboxyl group of the donor ubiquitin and one of the seven internal lysine (K) residues or the N‐terminal methionine (M) 1 of the acceptor ubiquitin. This results in a total of eight different inter‐ubiquitin linkage types (Spit *et al*, 2019). M1‐linkages, also known as linear ubiquitin linkages, are assembled in a head‐to‐tail fashion (Kirisako *et al*, 2006) by a tripartite protein complex called linear ubiquitin chain assembly complex (LUBAC) consisting of Shank‐Associated RH Domain‐Interacting Protein (SHARPIN), Heme‐Oxidized IRP2 Ubiquitin Ligase 1 (HOIL‐1), and HOIL‐1‐Interacting Protein (HOIP) (Gerlach *et al*, 2011; Ikeda *et al*, 2011; Tokunaga *et al*, 2011).

Ubiquitin linkages are disassembled by so‐called deubiquitinating enzymes (DUBs). In 2013, the OTU‐deubiquitinase with linear linkage specificity (OTULIN; also known as FAM105B or Gumby) was identified to specifically bind to and hydrolyze linear ubiquitin linkages assembled by LUBAC (Keusekotten *et al*, 2013; Rivkin *et al*, 2013).

Dysregulation of linear ubiquitin linkages is associated with numerous human diseases, including immune disorders, cancer, and neurodegeneration (Jahan *et al*, 2021). Variants affecting OTULIN’s catalytic activity, resulting in increased linear ubiquitin linkages, cause embryonic lethality in mice (Rivkin *et al*, 2013; Heger *et al*, 2018). Moreover, two independent groups identified a surplus of linear ubiquitin linkages in humans due to homozygous variants in *OTULIN* to result in an autoinflammatory disease: OTULIN‐Related Autoinflammatory Syndrome (ORAS) or Otulipenia (Damgaard *et al*, 2016; Zhou *et al*, 2016). Eight patients with ORAS carrying homozygous missense or premature stop variants in the *OTULIN* gene have been identified to date (Damgaard *et al*, 2016, 2019, 2020; Zhou *et al*, 2016; Nabavi *et al*, 2019) (Table EV1). All reported patients were born prematurely, showed first signs of disease within weeks after birth, and suffered from fever, nodular panniculitis, failure to thrive, diarrhea, and arthritis accompanied by increased levels of leukocytes, neutrophils, and C‐reactive protein (CrP) (Damgaard *et al*, 2016, 2019, 2020; Zhou *et al*, 2016; Nabavi *et al*, 2019). One ORAS patient carrying a homozygous missense mutation in OTULIN (Damgaard *et al*, 2016) additionally suffered from steatosis and hepatocyte degeneration with abnormal liver values (Damgaard *et al*, 2020) suggesting that functioning OTULIN is also essential for liver health. This is further supported by the fact that mice with liver‐specific deletion of OTULIN show a similar disease phenotype with liver inflammation and apoptosis ultimately leading to formation of hepatocellular carcinoma (Damgaard *et al*, 2020; Verboom *et al*, 2020).

In the present study, we identified a 7‐year‐old boy with compound heterozygosity in *OTULIN* carrying two different heterozygous variants with one variant on each allele of the *OTULIN* gene. He suffered from an atypical form of ORAS with late‐onset manifesting as a fulminant autoinflammatory episode with sterile abscess formation in different organs including skin, lung, and spleen. By performing structural and biochemical analyses, *OTULIN* gene deletion and reconstitution experiments with different *OTULIN* variants in a heterologous cell system and by assessing response of patient‐derived fibroblasts and B cells to immune stimuli, we provide characterization of the combined impact of the two different *OTULIN* variants on OTULIN’s function on both, molecular and functional levels.

## Results

### Sterile abscess formation in a patient with compound‐heterozygous missense variants in the *OTULIN* gene

**Figure 1:**
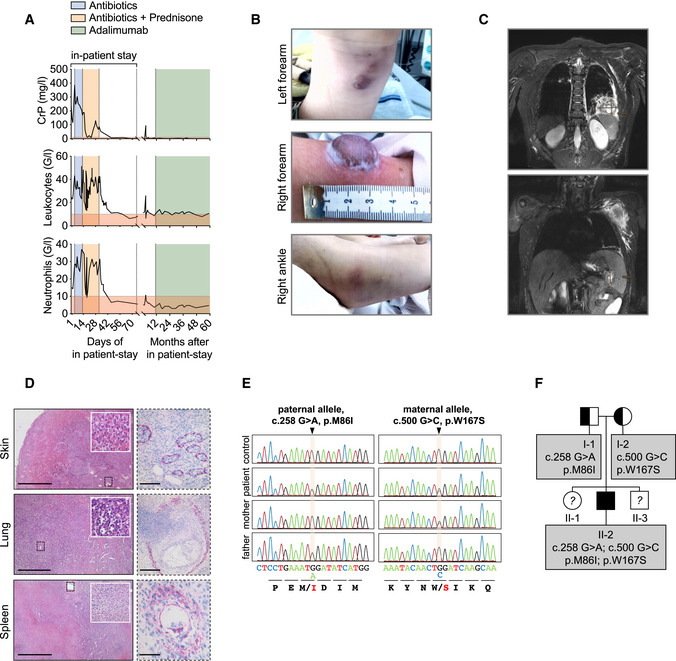
Sterile abscess formation in a patient with compound‐heterozygous missense variants in the *OTULIN* gene Blood parameters of patient are depicted.Patient’s skin alterations are depicted.T2‐weighted MR images in coronal plane show abscess formation in the left lower pulmonary lobe (upper panel) and in spleen and left axilla (lower panel).Histological sections of patient biopsies stained with hematoxylin and eosin (left panel) or with an actin antibody (right panel). Scale bars: skin: left 1,000 µm, right 100 µm; lung: left 500 µm, right 250 µm; spleen: left 1,000 µm, right 75 µm.Whole Exome Sequencing (WES) and targeted Sanger sequencing identified compound heterozygous variants in *OTULIN* at position c.258 G > A, p.M86I on the paternal allele and at position c.500 G > C, p.W167S on the maternal allele.The pedigree is depicted. The patient (II‐2) is the second child of non‐consanguineous parents.

A 7‐year‐old male patient of Greek origin was admitted with abdominal pain and subfebrile temperatures. The boy’s psychomotor development was age‐appropriate, and he was obese (body weight: 36.7 kg, height: 1.29 m, body mass index (BMI): 22.1 kg/m^2^ (97^th^ age‐specific BMI percentile)). He had previously suffered from a pneumonia at the age of 6 months, an appendicitis at the age of 6 years, and a gluteal abscess which had been difficult to treat. Initially, he presented with leukocytosis (25.5 G/l; normal range: 4.5–13.5 G/l), neutrophilia (14.93 G/l; normal range: 1.8–8 G/l), and highly elevated levels of CrP (241 mg/l; normal range: < 10 mg/l) (Fig 1A). Shortly after admission, he developed spiking fevers with continuously increasing inflammatory parameters (Fig 1A). Treatment with broad‐spectrum antibiotics did not influence the course of systemic inflammatory response syndrome. During further course, the patient developed inflammatory lesions on the left and right wrists and the right ankle (Fig 1B). Total body magnetic resonance imaging (MRI) further revealed abscess formation in the left lower pulmonary lobe, in the left axilla, and in the spleen (Fig 1C). The patient was transferred to intensive care unit (ICU) and underwent the following surgical procedures: debridement of lesions on the wrists, axillary dissection, partial resection of lung and pancreas, and splenectomy. Pus was drained from multiple sites of inflammation; however, biopsies and smears remained sterile (Appendix Table S1). All blood cultures, stool cultures, throat and anal swabs, and tracheal fluids remained sterile (Appendix Table S2). Histopathological analysis of the skin (Fig 1D) revealed massive inflammatory infiltrates of the corium, predominantly consisting of granulocytes, monocytes, and macrophages. In the lung, we found partially necrotizing infiltrates with neutrophils, and also, the spleen showed signs of inflammation and necrosis (Fig 1D). Eosinophils or giant cells were not detected. A monoclonal antibody directed against actin to visualize small blood vessels showed disruption of vessel walls by inflammatory cells in all three organs (Fig 1D). Although the patient’s urine was positive for *Pneumococcal* antigen (Appendix Table S2), gram‐positive bacteria were not detectable in biopsies of lung, spleen, and skin (Appendix Table S3).

**Figure EV1:**
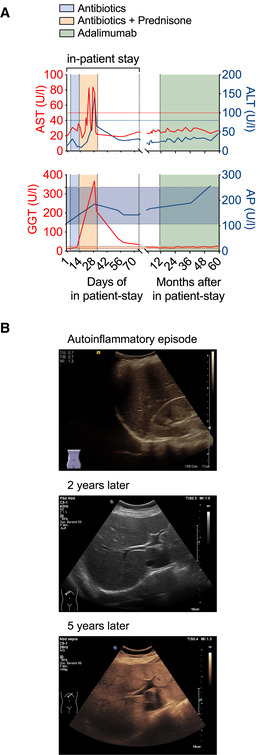
Abnormal liver function test and development of steatosis hepatis grade II The course of the patient’s liver enzymes AST (aspartate transaminase) and ALT (alanine transaminase) (upper panel) or GGT (γ‐glutamyltransferase) and AP (alkaline phosphatase) (lower panel) are depicted along the time axis with the upper limit of normal indicated by red and blue lines for AST and ALT and the normal range in shades of red and blue for GGT and AP, respectively.Liver ultrasound B‐mode images of the patient during the initial phase of autoinflammation and about 2 and 5 years thereafter display diffuse increase of liver echogenicity and finally slightly impaired appearance of portal vein wall and diaphragm indicative of steatosis hepatis grade II.

Since no infectious agent was identified and broad‐spectrum antibiotics had not improved the patient’s condition, an autoinflammatory syndrome was suspected, and additional treatment with corticosteroids was started on day 14 of in‐patient stay (Fig 1A). This resulted in a decline of body temperature and CrP levels and in marked improvement of the patient’s condition. Liver enzymes such as aspartate transaminase (AST), alanine transaminase (ALT), or gamma‐glutamyltransferase (GGT) were elevated at this time and returned to normal in the further course of the disease (Fig EV1A). Alkaline phosphatase (AP) (Fig EV1A), but also total bilirubin, prothrombin, and activated partial thromboplastin were within normal range at all times, and an ultrasound examination of the liver during the autoinflammatory episode showed no abnormalities (Fig EV1B, upper panel). During recovery, the patient developed a pneumothorax and suffered from several bleeding duodenal ulcer requiring application of endoclips. No signs of pathology apart from uncharacteristic inflammation in the gastric antrum (Appendix Table S3) were found in biopsies. Two months following admission, the patient was discharged in good condition.

To identify the underlying cause of the severe and life‐threatening autoinflammatory episode in this patient, we performed whole exome sequencing (WES) and targeted Sanger sequencing revealing compound heterozygous missense variants in exon 3 (*c.258G>A;* p.M86I) and exon 5 (*c.500G>C;* p.W167S) (Fig 1E) of the *OTULIN* gene, respectively. WES revealed no other homozygous, compound heterozygous or pathogenic variants likely to explain the observed disease phenotype (Appendix Tables S4–S7; for filtering strategy see Appendix Fig S1). The patient inherited the p.M86I variant from his father, whereas his mother is a heterozygous carrier of the p.W167S mutation. The patient is the second child born to non‐consanguineous parents (patient II‐2; Fig 1F). Both, his parents and siblings are clinically well.

### The compound‐heterozygous missense variants p.M86I and p.W167S affect OTULIN protein expression and function

**Figure EV2:**
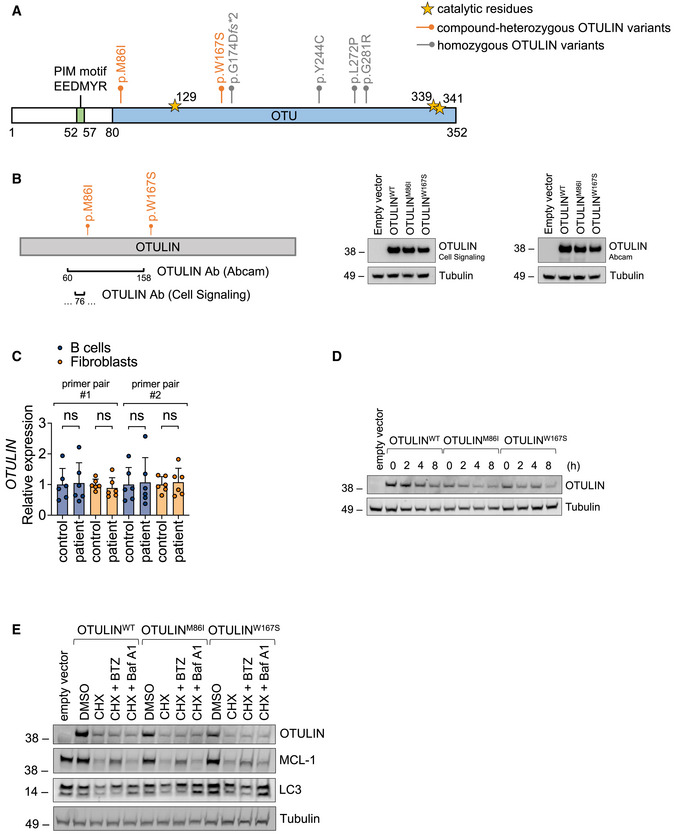
Diminished OTULIN protein expression in patient‐derived cells is not due to reduced mRNA expression or increased degradation *via* the proteasome or the lysosome OTULIN protein is depicted.OTULIN antibodies by Abcam (antigen corresponding to AA 60–158) or by Cell Signaling (recombinant fragment surrounds S76 without spanning M86) were tested in parallel on A549 *OTULIN* KO cells transfected with different OTULIN constructs as indicated. One representative of two independent experiments is shown.Relative mRNA expression of *OTULIN* in fibroblasts and B cells with two different primer pairs is depicted. Data are presented as mean ± SD of six independent experiments; dots represent individual experiments performed in three technical replicates; ns, non‐significant, unpaired *t*‐test.A549 *OTULIN* KO cells were transfected with the different OTULIN constructs as indicated. The following day, cells were treated with 50 µg/ml cycloheximide (CHX) for the indicated times, harvested and analyzed by Western blot for OTULIN protein expression. Tubulin served as loading control. One representative of three independent experiments is shown.A549 *OTULIN* KO cells were transfected with the different OTULIN constructs as indicated. The following day, cells were treated with 50 µg/ml CHX alone or in combination with 1 µM Bortezomib (BTZ) or 1 µM Bafilomycin A1 (Baf A1) for 8 h or left untreated (DMSO). Expression of the proteins indicated was analyzed by Western blot. One representative of three independent experiments is shown.

**Figure 2:**
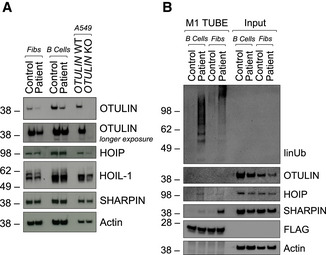
The compound‐heterozygous missense variants p.M86I and p.W167S affect OTULIN protein expression and function Expression of indicated proteins was determined by Western blot in patient‐derived and control fibroblasts and B cells and A549 *OTULIN* WT and KO cells.FLAG‐tagged tandem ubiquitin binding entity (TUBE) assay was performed to pull down linear ubiquitin linkages in patient‐derived and control fibroblasts and B cells. One representative (A, B) out of three independent experiments is shown. Source data are available online for this figure.

Missense or premature stop variants in *OTULIN* cause ORAS (Damgaard *et al*, 2016, 2019; Zhou *et al*, 2016; Nabavi *et al*, 2019). All published disease‐causing variants in *OTULIN* are homozygous (Damgaard *et al*, 2016, 2019; Zhou *et al*, 2016; Nabavi *et al*, 2019) (Fig EV2A). As the patient’s phenotype differed considerably from published patients with ORAS (Table EV1), we assessed whether the identified compound heterozygosity in the *OTULIN* gene affected OTULIN protein expression and/or function. OTULIN protein expression was diminished in patient‐derived fibroblasts and B cells compared to control (Fig 2A). As loss of OTULIN was shown to result in downregulation of LUBAC components in B cells (Damgaard *et al*, 2016; Heger *et al*, 2018), we also determined protein expression of SHARPIN, HOIL‐1, and HOIP. Expression of the different LUBAC components remained stable in patient‐derived cells (Fig 2A). To assess whether the diminished OTULIN expression was due to compromised antibody binding to OTULIN variant p.M86I, we applied a second commercially available antibody and confirmed equal detection of wildtype (OTULIN^WT^) and variant (OTULIN^M86I^ or OTULIN^W167S^) OTULIN protein overexpressed in A549 *OTULIN* KO cells (Fig EV2B). *OTULIN* mRNA expression was unchanged in patient‐derived fibroblasts and B cells (Fig EV2C). The homozygous OTULIN mutation p.G281R was reported to result in diminished protein expression due to increased OTULIN degradation via the proteasome (Damgaard *et al*, 2019). The half‐life of wildtype and variant OTULIN was similar (Fig EV2D). However, neither co‐incubation with the proteasome inhibitor Bortezomib (BTZ) nor with Bafilomycin A1 (Baf A1), an inhibitor of lysosomal protein degradation, was capable of stabilizing recombinant wildtype and variant OTULIN protein upon incubation with CHX (Fig EV2E). MCL‐1, a protein with high turnover (Wu *et al*, 2020), and LC3‐II, a marker of autophagosomes (Yoshii & Mizushima, 2017), served as positive controls for BTZ and Baf A1, respectively (Fig EV2E).

One function of OTULIN is the cleavage of linear ubiquitin linkages (Verboom *et al*, 2021; Weinelt & van Wijk, 2021). Numerous studies demonstrated that OTULIN downregulation or knockout as well as the expression of certain OTULIN variants lead to accumulation of linear ubiquitin linkages in cells (Fiil *et al*, 2013; Keusekotten *et al*, 2013; Rivkin *et al*, 2013; Elliott *et al*, 2014; Draber *et al*, 2015; Damgaard *et al*, 2016; Hrdinka *et al*, 2016; Zhou *et al*, 2016; van Wijk *et al*, 2017; Heger *et al*, 2018). Thus, we next assessed the level of linear ubiquitin linkages in patient‐derived and control cells. To enrich for linear ubiquitin, a FLAG‐tagged tandem ubiquitin binding entity (TUBE) reagent was used to pull down linear ubiquitin linkages and associated proteins (Fig 2B). Importantly, linear ubiquitin chains and associated LUBAC components SHARPIN and HOIP were increased in both, patient‐derived fibroblasts and B cells (Fig 2B).

Taken together, these results show that the compound heterozygous variants identified by WES in the *OTULIN* gene compromised OTULIN’s expression and DUB activity in patient‐derived cells.

### Pathogenic potential of OTULIN variants p.M86I and p.W167S

**Figure 3:**
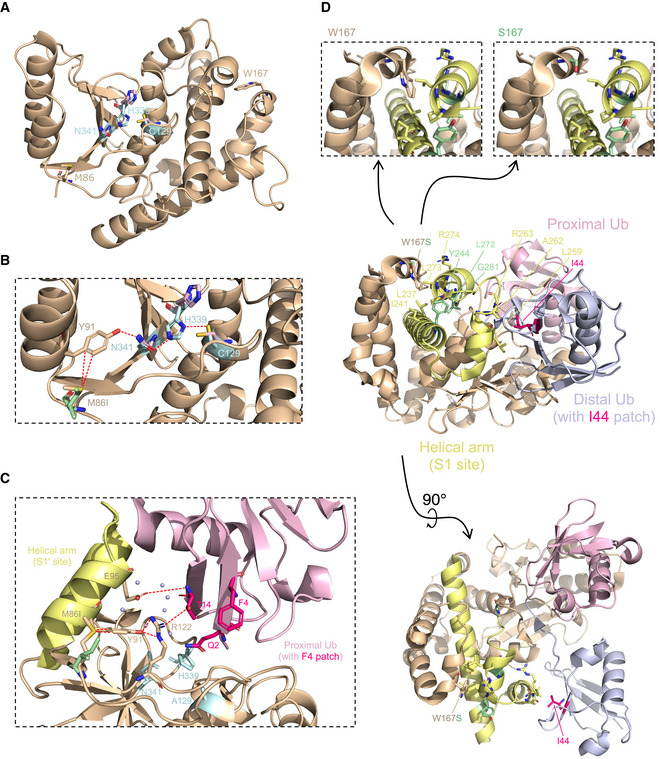
Pathogenic potential of OTULIN variants p.M86I and p.W167S The overall structure of OTULIN^WT^ (3ZNV) is depicted with its catalytic triad (C129, H339, N341; pale cyan) and positions of variants identified in this study (M86, W167).Close‐up of M86 (wheat) and M86I (pale green) with the interaction network around OTULIN’s catalytic center.Close‐up of OTULIN^C129A^ and its interaction with the proximal ubiquitin (Ub). M86, Y91, E95, and R122 of OTULIN and their interaction with each other and with the F4 patch of the proximal Ub are depicted. Interactions (dotted red lines) and water molecules (light blue) < 5 Å are shown. Residues of the catalytic triad (A129, H339, N341) in pale cyan.OTULIN^W167S^ in complex with di‐Ub. Contact sites of W167 (L237, I241, L273, and R274) in pale yellow. Positions of known homozygous OTULIN substitutions (Y244, L272, G281) in pale green. I44 patch of distal Ub in magenta and its contact sites L259, A262, and R263 in pale yellow. Close‐up of W167 and S167: loss of size and gain of polarity from W to S.

The potential pathogenicity of the two novel gene variants identified in this study was further analyzed using Ensembl Variant Effect Predictor (McLaren *et al*, 2016). Sorting Intolerant From Tolerant (SIFT) (Sim *et al*, 2012) and PolyPhen‐2 (Adzhubei *et al*, 2010) algorithms predicted the maternal, rather than the paternal, variant to affect OTULIN protein function (Table 1). Apart from one intronic variant (Nabavi *et al*, 2019), all published homozygous variants in the *OTULIN* gene (Damgaard *et al*, 2016, 2019; Zhou *et al*, 2016) were located in the OTU domain of OTULIN containing its catalytic activity (Keusekotten *et al*, 2013). In agreement, the two novel variants identified in our study are also localized in the OTU domain (Fig EV2A). In the OTULIN 3D‐structure (3ZNV, 3ZNZ; Keusekotten *et al*, 2013), M86 and W167 are, however, located on different ends of the protein (Fig 3A). The catalytic core of OTULIN is composed of N341, H339, and C129 (Fig 3A; pale cyan) (Rivkin *et al*, 2013). The crystal structure of this catalytic triad was shown to exist in two alternate conformations: “active” and “inhibited” (Keusekotten *et al*, 2013). The coordination of N341 is a key event in OTULIN activation (Keusekotten *et al*, 2013). One of the residues coordinating the N341 side chain is Y91 (Keusekotten *et al*, 2013) (wheat in Fig 3B). Y91, in turn, is involved, through its hydroxyl group, in an intense network of interactions between different OTULIN residues, among them its catalytic residues H339 and N341 (Fig 3B). This network is important for changing OTULIN’s conformation from “inhibitory” to “active” and co‐ordinates the catalytic triad residue N341, aligning it to H339 (Keusekotten *et al*, 2013). Importantly, the aromatic ring of Y91 approaches the sulfur center of M86 to a distance of 4.1 Å (Fig 3B; sulfur in yellow). The sulfur center of sulfur‐containing amino acids (M, C) and aromatic side chains of Y, W, or F are involved in close (< 5 Å) and frequent contacts in proteins (sulfur‐arene interactions) (Meyer *et al*, 2003). This interaction is lost in the mutant M86I (Fig 3B; mutant in pale green). We hypothesize that this loss alters the conformation of the catalytic core of OTULIN, enhancing the proportion of the inactive conformation, and thereby reducing the enzyme’s turnover number (*k_cat_*). Consistent with this notion is the observation that replacement of Y91 by F resulted in a 20‐fold reduction of *k_cat_* while not affecting *K_M_* (Keusekotten *et al*, 2013). Moreover, when in complex with M1‐diubiquitin (3ZNZ), Y91 is one of OTULIN’s S1' contact sites with the F4 patch of proximal ubiquitin (Fig 3C). The interaction between Y91 and M86, which is lost in the mutant M86I, might, thus, also play a role in binding of OTULIN to linear ubiquitin (Fig 3C). The position of the maternal variant W167 is in close proximity to OTULIN’s helical arm containing the S1 contact site with the I44 patch of distal ubiquitin. This helical arm comprises or adjoins the three residues Y244, L272, and G281, replaced in the known homozygous variants of OTULIN (Damgaard *et al*, 2016, 2019, 2020; Zhou *et al*, 2016) (Fig 3D). The mutation of W167 to S results in a replacement of the bulky, apolar indole side chain of W167 by a much smaller, polar hydroxymethyl side chain (Fig 3D; close‐up). It is likely, therefore, that the W167S replacement affects the orientation of the helical arm's S1 site, made up of L259, A262, and R263 toward the I44 patch of distal ubiquitin, thereby indirectly interfering with OTULIN’s binding (*K_M_*) to linear ubiquitin (Fig 3D).

**Table 1:** Compound heterozygous variants in the *OTULIN* gene in a patient with autoinflammation and sterile abscess formation.

	Nucleotide alteration	CDS position	AA alteration	domain	SIFT	PolyPhen
Paternal allele	Chr5: 14678818G>A	c.258G>A	p.M86I	OTU	0.11	0.31
Maternal allele	Chr5: 14687661G>C	c.500G>C	p.W167S	OTU	0	0.998

CDS, coding sequence; AA, amino acid; SIFT, Sorting Intolerant From Tolerant (< 0.05 = deleterious); PolyPhen, Polymorphism Phenotyping (> 0.908 “Probably Damaging”, 0.446–0.908 “Possibly Damaging”, < 0.446 “Benign”).

Collectively, this structural analysis suggests that both substitutions, M86I and W167S, may interfere with binding of OTULIN to linear ubiquitin (Fig 3C and D). Moreover, M86I might additionally reduce OTULIN’s catalytic turnover number (*k_cat_*) (Fig 3B).

### OTULIN variants compromise binding of OTULIN to linear ubiquitin and differentially affect OTULIN’s intrinsic stability and catalytic activity

**Figure 4:**
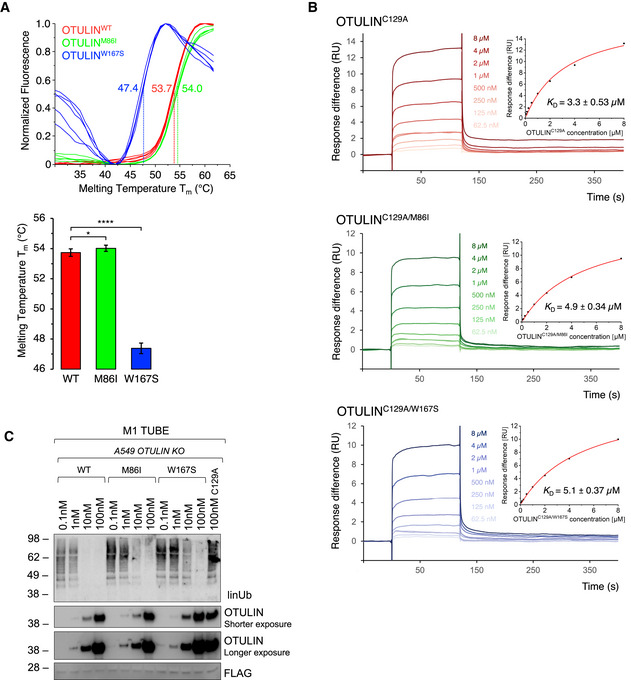
OTULIN variants compromise binding of OTULIN to linear ubiquitin and differentially affect OTULIN’s intrinsic stability and catalytic activity Biochemical characterization of recombinant OTULIN variants by means of differential scanning fluorimetry (DSF) (A) and surface Plasmon resonance (SPR) measurements (B).
DSF measurements with OTULIN^WT^, OTULIN^M86I^, or OTULIN^W167S^. Melting temperatures (T*_m_*) are calculated from five independent experiments with standard deviations. *P = 0.011; ****P = 6.64 × 10^−18^, unpaired *t*‐test.SPR measurements and steady‐state binding curves with calculated dissociation constants (*K_d_*) after injection of a concentration series of OTULIN^C129A^, OTULIN^C129A/M86I^, or OTULIN^C129A/W167S^ to CM5‐immobilized di‐ubiquitin chains.Linear ubiquitin linkages isolated from A549 *OTULIN* KO cells by M1 TUBE assay were incubated with increasing concentrations of recombinant OTULIN^WT^, OTULIN^M86I^, and OTULIN^W167S^ or catalytically inactive OTULIN^C129A^ as control for 1 h. Afterward, samples were subjected to analysis by Western blot for the indicated proteins. Images are representative of three independent experiments.

To assess how the variants p.M86I and p.W167S affect OTULIN’s intrinsic thermal stability, we purified recombinant wildtype (OTULIN^WT^) and variant OTULIN (OTULIN^M86I^ and OTULIN^W167S^) and determined their melting points (T*_m_*) by means of differential scanning fluorimetry (Fig 4A). While OTULIN^WT^ and OTULIN^M86I^ had a similar T*_m_* (OTULIN^WT^: 53.7°C, OTULIN^M86I^: 54°C), OTULIN^W167S^ unfolded at a significantly lower temperature (T*_m_* 47.4°C) in this assay (Fig 4A). These results are in line with the structural observation that W167 is part of the protein’s hydrophobic core and indicates that replacement with a smaller and more hydrophilic side chain of serine (W167S) influences its integrity. In contrast, the structurally more conservative mutation M86I appears to affect the overall conformation of the protein much less.

To measure the effect of the M86I and W167S mutations on the binding affinity of OTULIN to linear di‐ubiquitin chains, surface Plasmon resonance (SPR) measurements were performed. To prevent ubiquitin cleavage during SPR measurements, a catalytically inactive mutant (OTULIN^C129A^) was used. OTULIN^C129A^ bound linear di‐ubiquitin chains with a *K*_d_ of 3.3 µM in this assay, while lower affinities were determined for both variants OTULIN^C129A/M86I^ and OTULIN^C129A/W167S^ (4.9 µM and 5.1 µM, respectively) (Fig 4B).

To further evaluate to which extent OTULIN’s catalytic activity was impaired by the two variants, we performed a DUB assay using recombinant OTULIN^WT^, OTULIN^M86I^, and OTULIN^W167S^ on endogenous linear ubiquitin linkages isolated from A549 *OTULIN* KO cells (Fig 4C). Whereas OTULIN^WT^ was able to cleave all linear ubiquitin chains, residual amounts of linkages were still present in samples incubated with OTULIN^W167S^. Moreover, also in samples treated with OTULIN^M86I^, we detected remains of linear ubiquitin linkages, although to a lesser extent than in OTULIN^W167S^‐treated samples. In summary, we find binding of OTULIN to linear ubiquitin to be compromised by both variants; however, protein stability and catalytic activity is most affected by the OTULIN variant p.W167S.

### Parental variants both contribute to defective hydrolysis of linear ubiquitin linkages in patient‐derived cells, but to different extents

**Figure 5:**
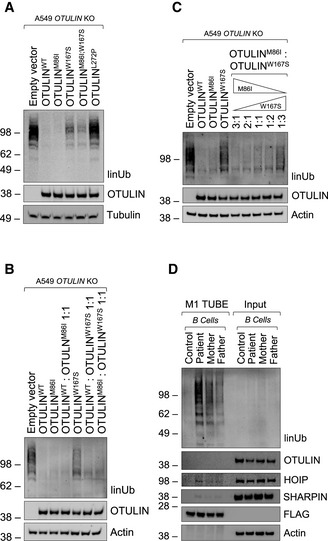
Parental variants both contribute to defective hydrolysis of linear ubiquitin linkages in patient‐derived cells, but to different extents A–CA549 *OTULIN* KO cells were transfected with empty vector or different OTULIN constructs as indicated and analyzed by Western blot.DFLAG‐tagged tandem ubiquitin binding entity (TUBE) assay was performed in B cells from control, patient, mother, and father to pull down linear ubiquitin linkages. One representative (A–D) of three independent experiments is shown. Source data are available online for this figure.

**Figure EV3:**
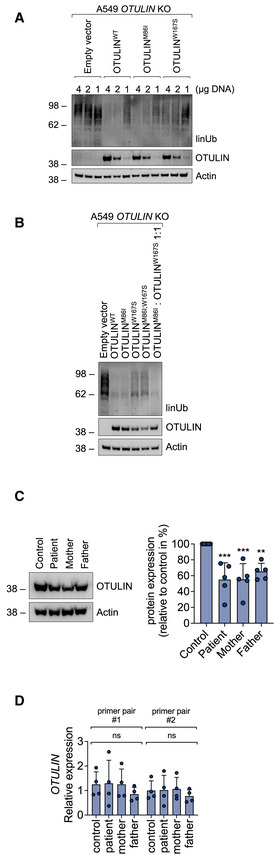
The maternal OTULIN variant p.W167S more severely impairs hydrolysis of linear ubiquitin linkages than the paternal OTULIN variant p.M86I A, BA549 *OTULIN* KO cells were transfected with different OTULIN constructs as indicated. After 24 h, cells were lysed and subjected to analysis by Western blot. One representative of three independent experiments is shown.COTULIN protein expression in B cells derived from control, patient, mother, and father is shown. Blots (left panel) are representative of five independent experiments analyzed by densitometry (right panel). Data are presented as mean ± SD (dots represent individual experiments); ***P = 0.0008, **P = 0.0069, ordinary one‐way ANOVA, Dunnett’s multiple comparisons test.DRelative mRNA expression of *OTULIN* in B cells derived from control, patient, mother, and father determined with two different primer pairs is depicted. Data are presented as mean ± SD of four independent experiments; dots represent individual experiments performed in three technical replicates; ns, non‐significant, ordinary one‐way ANOVA, Dunnett’s multiple comparisons test.

To validate these biochemical findings in a cellular context, we assessed the contribution of both parental variants to defects in hydrolysis of linear ubiquitin linkages in a heterologous cell system. Plasmid vectors encoding wildtype OTULIN (OTULIN^WT^), the paternal p.M86I variant (OTULIN^M86I^) and the maternal p.W167S variant (OTULIN^W167S^) were transiently expressed in A549 *OTULIN* KO cells. Plasmid vectors encoding both parental variants (OTULIN^M86I;W167S^) and the published variant p.L272P (OTULIN^L272P^) (Damgaard *et al*, 2016; Zhou *et al*, 2016) were used for comparison. OTULIN^WT^ and OTULIN^M86I^ were more efficient in hydrolysis of linear ubiquitin linkages than OTULIN^W167S^ or OTULIN^M86I;W167S^ when re‐expressed in OTULIN‐deficient cells (Fig 5A). The amount of linear ubiquitin in cells transfected with OTULIN^L272P^ was similar to empty vector (EV)‐transfected cells (Fig 5A) indicating that OTULIN’s catalytic activity was most compromised by this variant. The compromised DUB activity of OTULIN^W167S^ compared to OTULIN^WT^ and OTULIN^M86I^ was stable over a range of different DNA concentrations (Fig EV3A). As the double‐mutant OTULIN^M86I;W167S^ was less efficient in cleaving linear ubiquitin linkages than a mix of OTULIN^M86I^ and OTULIN^W167S^ (Fig EV3B), we reasoned that OTULIN^M86I^ might be able to substitute for the defect of OTULIN^W167S^. Indeed, defective hydrolysis of linear ubiquitin linkages by OTULIN^W167S^ was rescued by co‐expression of OTULIN^WT^ and, importantly, also by OTULIN^M86I^ (Fig 5B), at least when equal amounts of OTULIN^M86I^ and OTULIN^W167S^ were present in cells. When we next transfected A549 *OTULIN* KO cells with the two constructs OTULIN^M86I^ and OTULIN^W167S^ in different proportions (Fig 5C), we found a surplus of OTULIN^W167S^ to result in increased linear ubiquitin linkages suggesting that OTULIN^M86I^ can rescue the defect of OTULIN^W167S^ only to some extent. To investigate the regulation of linear ubiquitin linkages by the different OTULIN variants in a setting that is close to the natural situation, we assessed linear ubiquitin linkages in EBV‐transformed B cell lines from patient, mother, father and healthy control (Fig 5D). Surprisingly, not only the patient’s B cells but also the mother’s and the father’s B cells had a surplus of linear ubiquitin linkages, although to a lesser extent (Fig 5D). OTULIN protein expression was again diminished in patient‐derived B cells, but, interestingly, also in B cells derived from parents, as compared to control (Figs 5D and EV3C; see Fig 2 for comparison), whereas levels of *OTULIN* mRNA were similar (Fig EV3D). Together, these results suggest that OTULIN’s catalytic activity is compromised by both parental variants, with the maternal variant p.W167S, however, more severely impairing hydrolysis of linear ubiquitin linkages than the paternal variant p.M86I.

### TNF signaling is altered in patient‐derived cells

**Figure 6:**
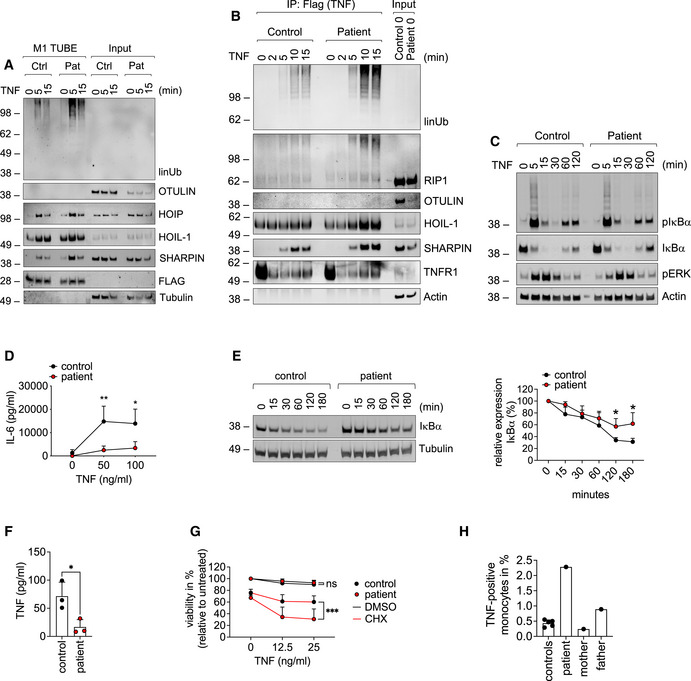
TNF signaling is altered in patient‐derived cells A–DPatient‐derived and control fibroblasts were used. (A) FLAG‐tagged tandem ubiquitin binding entity (TUBE) assay was performed to pull down linear ubiquitin linkages upon stimulation with 500 ng/ml TNF. (B) The TNFR1‐SC was isolated using 500 ng/ml TAP‐TNF. (C) Cells were stimulated with 100 ng/ml TNF for the indicated times and analyzed by Western blot. One representative (A–C) of three independent experiments is shown. (D) Cells were stimulated with TNF for 24 h and IL‐6 was determined by ELISA. Data are presented as mean ± SD of three individual experiments performed in two technical replicates; *P = 0.014; **P = 0.007; multiple *t*‐test corrected for multiple comparisons using the Holm–Sidak method.EB cells were incubated with 50 µg/ml cycloheximide (CHX) for the indicated times and subjected to analysis by Western blot. One representative (left panel) out of 4 individual experiments analyzed by densitometry (right panel) is shown. Data are presented as mean ± SD. *P = 0.02, unpaired *t*‐test.FPatient‐derived and control B cells were cultivated for 24 h and TNF was determined by ELISA. Data are presented as mean ± SD of 3 individual experiments performed in two technical replicates; *P = 0.028; unpaired *t*‐test.GFibroblasts were incubated with 50 µg/ml cycloheximide (CHX) or DMSO and stimulated with TNF as indicated. Viability was measured after 24 h. Data are presented as mean ± SD of 5 individual experiments performed in three technical replicates; ***P = 0.0008, mixed‐effects analysis with Tukey’s multiple comparisons test.HIntracellular TNF in CD11b‐positive cells from five healthy controls, patient (before anti‐TNF treatment), mother, and father was determined by FACS. Source data are available online for this figure.

**Figure EV4:**
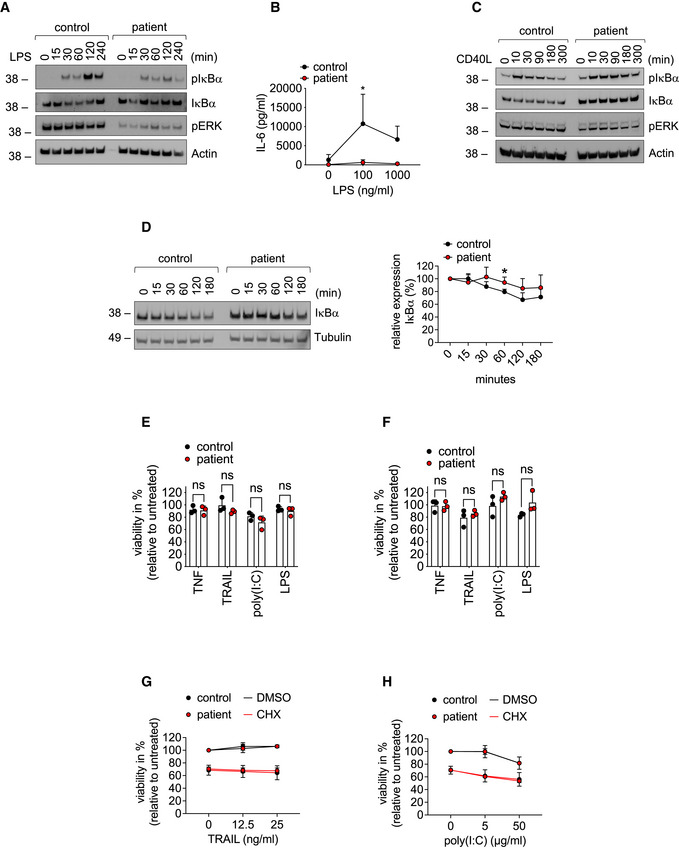
Signaling output of different innate immune stimuli in patient‐derived cells AFibroblasts were stimulated with 1 µg/ml LPS for the indicated times and subjected to analysis by Western blot. One representative out of three independent experiments is shown.BFibroblasts were stimulated with LPS for 24 h. Concentration of IL‐6 in the supernatant was determined by ELISA. Data are presented as mean ± SD of three individual experiments performed in two technical replicates; *P = 0.012; multiple *t*‐tests corrected for multiple comparisons using the Holm–Sidak method.CB cells were treated with 100 ng/ml CD40L for the indicated times and subjected to analysis by Western blot. One representative out of three independent experiments is shown.DFibroblasts were incubated with 50 µg/ml cycloheximide (CHX) for the indicated times and subjected to analysis by western blot. One representative (left panel) out of 4 individual experiments analyzed by densitometry (right panel) is shown. Data are presented as mean ± SD. *P = 0.02, unpaired *t*‐test.E, FFibroblasts (E) or B cells (F) were treated with 0.1 µg/ml TNF, 0.1 µg/ml TRAIL, 100 µg/ml poly(I:C), 1 µg/ml LPS or left untreated, and viability was determined after 24 h. Data are presented as mean ± SD of three independent experiments; dots represent individual experiments performed in three technical replicates; ns, non‐significant, unpaired *t*‐test.G, HFibroblasts were incubated with 50 µg/ml cycloheximide (CHX) or DMSO and stimulated with the indicated concentrations of TRAIL (G) or poly(I:C) (H). Viability was measured after 24 h. Data are presented as mean ± SD of three individual experiments performed in three technical replicates. Source data are available online for this figure.

Both, mice and humans with defects in OTULIN suffer from autoinflammation mediated by TNF receptor 1 (TNFR1) (Damgaard *et al*, 2016, 2019; Heger *et al*, 2018). Importantly, patients with ORAS have successfully been treated with anti‐TNF therapy (Damgaard *et al*, 2016, 2019; Zhou *et al*, 2016) (Table EV1). Thus, we investigated whether the compound heterozygosity in *OTULIN* affected TNF signaling in patient‐derived cells. We found that TNF stimulation resulted in increased formation of linear ubiquitin linkages in patient‐derived fibroblasts as compared to control (Fig 6A). Surprisingly, increased presence of linear ubiquitin linkages and LUBAC components was detectable in the TNFR1‐signaling complex (SC) in patient‐derived cells as compared to control (Fig 6B) although it was recently shown that recruitment of LUBAC to the TNFR1‐SC is compromised when OTULIN’s catalytic activity is perturbed (Heger *et al*, 2018). OTULIN itself was not recruited to the TNFR1‐SC (Fig 6B) in line with published data (Draber *et al*, 2015; Elliott *et al*, 2016; Hrdinka *et al*, 2016). Of note, OTULIN compound heterozygosity only marginally affected nuclear factor kappa B (NF‐κB) and mitogen‐activated protein kinase (MAPK) signaling activation upon TNF stimulation (Fig 6C). Secretion of interleukin‐6 (IL‐6) upon stimulation with TNF, however, was lower in patient‐derived fibroblasts compared to control (Fig 6D). Lipopolysaccharide (LPS) stimulation resulted in diminished activation of NF‐κB signaling (Fig EV4A) along with reduced secretion of IL‐6 (Fig EV4B) in patient‐derived fibroblasts as compared to control. Moreover, IκBα degradation induced by cluster of differentiation 40 ligand (CD40L) was more pronounced in control than in patient‐derived B cells (Fig EV4C). These data are supported by the fact that IκBα, assessed by cycloheximide (CHX) chase analysis, was more stable in patient‐derived B cells (Fig 6E) and fibroblasts (Fig EV4D) as compared to control suggesting basal NF‐κB activity to be diminished in patient‐derived cells. This is in line with the observation that basal TNF levels were lower in supernatants of patient‐derived B cells as compared to control (Fig 6F).

TNF induces cell death under certain circumstances (Peltzer *et al*, 2016). However, patient‐derived fibroblasts or B cells and respective control cells were mostly resistant to cell death induction by TNF or by other innate immune stimuli such as TNF‐related apoptosis‐inducing ligand (TRAIL), poly(I:C) or LPS (Fig EV4E and F). It was reported that variants in *OTULIN* can affect sensitivity of cells to TNF‐induced death in the presence of CHX (Damgaard *et al*, 2019). Of note, in the presence of CHX, TNF‐induced cell death was significantly higher in patient‐derived fibroblasts than in control cells (Fig 6G). Interestingly, however, patient‐derived fibroblasts were not sensitized to cell death induction by TRAIL or poly(I:C) (Fig EV4G and H).

**Figure EV5:**
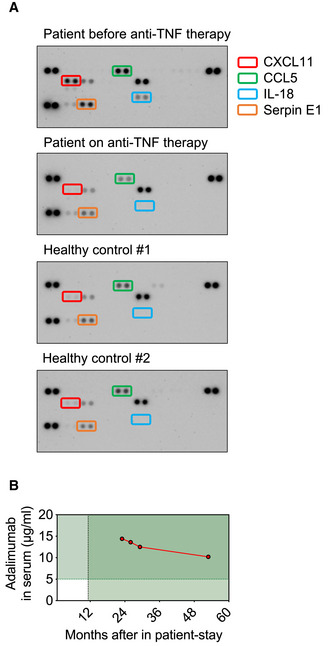
The patient’s inflammatory plasma protein profile is converted to normal after treatment with the anti‐TNF therapeutic Adalimumab Plasma was collected from the patient before and after start of therapy with the anti‐TNF therapeutic Adalimumab. Plasma from two healthy controls was used as comparison. The Proteome Profiler Human Cytokine Array was performed according to the manufacturer’s instructions.The concentration of Adalimumab in the patient’s serum is shown (target concentration: > 5 µg/ml).

Myeloid cells of ORAS patients are suggested to be hyperinflammatory and to show increased production of TNF and other inflammatory mediators (Zhou *et al*, 2016; Damgaard *et al*, 2019). Indeed, patient‐derived monocytes showed higher intracellular TNF staining as compared to monocytes of healthy controls, mother, or father (Fig 6H). ORAS patients were identified to benefit from TNF blockers (Damgaard *et al*, 2016, 2019; Zhou *et al*, 2016) underlining the importance of TNF in the pathogenesis of the disease. Thus, this study’s patient received 30 mg Adalimumab applied subcutaneously every 14 days (Fig 1A) as preventive measure after WES revealed his compound heterozygosity in OTULIN (Fig 1E). The Adalimumab dosage was subsequently adjusted to 40 mg. To assess whether Adalimumab treatment had a beneficial effect on the patient’s inflammatory state, we compared the patient’s plasma protein profile before anti‐TNF treatment and thereafter with plasma from two healthy controls (Fig EV5A). Whereas the patient’s plasma protein profile differed from that of healthy controls before anti‐TNF treatment with upregulation of C‐X‐C motif chemokine ligand (CXCL) 11, C‐C motif chemokine ligand (CCL) 5, IL‐18, and serpin E1, it was reverted to healthy condition after start of anti‐TNF treatment. The Adalimumab concentration in the patient’s serum has always been within therapeutic range (Fig EV5B), and importantly, the patient has not encountered any additional autoinflammatory episodes for 5 years. Laboratory checks and abdominal ultrasound examinations were carried out regularly every 6 months. The liver had initially been unconspicuous on ultrasound; however, the patient has developed signs of liver steatosis over the past 2 years (currently grade II) (Fig EV1B).

## Discussion

Classical OTULIN‐related autoinflammatory syndrome (ORAS) is a rare disease with neonatal‐onset autoinflammation due to homozygous variants in the *OTULIN* gene (Damgaard *et al*, 2016, 2019; Zhou *et al*, 2016; Nabavi *et al*, 2019). We show here that compound heterozygous variants in *OTULIN* result in atypical ORAS with distinct clinical manifestation of the disease. The described patient had a previously unremarkable medical history prior to encountering a fulminant autoinflammatory episode at the age of 7 years presenting with fever and severe sterile abscess formation at multiple organ sites but, importantly, without suffering from diarrhea or arthritis (Table EV1).

At the molecular level, we demonstrate that patient‐derived cells express less OTULIN protein than control cells (Fig 2A) and increased amounts of linear ubiquitin linkages under basal conditions (Fig 2B), and, importantly, also upon stimulation with TNF (Fig 6A), the cytokine identified as primary driver of inflammation in mice and humans with defective OTULIN function (Damgaard *et al*, 2016, 2019; Heger *et al*, 2018). In addition, we find linear ubiquitin linkages to be more abundant in the TNFR1‐SC along with increased presence of LUBAC components as compared to control (Fig 6B). Two separate pools of cytoplasmic LUBAC complexes were identified as abundant in cells (Draber *et al*, 2015): (i) LUBAC in complex with OTULIN, and (ii) LUBAC in complex with CYLD, a DUB known to antagonize K63‐ and linear ubiquitin linkages in SCs (Kovalenko *et al*, 2003), and spermatogenesis‐associated protein (SPATA) 2 (Elliott *et al*, 2016; Kupka *et al*, 2016; Schlicher *et al*, 2016; Wagner *et al*, 2016). The latter complex can be recruited to SCs, including the TNFR1‐SC (Elliott *et al*, 2016; Kupka *et al*, 2016; Schlicher *et al*, 2016; Wagner *et al*, 2016). The binding of SPATA2‐CYLD or OTULIN to HOIP was shown to be mutually exclusive (Draber *et al*, 2015; Schlicher *et al*, 2016). Thus, a downregulation in OTULIN expression levels, potentially due to reduced thermal stability of OTULIN^W167S^ (Fig 4A), as observed in this patient, might result in increased LUBAC‐SPATA2‐CYLD complex formation, and, in turn, increased recruitment of LUBAC to the TNFR1‐SC (Fig 6B). This is an intriguing finding as linear ubiquitin linkages in the TNFR1‐SC were unaffected by a complete absence of OTULIN (Draber *et al*, 2015), and expression of the catalytically inactive OTULIN^C129A^ resulted in even reduced presence of LUBAC and linear ubiquitin linkages in the TNFR1‐SC (Heger *et al*, 2018). OTULIN deficiency or expression of catalytically inactive OTULIN^C129A^ cause increased LUBAC autoubiquitination and degradation (Damgaard *et al*, 2016, 2019; Heger *et al*, 2018; Verboom *et al*, 2020). In patient‐derived cells, LUBAC components were pulled down together with increased amounts of linear ubiquitin linkages in patient‐derived cells (Figs 2B and 6A). Whether LUBAC components are modified by linear ubiquitin linkages in patient‐derived cells, however, requires further investigation. Expression levels of LUBAC components were not altered (Fig 2A) and, in addition, we observed augmented LUBAC recruitment to the TNFR1‐SC (Fig 6B) along with increased ubiquitination of the TNFR1‐SC component RIPK1 (Fig 6B). This is in line with previous reports demonstrating that OTULIN can regulate ubiquitination of substrates other than LUBAC, for example, RIPK1 and TNFR1 (Fiil *et al*, 2013; Keusekotten *et al*, 2013).

Thus, even subtle changes in the expression of OTULIN, LUBAC, and probably also CYLD and SPATA2, and their catalytic activities may affect the composition of the TNFR1‐SC and its signaling output.

Analysis of the OTULIN structure suggested the variants in the described patient to interfere with the protein’s catalytic activity and binding to linear di‐ubiquitin (Fig 3). Our in‐depth biochemical analyses indicate that both variant OTULIN proteins, p.M86I and p.W167S, have reduced binding affinities to linear di‐ubiquitin in SPR experiments (Fig 4B). The OTULIN variant p.W167S, however, is the one with lower thermal stability (Fig 4A) and catalytic activity (Figs 4C and 5). Performing reconstitution experiments in a heterologous cell system, we discovered that the paternal mutation p.M86I can compensate the reduced catalytic activity of p.W167S to a certain extent (Fig 5C). Moreover, the published homozygous OTULIN variant p.L272P (Damgaard *et al*, 2016; Zhou *et al*, 2016) compromised OTULIN’s DUB activity more than the combined action of the OTULIN variants p.M86I and p.W167S (OTULIN^M86I;W167S^) (Fig 5A). Thus, the degree of impairment of linear chain hydrolysis might correlate with ORAS severity and these data could provide the molecular explanation why the patient of this study was supposedly asymptomatic until the age of 7 years while ORAS patients carrying the homozygous OTULIN variant p.L272P diseased already after birth. This idea is in line with the fact that homozygous loss‐of‐function variants in the LUBAC components HOIL‐1 and HOIP were identified to result in a syndrome encompassing autoinflammation and immunodeficiency with variable manifestation depending on the site of the mutation (Boisson *et al*, 2012, 2015; Nilsson *et al*, 2013; Wang *et al*, 2013; Oda *et al*, 2019). Interestingly, the analysis of EBV‐transformed B cells clearly showed that both parental variants affect hydrolysis of linear chains, even in the heterozygous setting, as linear ubiquitin linkages were more abundant in B cells from patient, mother, and father (Fig 5D). This data suggests that even heterozygous variants in the *OTULIN* gene are sufficient to affect the level of linear ubiquitin linkages, at least *in vitro*.

The trigger of the autoinflammatory exacerbation in our patient is currently unknown. Another ORAS patient who carried the homozygous OTULIN variant p.L272P died of pneumococcal septicemia (Damgaard *et al*, 2016). As the urine of our patient was tested positive for pneumococcal antigen (Appendix Table S2), it is tempting to hypothesize that ORAS patients might exhibit increased vulnerability to infections with *S*. *pneumoniae*. This is of particular interest as TNF signaling was demonstrated to play a protective role in the pathogenesis of *S. pneumoniae* infections (Takashima *et al*, 1997; Wellmer *et al*, 2001). Thus, altered TNF signaling might result in enhanced susceptibility of ORAS patients to infections with *S. pneumoniae*. It is therefore reasonable to speculate that they may benefit from pneumococcal vaccinations.

Whereas recently identified patients with ORAS presented with failure to thrive (Damgaard *et al*, 2016, 2019, 2020; Zhou *et al*, 2016), this study’s patient suffers from obesity. Whether the patient’s obesity is due to his compound heterozygosity in OTULIN is currently unknown. Known obesity‐related genes (Appendix Table S8; Chesi & Grant, 2015; Rouillard *et al*, 2016; Warner *et al*, 2021)) were not affected in the patient apart from *FAT1* (FAT atypical cadherin 1) and *WWOX* (WW Domain Containing Oxidoreductase) (Appendix Fig S2). The single nucleotide variants (SNV) described to be associated with obesity are rs9923451 (16: 1677509940) and rs925642 (4: 187915860) for *WWOX* and *FAT1*, respectively (Wang *et al*, 2011). The patient’s *WWOX* and *FAT1* genes, however, showed a heterozygous SNV resulting in a missense mutation of yet unknown clinical impact. Thus, the underlying cause for the patient’s obesity remains currently uncertain.

In addition, one ORAS patient was identified to suffer from progressive steatotic liver disease (Damgaard *et al*, 2020) suggesting that OTULIN is crucial for liver homeostasis. This assumption was confirmed in mice with liver‐specific OTULIN deficiency which suffered from steatohepatitis, fibrosis, and hepatocellular carcinoma (Damgaard *et al*, 2020; Verboom *et al*, 2020). Although our patient showed elevated liver enzymes during his autoinflammatory episode (Fig EV1A), the laboratory values normalized in the further course and always remained within the normal range during follow‐up. Routine abdominal ultrasound examination, however, revealed that the patient had since developed sonographic alterations of the liver indicative of grade II hepatic steatosis (Fig EV1B) while liver function tests remained normal. Whether the steatosis is caused by the patient’s obesity, a risk factor for liver steatosis (Guzzaloni *et al*, 2000), or directly related to his ORAS, and further fueled by his treatment with Adalimumab (Haas *et al*, 2017), cannot be clarified with certainty at the moment. That hepatocytes are vulnerable to changes in the amount of linear ubiquitination is further highlighted by the fact that liver tumorigenesis is also promoted in mice with liver‐specific deletion of HOIP (Shimizu *et al*, 2017). Future studies will be required to assess how exactly the interplay of OTULIN and LUBAC determines liver health to be able to predict whether OTULIN variants identified in this patient, and others, bear the risk of increased liver tumorigenesis. In any case, ORAS patients should be examined regularly for the development of morphological and functional liver abnormalities.

In summary, we identified compound heterozygous variants in the *OTULIN* gene to be responsible for an atypical late‐onset ORAS phenotype with severe autoinflammation and sterile abscess formation in a hitherto supposedly asymptomatic patient. We identified variants in the *OTULIN* gene to affect OTULIN protein expression and to result in increased linear ubiquitin linkage formation under basal conditions, and, importantly also upon stimulation with TNF in the TNFR1‐SC. Whereas the symptoms identified in the patient are distinct from symptoms identified in patients with classical ORAS, we found similarities between classical and atypical ORAS on the molecular level: Monocytes showed enhanced TNF production as reported previously, confirming that TNF secreted from myeloid cells is the primary driver of the inflammation in patients with ORAS (Damgaard *et al*, 2016, 2019). Moreover, TNF‐induced gene activation was downregulated, but TNF‐mediated cell death enhanced in the presence of CHX in patient‐derived fibroblasts, in line with previous reports (Damgaard *et al*, 2016, 2019; Zhou *et al*, 2016).

Our study illustrates that the spectrum of patients with ORAS is wider than so far reported. Upon encountering certain triggers, previously asymptomatic individuals—like the patient described here—may develop a severe and life‐threatening ORAS phenotype.

## Materials and Methods

### Antibodies

Phospho‐IkBα (9246, IgG1, Cell Signaling Technology (CST), dilution 1:1,000), IκBα (9242, rabbit, CST, dilution 1:1,000), phospho‐ERK (9101, rabbit, CST, dilution 1:2,000), MCL‐1 (94296, rabbit, CST, dilution 1:1,000), Tubulin (2148, rabbit, CST, dilution 1:1,000), Tubulin (12004166, Rhodamine‐labelled, Bio‐Rad, dilution 1:5,000), OTULIN (14127, rabbit, CST, dilution 1:2,000), OTULIN (ab151117, rabbit, Abcam, dilution 1:2,000), Actin (A1987, IgG1, Sigma‐Aldrich (SA), dilution 1:5,000), FLAG (F3165, IgG1, SA, dilution 1:1,000), HOIP (SAB2102031, rabbit, SA, dilution 1:1,000), SHARPIN (14626‐1‐AP, rabbit, Proteintech, dilution 1:2,000), TNFR1 (sc‐8436, IgG2b, Santa Cruz, dilution 1:1,000), RIP1 (610459, IgG2a, BD Bioscience, dilution 1:1,000). Antibody against HOIL‐1 (IgG2a) was previously described (dilution 1:2,000) (Haas *et al*, 2009; Gerlach *et al*, 2011). Antibody detecting linear ubiquitin (rabbit) was generated using amino acid sequences as described previously (dilution 1:1,000) (Matsumoto *et al*, 2012). In brief, FreeStyle CHO‐S cells (Thermo Fisher) were transfected with 100 µg of plasmid (pVITRO1‐Rabbit‐Ubiq‐Ab) using Amaxa Nucleofector. 5 days after transfection, the supernatant was collected, purified with a protein A/G column, and eluted with 0.2 M glycine. 40% of glycerol was added to the eluate before storage at −20°C. pVITRO1‐trastuzumab (Addgene, 61883) was used as backbone plasmid, in which the signal peptide of IL‐2 was added and the variable region substituted with the sequence of interest. Plasmid sequence information is available upon request.

### Cell lines and materials

The ethics committee of Ulm University provided guidelines for study procedures. The study complies with relevant ethical regulations. Written informed consent was obtained from all subjects. The patient’s parents gave written informed consent to this study and its publication. This study is a case report with an individual attempt at healing. The principles set out in the WMA Declaration of Helsinki and the Department of Health and Human Services Belmont Report have been respected where applicable. Epstein–Barr virus (EBV)‐transformed B cell lines were generated at the Institute of Virology, Ulm University Medical Center, Ulm, Germany. B cell lines were cultured in RPMI 1640 containing 20% FCS, 2% l‐Glutamine and 1% Penicillin/Streptomycin (P/S). Normal Human Dermal Fibroblasts (NHDF) were obtained from Cambrex Bio Science and patient‐derived fibroblasts were generated at the Department of Pediatrics and Adolescent Medicine, Ulm University Medical Center, Ulm, Germany. Fibroblasts were cultured in DMEM (ATCC, 30‐2002) containing 20% FCS and 1% P/S. A549 cells were purchased from Caliper Life Science. A549 *OTULIN* KO and control cells were derived as previously described (Draber *et al*, 2015) and cultured in Ham’s F‐12K (Kaighn’s)‐Medium containing 10% FCS and 1% P/S. All cell lines used in this study were determined to be free of mycoplasma using MycoAlert Mycoplasma Detection kit (Lonza). Cell lines were not authenticated. Biological material derived from patient and parents is restricted.

### Genomic sequencing

Whole exome sequencing of the patient's genomic DNA was performed using an Illumina sequencing platform. Bioinformatics analysis for detection of rare sequence variants following Mendelian inheritance patterns were performed as described previously (Field *et al*, 2015). Confirmation of the segregation of the OTULIN variants considered to be pathogenic was obtained by Sanger sequencing of genomic DNA (ABI 3130XL; Thermo Fisher) with the use of Big Dye Terminator (v.1.1) chemistry (Thermo Fisher). Primers used for sequencing were designed using ENSG00000154124.5 as reference sequence.

### Histology

Tissue was fixed in 5% buffered formalin (pH 7,4) for 24 h. Paraffin sections of about 3 µm were stained with hematoxylin–eosin; immunohistochemistry with a monoclonal antibody specific for smooth muscle actin (Clone HHF35, IR700, Dako Denmark) was used according to standard protocols on a Dako stainer (Wildermann *et al*, 2021).

### 3D structural modeling

The 3D structures of OTULIN variants were predicted by protein structure homology‐modeling using SWISS‐MODEL (Waterhouse *et al*, 2018) using the known structures 3ZNV and 3ZNZ (Keusekotten *et al*, 2013) as templates. The structures were visualized and analyzed using the PyMOL software (version 2.4.1; Schroedinger).

### Cloning

Wildtype OTULIN DNA was amplified from DNA, and the PCR product was cloned into the BamHI and XhoI site of pcDNA3.1+. Point mutations in the OTULIN‐DNA to generate DNA encoding variants OTULIN^M86I^, OTULIN^W167S^, OTULIN^M86I;W167S^, and OTULIN^L272P^ were introduced by site‐directed mutagenesis according to the manufacturer’s instructions (QuikChange II Site‐Directed Mutagenesis Kit, Agilent). Nucleotide sequence information of the primers used for site‐directed mutagenesis is available upon request.

For protein expression the DNA encoding wildtype OTULIN, OTULIN^C129A^, OTULIN^M86I^, OTULIN^W167S^, OTULIN^M86I/C129A^, and OTULIN^W167S/C129A^ were cloned into the BamHI and XhoI in a modified pGEX‐4T‐2 (kindly provided by Michael Meister) carrying a sequence encoding a HRV3C protease site. The GST‐OTULIN proteins were expressed in *E. coli* strain BL21 (DE3). Cells were grown at 37°C in LB medium containing 50 μg/ml ampicillin to an OD_600_ of 0.8. The culture was cooled to 20°C prior to induction with 400 μM IPTG and harvested 20 h post‐induction.

### Purification of OTULIN

For purification of GST‐tagged OTULIN^WT^, OTULIN^C129A^, OTULIN^M86I^, OTULIN^W167S^, OTULIN^M86I/C129A^, and OTULIN^W167S/C129A^, cells were resuspended in lysis buffer (500 mM NaCl, 50 mM Tris–HCl pH 8.0, 2 mM DTT) and disrupted using an LM10 microfluidizer (Microfluidics). The clarified lysate (30,000 *g*, 30 min, 4°C) containing GST‐OTULIN was applied onto equilibrated GSH‐Sepharose 4B beads (Cytiva) and unbound proteins were removed by washing with 2 column volumes of lysis buffer. OTULIN was released by on‐column cleavage with 0.1 mg HRV3C protease overnight, collected and concentrated using Amicon Ultra Centrifugal Filter Units (Merck). A final polishing step was performed by gel filtration (Superdex 200 XK 16/600, Cytiva) in a buffer containing 150 mM NaCl, 20 mM Tris–HCl pH 8.0, and 2 mM DTT. OTULIN containing fractions were pooled, concentrated, frozen in liquid nitrogen, and stored at −80°C.

### Binding assay

Binding between OTULIN^C129A^ (catalytically inactive background), OTULIN^M86I/C129A^ or OTULIN^W167S/C129A^ and di‐ubiquitin chains was analyzed using a Biacore S200 instrument (Cytiva). Di‐ubiquitin chains (UbiQ) were amine‐coupled to the CM5 sensor chip (Cytiva) at a concentration of 10 µg/ml in 10 mM sodium acetate buffer pH 4.0. Concentration series of OTULIN^C129A^, OTULIN^M86I/C129A^, or OTULIN^W167S/C129A^ (0.065, 0.125, 0.25, 0.5, 1, 2, 4, and 8 µM) were injected in a multi‐cycle approach onto the sensor chip at a flow rate of 30 µl/min in running buffer (150 mM NaCl, 20 mM HEPES/NaOH pH 7.5, 2 mM DTT, 0.005% Tween 20) at 20°C. The surface was regenerated with 10 mM Glycine pH 2.2 between the runs. Acquired binding curves were double‐referenced against buffer runs and a ligand‐free reference channel. The equilibrium dissociation constants (*K*_d_) were calculated from steady‐state measurements using the BIAevaluation program (Cytiva).

### Differential scanning fluorimetry (DSF)

OTULIN^WT^, OTULIN^M86I^, and OTULIN^W167S^ were subjected to thermal denaturation in a real‐time thermal cycler (qTOWER^3^ G touch, Analytik Jena) using emission and excitation wavelengths of 490 and 580 nm, respectively. Quintuplicates were measured in 2× SYPRO^®^‐Orange (SA) at a protein concentration of 4 µM in gel filtration buffer. Experiments were performed in white 96‐well qPCR plates (Sarstedt), which were sealed with adhesive qPCR seal (Sarstedt) and centrifuged at 1,000 *g* for 1 min and 20°C to remove air bubbles. A temperature gradient from 30 to 90°C was applied with a ramping rate of 1°C/s and a ΔT of 1°C while fluorescence was detected after an equilibration time of 30 s. Curves were referenced against buffer controls and T*_m_* values calculated and averaged from the derivatives of the melting curves (‐dRn/dT) as implemented in qPCRsoft 4.0 (Jena Analytik).

### Intracellular TNF staining in monocytes

4 × 10^6^ Ficoll‐isolated PBMCs were seeded in a 24‐well plate in 2 ml RPMI‐1640 (+ 10% FCS, 2 mM l‐glutamine, 1 mM Sodium‐Pyruvate, 50 μM β‐Mercaptoethanol) for 4 h in presence of 10 µg/ml Brefeldin A (B7651, SA). Cell surface was stained using CD11b‐PB antibody (dilution 1:200; 301315, BioLegend) for 20 min at 4°C in the dark. Intracellular TNF was stained using TNF‐PE (dilution 1:20; 502909, BioLegend) or IgG1‐PE (dilution 1:20, 400140, BioLegend) as isotype control for 30 min at 4°C in the dark.

### Western blot

Cells were lysed in lysis buffer (30 mM Tris–HCl, pH 7.4, 150 mM NaCl, 2 mM EDTA, 2 mM KCl, 10% Glycerol) supplemented with 1% Triton X‐100, 1× complete EDTA‐free protease‐inhibitor mix (Roche), and 1× phosphatase‐inhibitor cocktail 2 (SA). Lysates were denatured in reducing sample buffer before separation by SDS–PAGE (Bolt^TM^ 4–12% Bis‐Tris). Membranes were incubated with primary antibodies at 4°C overnight or for 1 h at room temperature (RT). Washing of membranes was performed in 1xPBS containing 0.1% Tween‐20 (SA) for 3 × 10 min prior to incubation with the HRP‐conjugated or StarBright Blue 700 fluorescent secondary antibody for 1 h at RT. Membranes were subsequently developed on a Protec Optimax developing machine or imaged on a ChemiDoc Imaging System.

### Transfection

A549 *OTULIN* KO cells were seeded and transfected on the following day with OTULIN constructs using Lipofectamine 2000 according to the manufacturer’s instructions. After 24 h, cells were lysed and subjected to further experimental analysis.

### TNFR1‐SC analysis

Cells were seeded and stimulated with FLAG‐TNF (Draber *et al*, 2015) as indicated in DMEM with 20% FCS. Cells were washed twice with ice‐cold PBS and harvested in 0.5 ml lysis buffer (100 mM NaCl, 40 mM Tris–HCl, pH 7.5, 1 mM CaCl_2_, 1 mM MgCl_2_, and 1× complete EDTA‐free protease‐inhibitor mix) supplemented with 0.5% Triton X‐100. Lysates were cleared by centrifugation at 18,000 *g* for 20 min. The remaining pellet was resuspended in 0.5 ml of lysis buffer supplemented with 1% Triton X‐100 and 0.1% SDS and placed into an ultrasonic bath for 2 × 20 sec prior to centrifugation at 18,000 *g* for 20 min. Lysates of centrifugation step 1 and 2 were combined. For immunoprecipitation of FLAG‐TNF and associated proteins, 10 µl M2 beads (A2220, SA) were added to each sample and incubated on an overhead wheel at 4°C overnight. Samples were washed at least five times with lysis buffer containing 1% Triton X‐100. After the last centrifugation step, beads were sucked dry and resuspended in 2× reducing sample buffer to elute proteins. Eluted proteins were separated by SDS–PAGE before being examined by Western blotting.

### RNA isolation and qRT–PCR

RNA was isolated with the RNeasy Mini Kit (Qiagen) according to the manufacturer’s instructions. cDNA was synthesized using SuperScript II Reverse Transcriptase (Thermo Fisher) with random primers (Thermo Fisher). qRT–PCR was performed using SsoAdvanced Universal SYBR Green Supermix (Bio‐Rad) on a Bio‐Rad CFX Connect Real‐Time PCR Detection System with the following protocol: 95°C for 30 s, then 40 cycles of 95°C for 5 s followed by 60°C for 30 s.

The following primer sequences were used:

*OTULIN #1*: TATTGACCGCTTCCGTCTTGC (f), GGGTGAGGAGGTGAGACAGAA (r).

*OTULIN #2:* GGGGAAGATCCTCCAGACCT (f), CACACCAAACCTCTTTGCGG (r).

*GAPDH*: GAAGGTGAAGGTCGGAGTC (f), GAAGATGGTGATGGGATTTC (r).

### ELISA

Cells were stimulated as indicated for 24 h and supernatants were analyzed with DuoSet ELISA for human IL‐6 (DY206, R&D Systems) according to the manufacturer's instructions.

### Cell viability assay

Cell viability was determined using CellTiter‐Glo^®^ assay (G7572, Promega) according to the manufacturer’s instructions.

### M1 TUBE

Anti‐M1 TUBE (FLAG, UM606, Life Sensors) was performed according to the manufacturer’s instructions.

### DUB assay

DUB assay was performed as previously described (Draber *et al*, 2015). In brief, M1‐linked ubiquitin chains were isolated from A549 *OTULIN* KO cells with anti‐M1 TUBE (FLAG, UM606, Life Sensors) according to the manufacturer’s instructions. After washing, dried M2‐beads were resuspended in DUB‐buffer (50 mM HEPES (pH 7.6), 150 mM NaCl, 5 mM DTT) and incubated with recombinant OTULIN^WT^, OTULIN^M86I^, or OTULIN^W167S^ at 37°C for 1 h. Reducing sample buffer was added to stop the reaction and proteins were eluted by boiling the beads for 10 min at 90°C. Samples were then further subjected to analysis by Western blot.

### Human cytokine array

The Proteome Profiler Human Cytokine Array Kit (ARY005B, R&D) was performed according to the manufacturer’s instructions.

### Statistical analysis

Data were analyzed using GraphPad Prism software (version 9.1.2) (San Diego, CA). Statistical significance between groups was determined using appropriate statistical tests. For comparison of two groups, unpaired *t*‐test or multiple *t*‐tests corrected for multiple comparisons using the Holm–Sidak method were applied. For comparison of more than two groups, ordinary one‐way ANOVA with Dunnett’s multiple comparisons test or mixed‐effects analysis with Tukey’s multiple comparisons test were used. Normal (Gaussian) distribution of data was assumed. As the sample size was small (always *n* < 7 biological replicates), this assumption was tested using Shapiro–Wilk test. In all quantitative results, standard deviation (SD) is reported to show the variation around the mean. Equal standard deviations were assumed when performing the statistical analysis. Exact p values are stated in the figure legends.

## Author contributions

**Julia Zinngrebe:** Conceptualization; Investigation; Writing—original draft; Writing—review & editing. **Barbara Moepps:** Investigation. **Thomas Monecke:** Investigation. **Peter Gierschik:** Investigation. **Ferdinand Schlichtig:** Investigation. **Thomas Barth:** Investigation. **Gudrun Strauss:** Investigation. **Elena Boldrin:** Investigation. **Carsten Posovszky:** Investigation. **Ansgar Schulz:** Investigation. **Ortraud Beringer:** Investigation. **Eva Rieser:** Investigation. **Eva‐Maria Jacobsen:** Investigation. **Myriam Lorenz:** Investigation. **Klaus Schwarz:** Investigation. **Ulrich Pannicke:** Investigation. **Henning Walczak:** Investigation. **Dierk Niessing:** Investigation. **Catharina Schuetz:** Conceptualization; Investigation. **Pamela Fischer‐Posovszky:** Conceptualization; Investigation; Writing—original draft; Writing—review & editing. **Klaus‐Michael Debatin:** Conceptualization; Writing—original draft; Writing—review & editing.

In addition to the CRediT author contributions listed above, the contributions in detail are:

JZ, CS, PF‐P, and K‐MD conceived the study; JZ, BM, TM, PG, FS, TFEB, GS, and EB performed research; CP, AS, OB, E‐MJ, MRL, KS, and UP provided patient samples and data; ER and HW provided reagents; JZ, BM, TM, PG, FS, TB, GS, EB, CP, OB, ER, MRL, KS, UP, HW, DN, CS, PF‐P, K‐MD analyzed and interpreted data; JZ, PF‐P, and K‐MD co‐wrote the manuscript. All authors read and approved the manuscript.

## For more information

- The portal for rare diseases and orphan drugs: https://www.orpha.net/
- The human gene database: https://www.genecards.org/
- MedlinePlus: https://medlineplus.gov/genetics/condition/otulipenia/
- Genomics England PanelApp: https://panelapp.genomicsengland.co.uk/

## Supporting information



Appendix

Expanded View Figures PDF

Table EV1

Source Data for Expanded View

Source Data for Figure 2

Source Data for Figure 5

Source Data for Figure 6

## Data Availability

Explicit consent for disclosure of entire WES data sets in a public data base had not been obtained. Therefore, there are no such data deposited in external repositories.
