# Supplementary material for: Compound heterozygous variants in OTULIN are associated with fulminant atypical late‐onset ORAS
Source: EMBO Mol Med. 2022 Feb 16;14(3):e14901. doi: 10.15252/emmm.202114901 (PMC8899767; doi:10.15252/emmm.202114901)
Supplement: Supplementary file 1 — Appendix [file EMMM-14-e14901-s006.pdf]

APPENDIX

Compound heterozygous variants in *OTULIN* are associated with fulminant atypical late-onset ORAS

Julia Zinngrebe,[1] Barbara Moepps,[2] Thomas Monecke,[3] Peter Gierschik,[2] Ferdinand Schlichtig,[1] Thomas FE Barth,[4] Gudrun Strauß,[1] Elena Boldrin,[1] Carsten Posovszky,[1] Ansgar Schulz,[1] Ortraud Beringer,[1] Eva Rieser,[5] Eva-Maria Jacobsen,[1] Myriam Ricarda Lorenz,[6] Klaus Schwarz,[6,7] Ulrich Pannicke,[6] Henning Walczak,[5,8] Dierk Niessing,[3] Catharina Schuetz,[9#] Pamela Fischer-Posovszky,[1*#] and Klaus-Michael Debatin,[1#]

[1]Department of Pediatrics and Adolescent Medicine, Ulm University Medical Center, 89075 Ulm, Germany

[2]Institute of Pharmacology and Toxicology, Ulm University, 89081 Ulm, Germany

[3]Institute of Pharmaceutical Biotechnology, Ulm University, 89081 Ulm, Germany

[4]Department of Pathology, University Medical Center Ulm, 89081 Ulm, Germany

[5]Institute of Biochemistry I & CECAD Research Center, University of Cologne, 50931 Cologne, Germany

[6]Institute for Transfusion Medicine, Ulm University, 89081 Ulm, Germany

[7]Institute for Clinical Transfusion Medicine and Immunogenetics Ulm, German Red Cross Blood Service Baden-Wuerttemberg – Hessen, 89081 Ulm, Germany

[8]UCL Cancer Institute, London WC1E 6BT, UK

[9]Pediatric Immunology, Technical University Dresden, 01307 Dresden, Germany

[#]These authors contributed equally.

[*]Corresponding author: Professor Pamela Fischer-Posovszky, PhD

pamela.fischer@uniklinik-ulm.de

Table of content

Appendix Table S1. Microbiological results of patient-derived biopsies. 3

Appendix Table S2. Additional microbiological results of patient-derived material.................4

Appendix Table S3. Histopathological analysis of patient biopsies. 5

Appendix Table S4. Homozygous SNVs 6

Appendix Table S5. Heterozygous SNVs 7

Appendix Table S6. Homozygous small insertions and deletions 9

Appendix Table S7. Heterozygous small insertions and deletions 10

Appendix Table S8. Obesity Gene Sets 11

Appendix Figure S1. Filtering strategy WES 12

Appendix Figure S2. Venn's diagram comparing patient variants with genes known to be involved in obesity and childhood obesity, respectively..13

References 14

Appendix Table S1. Microbiological results of patient-derived biopsies.
This table summarizes all biopsies, intraoperative and wound smears obtained from the patient during his severe inflammatory episode (in days of in-patient stay).

**Biopsies**		
Day 9	Right forearm	Mycobacteria not detected.
Day 9	Right forearm	No pathogen detected.
Day 12	Left forearm	No pathogen detected.
Day 12	Lung	No pathogen detected.
Day 13	Periosteum	No pathogen detected.
Day 13	Skin	No pathogen detected.
Day 13	Fascia	No pathogen detected.
Day 13	Subcutaneous adipose tissue	No pathogen detected.
Day 13	Intercostal muscles	No pathogen detected.
Day 13	Muscle	No pathogen detected.
Day 13	Pleura	No evidence of bacterial or fungal DNA by PCR.
Day 13	Subcutaneous adipose tissue	No evidence of bacterial or fungal DNA by PCR.
Day 13	Intercostal muscles	No evidence of bacterial or fungal DNA by PCR.
Day 13	Muscle	No evidence of bacterial or fungal DNA by PCR.
Day 13	Lung	Mycobacteria not detected.
Day 17	Lung	No evidence of bacterial or fungal DNA by PCR.
Day 30	Spleen	No pathogen detected.
**Intraoperative Smears**		
Day 9	Right wrist	No pathogen detected.
Day 12	Intrathoracic	No pathogen detected.
Day 19	Axilla	No pathogen detected.
Day 19	Axilla	No pathogen detected.
Day 19	Axilla	No pathogen detected.
Day 19	Intraabdominal	No pathogen detected.
**Wound Smears**		
Day 12	Left forearm	No pathogen detected.
Day 32	Thoracic drainage	No pathogen detected.
Day 32	Thoracic drainage	No pathogen detected.
Day 32	Thoracic drainage	No pathogen detected.
Day 34	Axilla	No pathogen detected.
Day 38	Thoracic drainage	No pathogen detected.
Day 38	Axilla	No pathogen detected.
**Pleura Punctuates**		
Day 12	Right	No pathogen detected.
Day 12	Left	No pathogen detected.
Day 13	Intraoperative	No pathogen detected.
**Drainage Tips**		
Day 26	Salem drainage (liver)	No pathogen detected.
Day 26	Jackson Pratt drain (spleen)	No pathogen detected.
Day 27	Douglas	No pathogen detected.
Day 32	Thoracic drainage left	No pathogen detected.

Appendix Table S2. Additional microbiological results of patient-derived material. This table contains all blood, urine, and stool cultures, anal and throat swabs and serologies obtained from the patient during his severe inflammatory episode (in days of in-patient stay).

**Blood Cultures**		
Day 1	Blood culture	No pathogen detected.
Day 6	Blood culture	No pathogen detected.
Day 12	Blood culture (central catheter)	No pathogen detected.
Day 12	Blood culture (central catheter)	No pathogen detected.
Day 13	Blood culture (central catheter)	No pathogen detected.
Day 13	Blood culture (arterial)	No pathogen detected.
Day 26	Blood culture	No pathogen detected.
Day 26	Blood culture	No pathogen detected.
Day 26	Blood culture	No pathogen detected.
Day 26	Blood culture (central catheter)	No pathogen detected.
Day 27	Blood culture (central catheter)	No pathogen detected.
**Urine Cultures**		
Day 12	Midstream urine	Legionella antigen not detected.
Day 16	Catheter urine	Legionella antigen not detected.
Day 16	Catheter urine	Pneumococcal antigen detected.
Day 26	Catheter urine	No fungal or bacterial pathogen detected.
**Stool Cultures**		
Day 1	Salmonellae, Shigellae, Campylobacter and Yersiniae not detected. EHEC negative.	
Day 4	Salmonellae, Shigellae, Campylobacter and Yersiniae not detected.	
Day 8	Salmonellae, Shigellae, Campylobacter and Yersiniae not detected. EHEC negative. Clostridium difficile negative. Worm eggs not detected. Amoebas not detected.	
**Anal Swabs**		
Day 14	Methicillin-resistant staphylococcus aureus not detected.	
Day 15	Physiological flora	
**Throat Swabs**		
Day 1	Physiological flora	
Day 14	Methicillin-resistant staphylococcus aureus not detected.	
**Tracheal Secretion**		
Day 13	No pathogen detected.	
Day 15	No pathogen detected.	
Day 23	No pathogen detected.	
**Serology**		
Day 4	Aspergillus antigen negative, no leptospirosis, no antibodies against Yersiniae.	
Day 10	Campylobacter negative, Coxiella negative, Mycoplasma pneumoniae negative, no antibodies against Francisella tularensis.	
Day 17	Bartonella henselae negative, Bartonella quintana negative, Cryptococcus neoformans antigen not detected, Leishmania negative.	
**Quantiferon Test**		
Day 2	No T cell activation, evaluation not possible.	
Day 3	Infection with M. tuberculosis unlikely.	
Day 4	No T cell activation, evaluation not possible.	
Day 6	No T cell activation, evaluation not possible.	

Appendix Table S3. Histopathological analysis of patient biopsies.
This table summarizes all histologic findings in tissue analyses of the patient during the severe inflammatory episode (in days of in patient-stay).

**Histology**		
Day 10	Right forearm	Inflammation with abscess formation and infiltrating histiocytes. No detection of acid-proof rod bacteria. No indication of gram-positive bacteria. No detection of fungal infection (PAS and Grocott staining). Many cells stain CD15-positive consistent with neutrophil granulocytes. S100 staining identifies some dendritic cells in dermis and epidermis. CD68 staining identifies macrophages within the inflammation. CD1a staining identifies Langerhans cells in epidermis.
Day 12	Skin	Inflammation with tissue necrosis.
	Lung, pleura, intercostal muscles	Massive inflammatory pneumonia with tissue abscess formation. No indication of fungal infection (PAS and Grocott staining negative). Gram staining negative, no indication of pneumococci.
Day 24	Diaphragm	Tissue necrosis.
	Pancreas	Tissue necrosis.
	Spleen	Microfocal abscess formation. No fungal or other germs detectable (PAS, Grocott, EvG, Warthin-starry staining). Gram staining negative, no detection of pneumococci.
	Omentum	Chronic granulating and necrotizing inflammation.
	Left Axilla	Tissue necrosis.
Day 76	Duodenum	No pathologies.
	Duodenum	No pathologies.
	Antrum	Mucous membrane fibrosis and foveolar hyperplasia, minimal chronic uncharacteristic inflammation.

Appendix Table S4. Homozygous SNVs

This table summarizes all homozygous single nucleotide variants (SNVs) identified in the patient by whole exome sequencing (WES).

Chromosome	Position	Reference allele	Variant allele	Gene	AA change	AA position of variant	AA of the total protein	Type of variant
X	147019682	T	C	FMR1	N/A	N/A	N/A	SPLICE (2)
X	46918480	T	C	JADE3	N/A	N/A	N/A	SPLICE (1)

Appendix Table S5. Heterozygous SNVs

This table summarizes all heterozygous single nucleotide variants (SNVs) identified in the patient by whole exome sequencing (WES). Identified OTULIN variants are indicated in blue.

Chromosome	Position	Reference allele	Variant allele	Gene	AA change	AA position of variant	AA of the total protein	Type of variant
2	220080858	G	A	ABCB6	Q->Stop	339	842	NONSENSE
10	101563783	C	G	ABCC2	T->S	406	1545	MISSENSE
1	167829118	C	T	ADCY10	R->Q	608	1610	MISSENSE
11	62295104	T	A	AHNAK	E->V	2262	5890	MISSENSE
14	105417142	T	C	AHNAK2	D->G	1549	5795	MISSENSE
15	85400817	C	T	ALPK3	R->W	1152	1907	MISSENSE
7	36459920	A	G	ANLN	Y->C	671	1124	MISSENSE
19	17444526	T	A	ANO8	N->Y	64	1232	MISSENSE
4	41016023	T	C	APBB2	K->E	138	759	MISSENSE
1	16532517	C	T	ARHGEF19	V->M	454	802	MISSENSE
13	113460599	A	G	ATP11A	I->V	109	1134	MISSENSE
17	79166379	A	G	AZI1	L->P	815	1080	MISSENSE
11	117161764	G	A	BACE1	T->M	315	501	MISSENSE
2	160239106	G	T	BAZ2B	D->E	1323	2168	MISSENSE
5	137505003	T	C	BRD8	M->V	184	1235	MISSENSE
18	51904564	G	A	C18orf54	R->H	356	372	MISSENSE
19	12845130	G	T	C19orf43	S->R	114	176	MISSENSE
11	73834121	G	A	C2CD3	S->F	426	1963	MISSENSE
12	94772596	C	G	CCDC41	E->Q	258	701	MISSENSE
16	3080767	C	T	CCDC64B	R->Q	182	508	MISSENSE
15	59399777	C	G	CCNB2	P->A	59	398	MISSENSE
15	101016370	G	A	CERS3	S->F	177	383	MISSENSE
15	65459039	T	C	CLPX	K->R	148	633	MISSENSE
16	1716088	C	T	CRAMP1L	R->W	923	1269	MISSENSE
9	131857753	G	A	CRAT	R->C	602	626	MISSENSE
11	66276627	G	A	CTD-3074O7.11,DPP3	D->N	707	737	MISSENSE
11	107328536	T	C	CWF19L2	T->A	3	894	MISSENSE
17	76705764	G	A	CYTH1	R->W	25	398	MISSENSE
9	135537967	C	A	DDX31	R->M	169	851	MISSENSE
8	142161835	C	T	DENND3	P->S	245	1198	MISSENSE
19	10906076	G	A	DNM2	R->Q	386	870	MISSENSE
8	21768299	C	T	DOK2	G->E	168	412	MISSENSE
3	113850232	G	C	DRD3	P->A	247	400	MISSENSE
5	31526793	G	A	DROSHA	P->S	83	1374	MISSENSE
22	37769489	G	A	ELFN2	H->Y	696	820	MISSENSE
3	27759216	C	T	EOMES	R->Q	469	686	MISSENSE
1	29344900	C	G	EPB41	P->R	357	864	MISSENSE
2	212570070	C	T	ERBB4	V->I	391	1308	MISSENSE
**5**	**14687661**	**G**	**C**	**FAM105B (OTULIN)**	**W->S**	**167**	**352**	**MISSENSE**
**5**	**14678818**	**G**	**A**	**FAM105B (OTULIN)**	**M->I**	**86**	**352**	**MISSENSE**
3	68055776	A	G	FAM19A1	M->V	3	133	MISSENSE
6	17601058	G	A	FAM8A1	A->T	140	413	MISSENSE
6	17601044	T	C	FAM8A1	L->P	135	413	MISSENSE
6	17601035	A	G	FAM8A1	H->R	132	413	MISSENSE
6	17601041	G	T	FAM8A1	G->I (COMBINED:17601040-17601041)	134	413	MISSENSE
6	17601040	G	A	FAM8A1	G->I (COMBINED:17601040-17601041)	134	413	MISSENSE
17	80045042	C	T	FASN	R->Q	1104	2511	MISSENSE
4	187629478	T	C	FAT1	I->V	502	4588	MISSENSE
12	133158672	C	T	FBRSL1	P->L	717	1045	MISSENSE
1	241661240	G	T	FH	T->K	474	510	MISSENSE
1	241661233	G	T	FH	H->K (COMBINED:241661233-241661235)	476	510	MISSENSE
1	241661235	G	T	FH	H->K (COMBINED:241661233-241661235)	476	510	MISSENSE
1	241661237	G	T	FH	A->K (COMBINED:241661237-241661238)	475	510	MISSENSE
1	241661238	C	T	FH	A->K (COMBINED:241661237-241661238)	475	510	MISSENSE
9	14776131	C	G	FREM1	A->P	1505	2179	MISSENSE
12	51757930	G	A	GALNT6	R->C	342	622	MISSENSE
17	61957914	C	T	GH2	S->N	225	256	MISSENSE
4	158074079	C	A	GLRB	Q->K	372	497	MISSENSE
15	23688956	C	T	GOLGA6L2	A->T	187	909	MISSENSE
19	48945183	G	A	GRIN2D	D->N	804	1336	MISSENSE
7	142961224	C	T	GSTK1	R->W	40	282	MISSENSE
1	89318970	A	G	GTF2B	Y->H	293	316	MISSENSE
15	63927063	C	T	HERC1	A->T	4147	4861	MISSENSE
4	89358115	C	T	HERC6	R->C	826	1022	MISSENSE
6	32714033	C	A	HLA-DQA2	A->T (COMBINED:32714031-32714033)	210	255	MISSENSE
6	32714031	G	A	HLA-DQA2	A->T (COMBINED:32714031-32714033)	210	255	MISSENSE
1	161496006	A	G	HSPA6	M->V	520	643	MISSENSE
6	87726029	C	A	HTR1E	A->D	326	365	MISSENSE
1	79101115	G	A	IFI44L	G->R	273	452	MISSENSE
9	34657470	G	T	IL11RA	A->S	178	422	MISSENSE
19	34832596	C	G	KIAA0355	T->R	586	1070	MISSENSE
7	151849934	T	G	KMT2C	S->R	4128	4911	MISSENSE
4	17583382	G	C	LAP3	N/A	N/A	N/A	SPLICE (1)
15	59500230	A	C	LDHAL6B,MYO1E	H->P (COMBINED:59500230-59500231)	364	381	MISSENSE
15	59500231	T	C	LDHAL6B,MYO1E	H->P (COMBINED:59500230-59500231)	364	381	MISSENSE
5	77805916	G	A	LHFPL2	R->C	41	228	MISSENSE
19	54803303	G	C	LILRA3	P->R	125	439	MISSENSE
7	156518154	T	C	LMBR1	K->R	378	490	MISSENSE
16	48290570	G	A	LONP2	R->H	173	852	MISSENSE
2	141264446	T	C	LRP1B	N->D	2814	4599	MISSENSE
7	150034027	G	A	LRRC61	G->D	26	259	MISSENSE
21	47630642	T	C	LSS	Q->R	385	732	MISSENSE

2	128096566	C	T	MAP3K2	R->Q	22	619	MISSENSE
6	83947435	C	A	ME1	N/A	N/A	N/A	SPLICE (1)
19	2078331	C	T	MOB3A	G->S	77	217	MISSENSE
12	62887808	G	T	MON2	E->Stop	97	1717	NONSENSE
17	10355577	C	A	MYH4	R->L	1140	1939	MISSENSE
22	26291213	C	A	MYO18B	S->Stop	1545	2567	NONSENSE
1	120484299	T	A	NOTCH2	D->V	944	2471	MISSENSE
11	71725341	G	A	NUMA1	R->C	1070	2115	MISSENSE
12	123463837	G	A	OGFOD2	G->S	273	290	MISSENSE
11	5758623	C	G	OR56B1	P->A	293	324	MISSENSE
2	26707421	C	T	OTOF	D->N	376	1997	MISSENSE
3	142567287	T	G	PCOLCE2	N->H	74	415	MISSENSE
1	156884577	G	A	PEAR1	R->H	1034	1037	MISSENSE
12	76425515	G	A	PHLDA1	R->C	3	401	MISSENSE
8	144995705	C	T	PLEC	E->K	2899	4684	MISSENSE
1	204219717	C	T	PLEKHA6	R->Q	517	1048	MISSENSE
8	96166355	C	T	PLEKHF2	P->L	28	249	MISSENSE
7	132192962	T	C	PLXNA4	N->S	164	1894	MISSENSE
6	43565474	C	G	POLH	L->V	178	713	MISSENSE
5	89781410	G	A	POLR3G	R->H	9	223	MISSENSE
1	150318520	A	G	PRPF3	K->R	556	683	MISSENSE
1	228033725	G	A	PRSS38	G->D	266	326	MISSENSE
4	152201098	A	G	PRSS48	H->R	68	328	MISSENSE
2	33774685	G	A	RASGRP3	S->N	470	690	MISSENSE
1	173951948	T	A	RC3H1	R->Stop	229	1133	NONSENSE
17	29325882	C	G	RNF135	H->Q	324	432	MISSENSE
1	35485145	C	A	RP11-244H3.4,ZMYM6	L->F	79	723	MISSENSE
1	114340587	T	C	RSBN1	K->E	259	802	MISSENSE
18	76754188	C	T	SALL3	P->S	733	1300	MISSENSE
20	50408175	C	T	SALL4	G->S	283	1053	MISSENSE
10	75528638	C	G	SEC24C	A->G	751	1094	MISSENSE
15	49309059	G	T	SECISBP2L	Q->K	469	1101	MISSENSE
15	49309062	C	T	SECISBP2L	E->K (COMBINED:49309060-49309062)	468	1101	MISSENSE
15	49309060	C	T	SECISBP2L	E->K (COMBINED:49309060-49309062)	468	1101	MISSENSE
15	49309064	A	T	SECISBP2L	M->K (COMBINED:49309063-49309064)	467	1101	MISSENSE
15	49309063	C	T	SECISBP2L	M->K (COMBINED:49309063-49309064)	467	1101	MISSENSE
10	120919247	G	A	SFXN4	A->V (COMBINED:120919246-120919247)	118	337	MISSENSE
4	48487028	A	T	SLC10A4	I->F	224	437	MISSENSE
5	127484447	C	T	SLC12A2	A->V	628	1212	MISSENSE
21	46945816	T	C	SLC19A1	N->S	403	591	MISSENSE
11	66136991	G	A	SLC29A2	R->Stop	42	456	NONSENSE
17	1479056	T	C	SLC43A2	N->D	518	569	MISSENSE
17	18219863	C	T	SMCR8	R->C	254	937	MISSENSE
17	1690205	A	G	SMYD4	F->L	595	804	MISSENSE
21	34924222	G	C	SON	M->I	895	2426	MISSENSE
2	54895611	G	A	SPTBN1	V->I	2334	2364	MISSENSE
11	66472100	G	A	SPTBN2	R->C	883	2390	MISSENSE
11	66807490	C	T	SYT12	T->I	146	421	MISSENSE
6	159185613	G	C	SYTL3	D->H	604	610	MISSENSE
14	104460689	A	C	TDRD9	M->L	401	1382	MISSENSE
12	53452596	G	A	TENC1	E->K	474	1419	MISSENSE
15	90169242	C	T	TICRR	P->L	1851	1910	MISSENSE
7	47332467	G	A	TNS3	T->M	1270	1445	MISSENSE
1	36603504	T	C	TRAPPC3	I->V	106	180	MISSENSE
7	100465536	T	C	TRIP6	Y->H	55	476	MISSENSE
15	31323186	G	A	TRPM1	L->F	1060	1642	MISSENSE
2	179414833	C	T	TTN	V->I	30578	35991	MISSENSE
2	170735046	G	A	UBR3	A->T	334	1888	MISSENSE
11	11971915	C	T	USP47	R->C	1089	1287	MISSENSE
1	160389131	G	A	VANGL2	V->I	178	521	MISSENSE
15	101817623	G	A	VIMP	N/A	N/A	N/A	SPLICE (1)
11	61048662	C	T	VWCE	R->Q	278	955	MISSENSE
16	78458893	G	C	WWOX	Q->H	244	414	MISSENSE
5	32385662	T	C	ZFR	I->V	865	1074	MISSENSE
17	30693723	A	G	ZNF207	N->D	290	494	MISSENSE
19	57086006	G	C	ZNF470	N/A	N/A	N/A	SPLICE (1)
19	56903115	G	C	ZNF582	L->V	3	517	MISSENSE
19	37382719	T	G	ZNF829	E->A	406	513	MISSENSE

Appendix Table S6. Homozygous small insertions and deletions
This table summarizes all homozygous small insertions and deletions (InDel) identified in the patient by whole exome sequencing (WES).

Chromosome	Position start of variant	Position end of variant	Type of variant	Length of variant	Gene	Localization of variant
**11**	3661585	3661585	INS	3	ART5	EXON
**1**	54605319	54605319	INS	1	CDCP2	EXON
**2**	211421452	211421452	INS	3	CPS1	EXON
**7**	27239320	27239337	DEL	18	HOXA13	EXON
**19**	50003817	50003817	INS	2	hsa-mir-150	EXON
**19**	35758275	35758275	INS	3	LSR	EXON
**17**	48227384	48227384	INS	2	PPP1R9B	SPLICE (1)
**14**	24646406	24646406	INS	3	REC8	EXON
**17**	26699199	26699199	INS	1	SARM1	EXON
**X**	101395780	101395780	INS	1	TCEAL6	EXON

Appendix Table S7. Heterozygous small insertions and deletions
This table summarizes all heterozygous small insertions and deletions (InDel) identified in the patient by whole exome sequencing (WES).

Chromosome	Position start of variant	Position end of variant	Type of variant	Length of variant	Gene	Localization of variant
**14**	74060511	74060511	INS	4	ACOT4	EXON
**6**	151674116	151674116	INS	3	AKAP12	EXON
**20**	47649621	47649621	DEL	1	ARFGEF2	EXON
**11**	3661585	3661585	INS	3	ART5	EXON
**11**	4592708	4592708	INS	2	C11orf40	EXON
**11**	66512290	66512290	INS	3	C11orf80	EXON
**9**	116187645	116187645	INS	3	C9orf43	EXON
**17**	77807917	77807917	INS	6	CBX4	EXON
**3**	56650051	56650051	INS	3	CCDC66	EXON
**12**	51723598	51723598	INS	1	CELA1	EXON
**9**	95237025	95237027	DEL	3	CENPP,ASPN	EXON
**5**	179287622	179287622	INS	2	CTC-241N9.1	EXON
**12**	2062323	2062323	INS	3	DCP1B	EXON
**2**	29258439	29258439	INS	5	FAM179A	EXON
**6**	1612016	1612016	INS	6	FOXC1	EXON
**1**	47904668	47904668	INS	6	FOXD2	EXON
**16**	12009530	12009530	INS	3	GSPT1	EXON
**19**	1085845	1085845	INS	6	HMHA1	EXON
**19**	49657710	49657710	INS	3	HRC	EXON
**19**	49657889	49657889	INS	3	HRC	EXON
**9**	21228002	21228002	INS	1	IFNA17	EXON
**17**	39622430	39622430	INS	2	KRT32	SPLICE (2)
**7**	107735724	107735727	DEL	4	LAMB4	EXON
**8**	98788165	98788165	INS	16	LAPTM4B	EXON
**1**	152681680	152681680	INS	18	LCE4A	EXON
**19**	35758275	35758275	INS	3	LSR	EXON
**9**	12775861	12775861	INS	9	LURAP1L	EXON
**3**	65425560	65425560	INS	3	MAGI1	EXON
**1**	1684347	1684347	INS	3	NADK	EXON
**3**	101576029	101576029	INS	23	NFKBIZ	SPLICE (2)
**15**	23006299	23006299	INS	3	NIPA2	EXON
**5**	175811094	175811094	INS	2	NOP16	EXON
**14**	24769849	24769849	INS	6	NOP9	EXON
**20**	334235	334235	DEL	1	NRSN2	EXON
**19**	15052983	15052983	INS	3	OR7C2	EXON
**2**	178494173	178494173	INS	3	PDE11A	EXON
**13**	73409508	73409508	INS	1	PIBF1	SPLICE (2)
**10**	118215310	118215310	INS	1	PNLIPRP3	EXON
**6**	99283172	99283172	INS	3	POU3F2	EXON
**1**	14106394	14106394	INS	3	PRDM2	EXON
**20**	32664864	32664864	INS	3	RALY	EXON
**14**	23371255	23371255	INS	3	RBM23	EXON
**3**	40503520	40503520	INS	9	RPL14	EXON
**3**	40503520	40503520	INS	6	RPL14	EXON
**1**	31905889	31905889	INS	3	SERINC2	EXON
**21**	40883671	40883671	INS	3	SH3BGR	EXON
**5**	127419932	127419940	DEL	9	SLC12A2	EXON
**21**	34003928	34003928	INS	6	SYNJ1	EXON
**X**	101395780	101395780	INS	1	TCEAL6	EXON
**2**	120194651	120194651	INS	6	TMEM37	EXON
**9**	73458045	73458045	INS	1	TRPM3	SPLICE (2)
**1**	151518313	151518313	INS	4	TUFT1	EXON
**12**	122359397	122359397	INS	15	WDR66	EXON
**X**	119387833	119387833	INS	3	ZBTB33	EXON
**16**	31770696	31770696	INS	1	ZNF720	EXON

Appendix Table S8. Obesity Gene Sets

This table provides a list of obesity genes from a genome-wide association study (GWAS) (Rouillard *et al*, 2016) and a list of genes known to be associated with childhood obesity (Chesi & Grant, 2015; Warner *et al*, 2021).

**Obesity Gene Set (Rouillard *et al.*)**				**Childhood Obesity Gene Set (Warner *et al.*; Chesi & Grant)**	
TFAP2B	MDFIC	LHFPL3	ANO3	FTO	RMST
C8ORF37	KCNMA1	C9ORF84	KCTD8	ADCY3	TNKS-MSRA
PCDH9	FAT1	VEGFA	MAP1A	TMEM18B	LEPR
THEMIS	CDH12	DNM3	TBX15	SEC16B	PRKCH
GDAP1	HOXB5	PIGC	APOC1	TNNI3K	PACS1
NISCH	COL21A1	POC5	KCTD15	MC4R	HOXB5
EML6	HOXB3	RARB	SPRY2	TFAP2B	OLFM4
SFRP2	FBN2	EXOC4	LCT	GPR61	ABCB5
OLFM4	INO80D	EIF4E3	CAMK1G	AL513166.1	ARL15
MEX3B	FTO	NFE2L3	PFKP	SLC39A8	EDIL3
GNPDA2	LRRFIP1	NRXN1	TRUB2	PRKD1	EPHA6-UNQ6114
RPS17P5	IFIT1	ITPR2	MTCH2	FAM110A	FOXP2
CPEB4	LIN7C	ITPR3	HDAC4	FHIT	KIF2B
ALPK1	SLC30A10	NRXN3	DIRAS2	BDNF	OR4P4-OR4S2-OR4C6
PKP1	LIPA	HOXC13	FAIM2	FAIM2	S1PR5
HCN4	RGS6	CHD2	ATP2A1	QPCTL	
HOXB1	CYB5R2	TTC28	ZNRF3	GNPDA2	
ASAH1	PKHD1	MLN	MC4R	INSIG2	
SV2C	CMYA5	STK33	RSPO3	KCTD15	
MACROD2	COBLL1	NCAM2	ENPP2	NEGR1	
SH2B1	IFITM8P	FAM83B	ADAMTS9	NRXN3	
ST3GAL1	ZNF248	WWOX	MRPS22	POMC-ADCY3	
ANKDD1B	SOX6	PALD1	RFTN1	HHEX-IDE	
TBCE	DARS	ARG1	NPC1	SDCCAG8	
INHBB	KDM4C	OVCH2		SH2B1	
NAALADL2	BCDIN3D	NEGR1		TBCA	
ARHGAP12	PAX5	MSRA		ZPLD1	

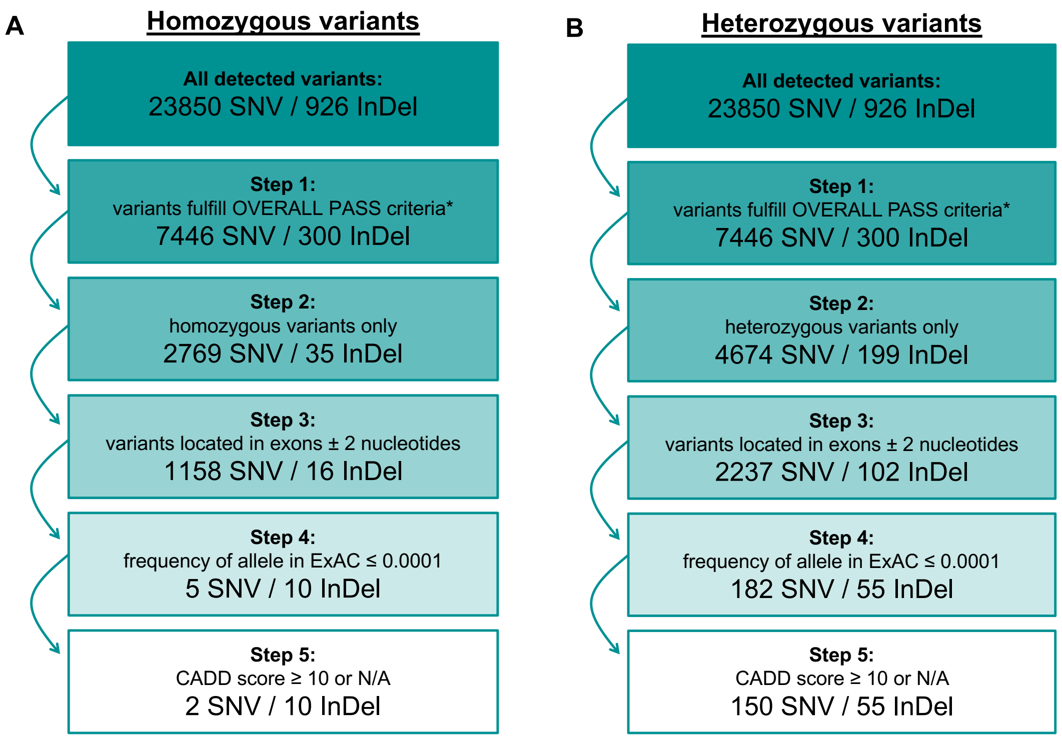

Appendix Figure S1. Filtering strategy WES

The filtering strategy for identification of homozygous and heterozygous variants in the patient is depicted. *OVERALL PASS criteria: read depth ≥ 5, quality score reads ≥ 20, frequency dbSNP ≤ 0.02, exons ± 10 nucleotides.

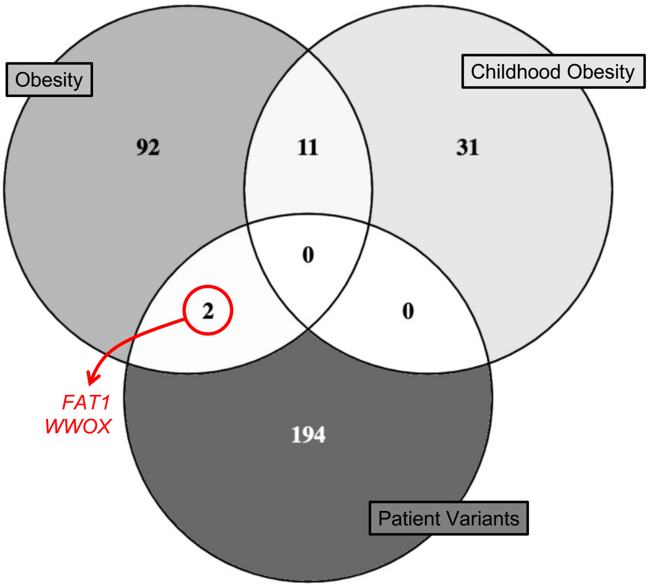

Appendix Figure S2. Venn's diagram comparing patient variants with genes known to be involved in obesity and childhood obesity, respectively.
Single nucleotide variants (SNVs) and small insertions and deletions (InDel) identified by whole exome sequencing (WES) in the patient (n=196) were compared with the obesity gene set (Rouillard *et al.*, 2016) (n=105) and a list of genes known to be associated with childhood obesity (Chesi & Grant, 2015; Warner *et al.*, 2021) (n=42) using Venny (Oliveros, J.C. (2007-2015) Venny. An interactive tool for comparing lists with Venn's diagrams.).

References

Chesi A, Grant SFA (2015) The Genetics of Pediatric Obesity. *Trends Endocrinol Metab* 26: 711-721

Rouillard AD, Gundersen GW, Fernandez NF, Wang Z, Monteiro CD, McDermott MG, Ma'ayan A (2016) The harmonizome: a collection of processed datasets gathered to serve and mine knowledge about genes and proteins. *Database (Oxford)* 2016

Warner ET, Jiang L, Adjei DN, Turman C, Gordon W, Wang L, Tamimi R, Kraft P, Lindstrom S (2021) A Genome-Wide Association Study of Childhood Body Fatness. *Obesity (Silver Spring)* 29: 446-453
