# Supplementary material for: Compound heterozygous variants in OTULIN are associated with fulminant atypical late‐onset ORAS
Source: EMBO Mol Med. 2022 Feb 16;14(3):e14901. doi: 10.15252/emmm.202114901 (PMC8899767; doi:10.15252/emmm.202114901)
Supplement: Supplementary file 2 — Expanded View Figures PDF [file EMMM-14-e14901-s005.pdf]

Expanded View Figures

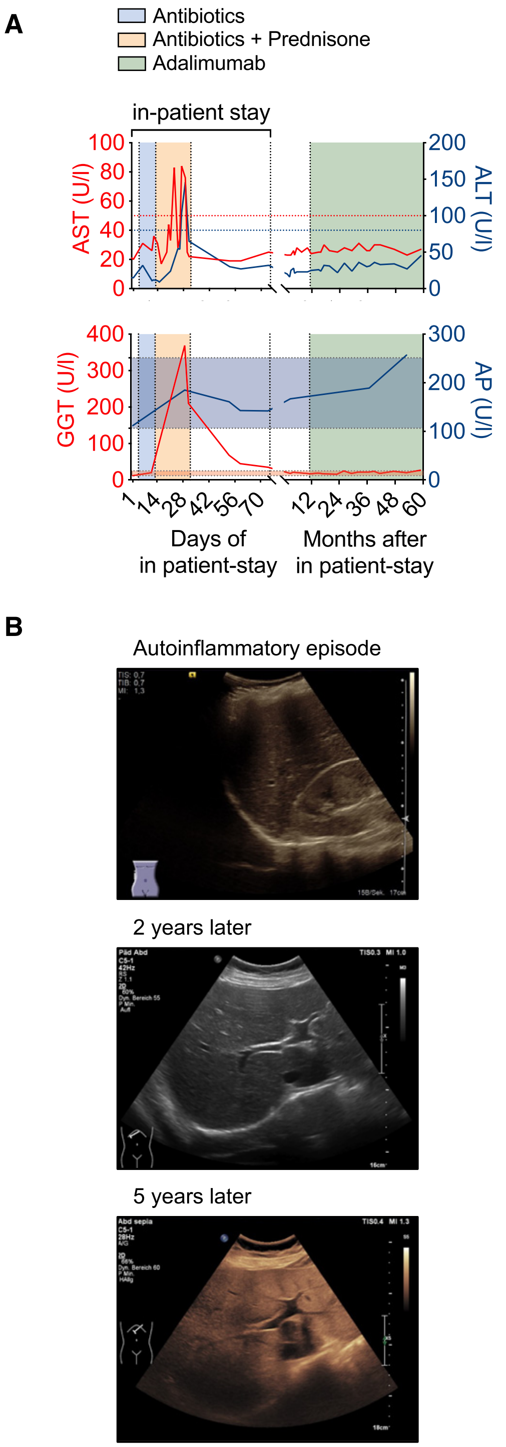

Figure EV1. Abnormal liver function test and development of steatosis hepatis grade II.

A The course of the patient's liver enzymes AST (aspartate transaminase) and ALT (alanine transaminase) (upper panel) or GGT (γ-glutamyltransferase) and AP (alkaline phosphatase) (lower panel) are depicted along the time axis with the upper limit of normal indicated by red and blue lines for AST and ALT and the normal range in shades of red and blue for GGT and AP, respectively.

B Liver ultrasound B-mode images of the patient during the initial phase of autoinflammation and about 2 and 5 years thereafter display diffuse increase of liver echogenicity and finally slightly impaired appearance of portal vein wall and diaphragm indicative of steatosis hepatis grade II.

© 2022 The Authors

Figure EV2. Diminished OTULIN protein expression in patient-derived cells is not due to reduced mRNA expression or increased degradation *via* the proteasome or the lysosome.

A OTULIN protein is depicted.

B OTULIN antibodies by Abcam (antigen corresponding to AA 60–158) or by Cell Signaling (recombinant fragment surrounds S76 without spanning M86) were tested in parallel on A549 *OTULIN* KO cells transfected with different OTULIN constructs as indicated. One representative of two independent experiments is shown.

C Relative mRNA expression of *OTULIN* in fibroblasts and B cells with two different primer pairs is depicted. Data are presented as mean ± SD of six independent experiments; dots represent individual experiments performed in three technical replicates; ns, non-significant, unpaired *t*-test.

D A549 *OTULIN* KO cells were transfected with the different OTULIN constructs as indicated. The following day, cells were treated with 50 μg/ml cycloheximide (CHX) for the indicated times, harvested and analyzed by Western blot for OTULIN protein expression. Tubulin served as loading control. One representative of three independent experiments is shown.

E A549 *OTULIN* KO cells were transfected with the different OTULIN constructs as indicated. The following day, cells were treated with 50 μg/ml CHX alone or in combination with 1 μM Bortezomib (BTZ) or 1 μM Bafilomycin A1 (Baf A1) for 8 h or left untreated (DMSO). Expression of the proteins indicated was analyzed by Western blot. One representative of three independent experiments is shown.

© 2022 The Authors

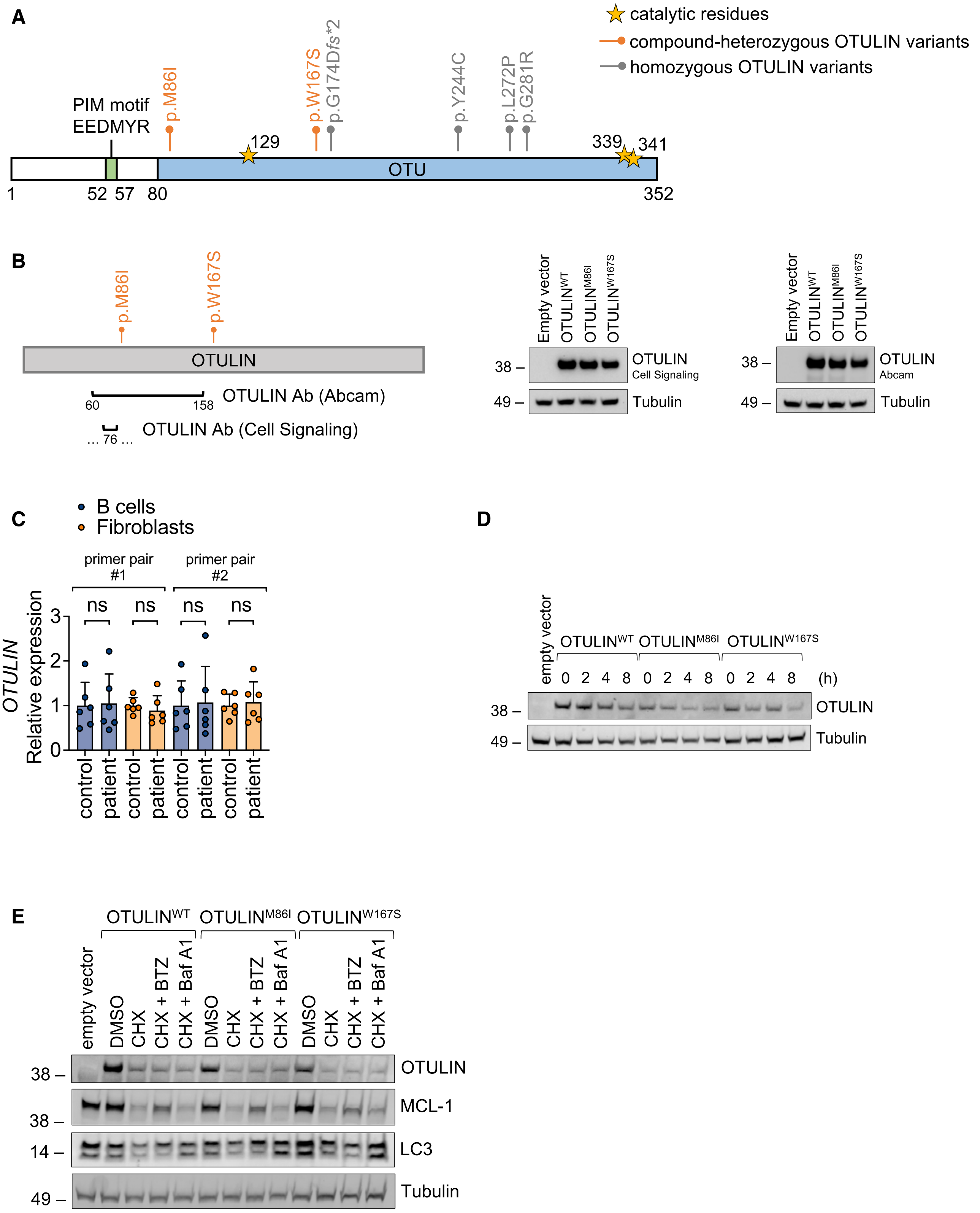
A
catalytic residues
compound-heterozygous OTULIN variants
homozygous OTULIN variants
PIM motif
EEDMYR
p.M86I
p.W167S
p.G174Dfs*2
p.Y244C
p.L272P
p.G281R
129
339
341
OTU
1
52
57
80
352
B
p.M86I
p.W167S
OTULIN
60
158
OTULIN Ab (Abcam)
... 76 ...
OTULIN Ab (Cell Signaling)
Empty vector
OTULINWT
OTULINM86I
OTULINW167S
38 –
49 –
OTULIN
Cell Signaling
Tubulin
OTULIN
Abcam
C
B cells
Fibroblasts
primer pair #1
primer pair #2
ns
OTULIN Relative expression
control
patient
D
empty vector
OTULINWT OTULINM86I OTULINW167S
0 2 4 8 0 2 4 8 0 2 4 8 (h)
OTULIN
Tubulin
E
OTULINWT OTULINM86I OTULINW167S
empty vector
DMSO
CHX
CHX + BTZ
CHX + Baf A1
OTULIN
MCL-1
LC3
Tubulin
14 –

Figure EV2.

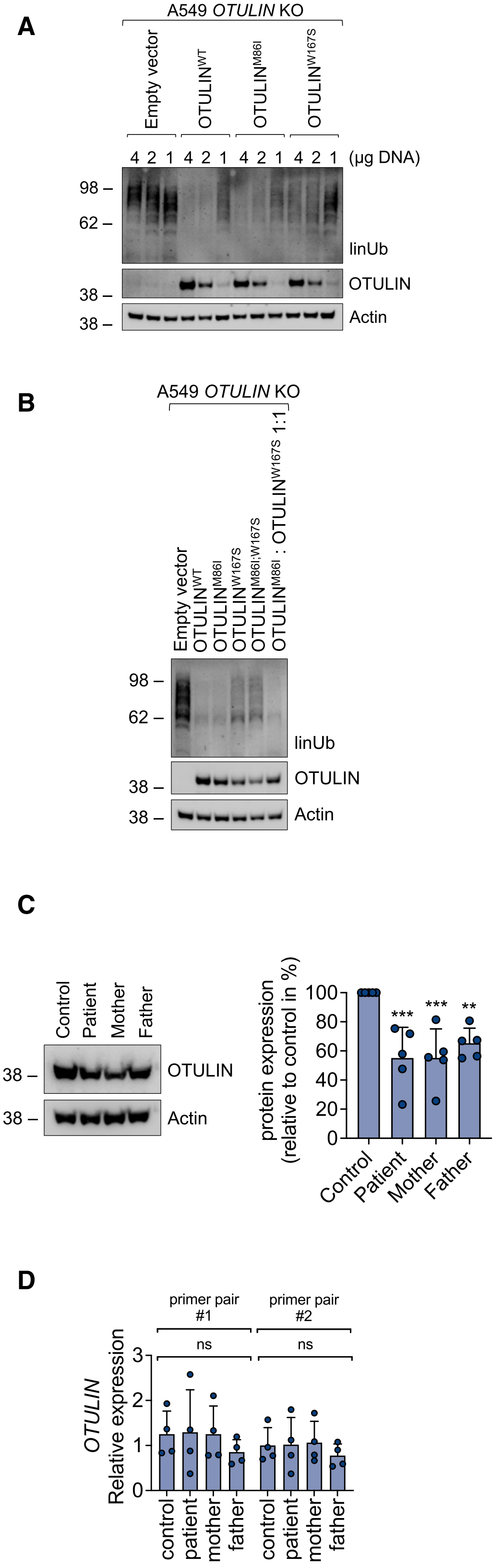

Figure EV3. The maternal OTULIN variant p.W167S more severely impairs hydrolysis of linear ubiquitin linkages than the paternal OTULIN variant p.M86I.

A, B A549 *OTULIN* KO cells were transfected with different OTULIN constructs as indicated. After 24 h, cells were lysed and subjected to analysis by Western blot. One representative of three independent experiments is shown.

C OTULIN protein expression in B cells derived from control, patient, mother, and father is shown. Blots (left panel) are representative of five independent experiments analyzed by densitometry (right panel). Data are presented as mean ± SD (dots represent individual experiments); ****P* = 0.0008, ***P* = 0.0069, ordinary one-way ANOVA, Dunnett's multiple comparisons test.

D Relative mRNA expression of *OTULIN* in B cells derived from control, patient, mother, and father determined with two different primer pairs is depicted. Data are presented as mean ± SD of four independent experiments; dots represent individual experiments performed in three technical replicates; ns, non-significant, ordinary one-way ANOVA, Dunnett's multiple comparisons test.

 © 2022 The Authors

Figure EV4. Signaling output of different innate immune stimuli in patient-derived cells.

A Fibroblasts were stimulated with 1 μg/ml LPS for the indicated times and subjected to analysis by Western blot. One representative out of three independent experiments is shown.

B Fibroblasts were stimulated with LPS for 24 h. Concentration of IL-6 in the supernatant was determined by ELISA. Data are presented as mean ± SD of three individual experiments performed in two technical replicates; **P* = 0.012; multiple *t*-tests corrected for multiple comparisons using the Holm–Sidak method.

C B cells were treated with 100 ng/ml CD40L for the indicated times and subjected to analysis by Western blot. One representative out of three independent experiments is shown.

D Fibroblasts were incubated with 50 μg/ml cycloheximide (CHX) for the indicated times and subjected to analysis by western blot. One representative (left panel) out of 4 individual experiments analyzed by densitometry (right panel) is shown. Data are presented as mean ± SD. **P* = 0.02, unpaired *t*-test.

E, F Fibroblasts (E) or B cells (F) were treated with 0.1 μg/ml TNF, 0.1 μg/ml TRAIL, 100 μg/ml poly(I:C), 1 μg/ml LPS or left untreated, and viability was determined after 24 h. Data are presented as mean ± SD of three independent experiments; dots represent individual experiments performed in three technical replicates; ns, non-significant, unpaired *t*-test.

G, H Fibroblasts were incubated with 50 μg/ml cycloheximide (CHX) or DMSO and stimulated with the indicated concentrations of TRAIL (G) or poly(I:C) (H). Viability was measured after 24 h. Data are presented as mean ± SD of three individual experiments performed in three technical replicates.

Source data are available online for this figure.

© 2022 The Authors

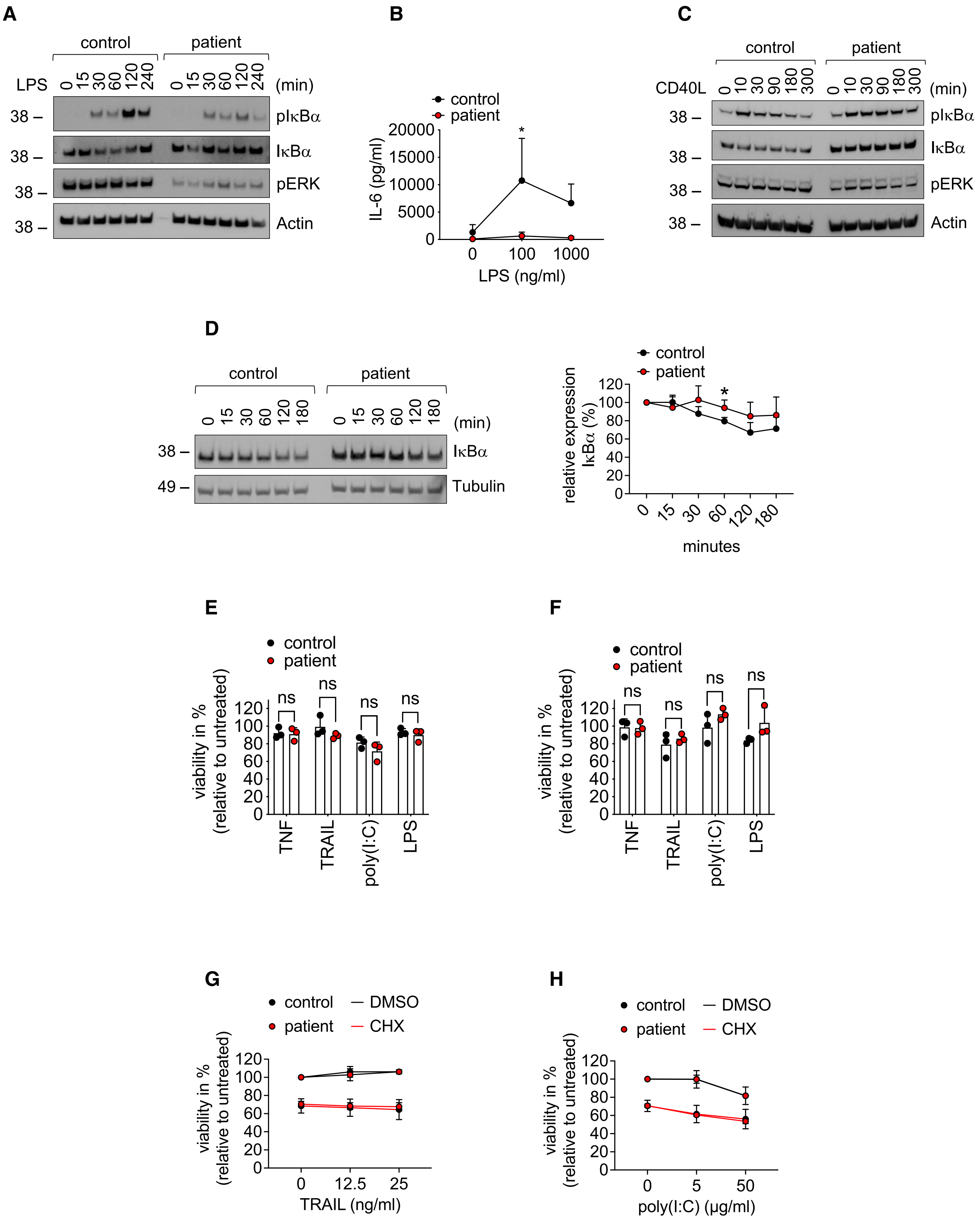

Figure EV4.

 © 2022 The Authors

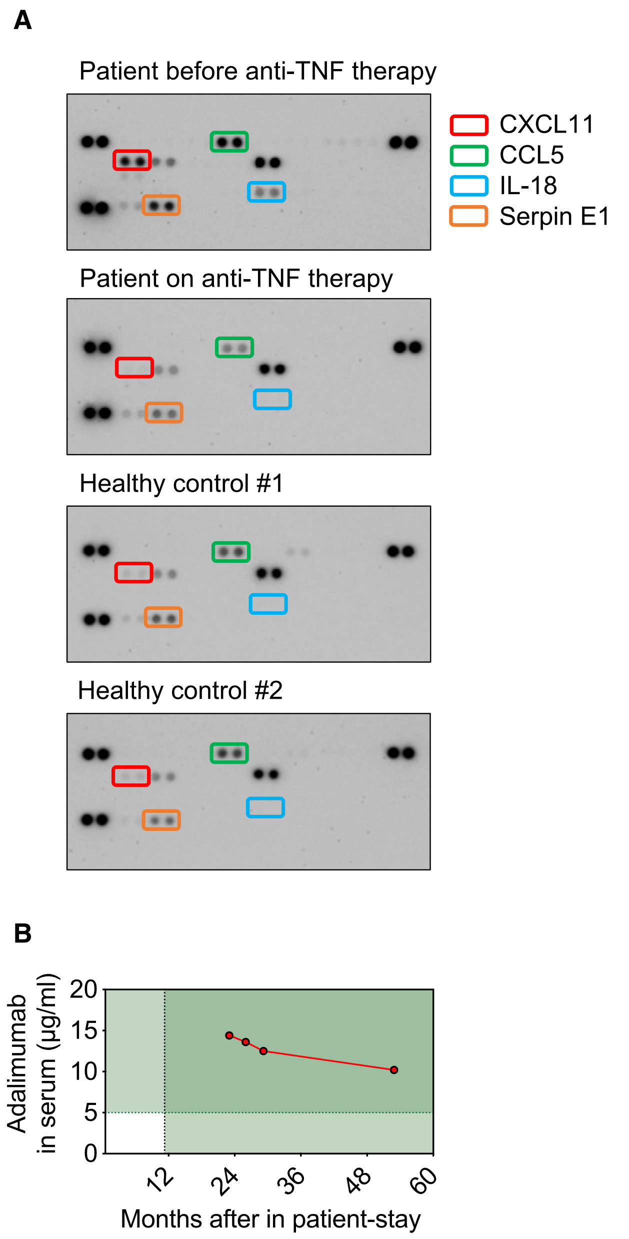

Figure EV5. The patient's inflammatory plasma protein profile is converted to normal after treatment with the anti-TNF therapeutic Adalimumab.

A Plasma was collected from the patient before and after start of therapy with the anti-TNF therapeutic Adalimumab. Plasma from two healthy controls was used as comparison. The Proteome Profiler Human Cytokine Array was performed according to the manufacturer's instructions.

B The concentration of Adalimumab in the patient's serum is shown (target concentration: > 5 µg/ml).

© 2022 The Authors
