# Supplementary material for: Compound heterozygous variants in OTULIN are associated with fulminant atypical late‐onset ORAS
Source: EMBO Mol Med. 2022 Feb 16;14(3):e14901. doi: 10.15252/emmm.202114901 (PMC8899767; doi:10.15252/emmm.202114901)
Supplement: Supplementary file 3 — Table EV1 [file EMMM-14-e14901-s003.docx]

**Table EV1. Overview of patients diagnosed with ORAS**

This table summarizes all patients with *OTULIN* variants described in the literature to date and compares them to the patient identified in this study.

|  | Present study | Damgaard  *et al.*, 2016 | Zhou  *et al.*, 2016 | Damgaard  *et al.*, 2019 | Nabavi  *et al.*, 2019 | Damgaard  *et al.*, 2020 |
| --- | --- | --- | --- | --- | --- | --- |
| cDNA alteration | pat.: c.258G>A  mat.: c.500G>C | a) c.815T>C  b) c.815T>C  c) c.815T>C | d) c.815T>C  e) c.731A>G  f) c.517delC | c.841G>A | c.864+2T>C | c.815T>C  (= patient a) |
| Type of mutation | Compound heterozygous, missense | Homozygous, missense | Homozygous, missense (d, e), premature stop (f) | Homozygous, missense | Homozygous, intronic | Homozygous, missense |
| Gestational age (premature (+)) | 38 weeks | a) + (34 weeks)  b) + (36weeks)  c) + (28 weeks) | + (1/3) | + (36 weeks) | + (35 weeks) | + (34 weeks) |
| Disease onset | 7 years | a) 3 weeks  b) 3 days  c) 8 weeks | d) Soon after birth  e) 4.5 months  f) Neonatal-onset | Shortly after birth | Soon after birth | 3 weeks |
| Age at death | alive | a) 16 months  b) 4 years  c) alive | alive | alive | 8.5 months | 16 months |
| Fever | + | + (3/3) | + (3/3) | + | + | + |
| Panniculitis | + | + (3/3) | + (3/3) | + | Erythematous nodules, no histology | + |
| Failure to thrive | - (obese) | + (3/3) | + (2/3) | + | N/A | + |
| Diarrhea | - | + (3/3) | + (1/3) | + | N/A | + |
| Arthralgia/  Arthritis | - | + (2/3) | + (2/3) | + | N/A | - |
| CRP ↑ | + | + (3/3) | + (3/3) | + | + | + |
| Leukocytosis | + | + (3/3) | + (3/3) | + | + | + |
| Neutrophilia | + | + (3/3) | N/A | - | + | + |
| Sterile abscess formation | + | N/A | N/A | N/A | N/A | N/A |
| Steatosis hepatis | + | N/A | N/A | N/A | N/A | + |
| Therapy  Efficient (+)  Inefficient (-) | Steroids (+)  Adalimumab (+) | Steroids (+)  AZA (-)  Anakinra (-)  MTX (-)  Infliximab (+) | Steroids (+)  Infliximab (+)  Etanercept (+)  Anakinra (+/-) | Prednisone (+)  colchicine (+)  Anakinra (-)  HSCT (+)  Etanercept (+) | Antibiotics, IFNγ (-) | Steroids (+)  AZA (-)  MTX (-) |
