# Supplementary figures and images for: Compound heterozygous variants in OTULIN are associated with fulminant atypical late‐onset ORAS
Source: EMBO Mol Med. 2022 Feb 16;14(3):e14901. doi: 10.15252/emmm.202114901 (PMC8899767; doi:10.15252/emmm.202114901)

Fig 2A

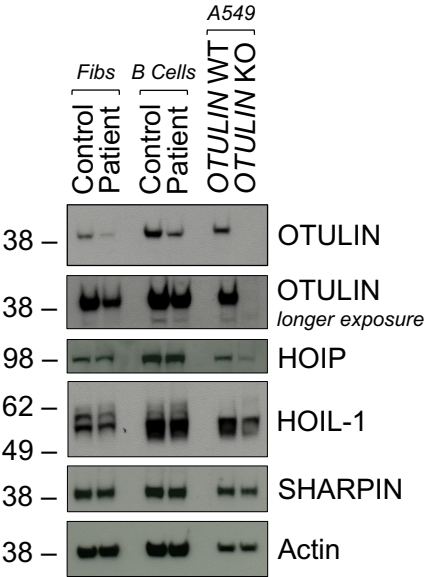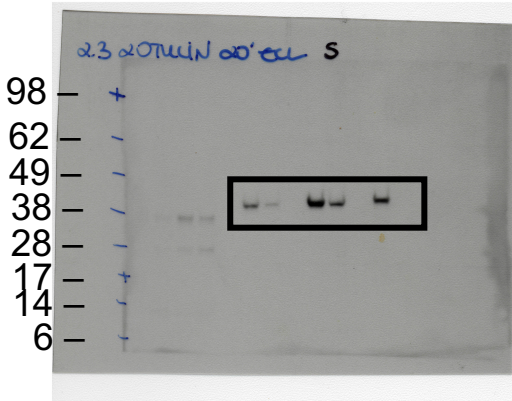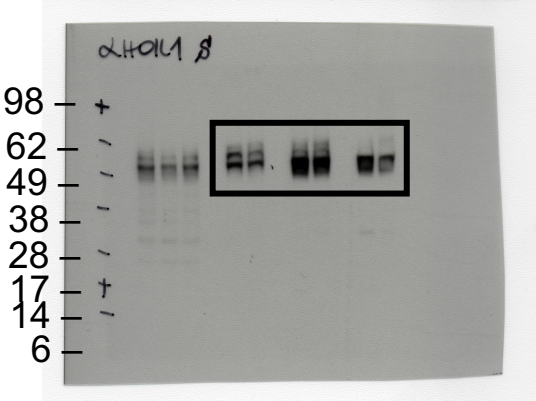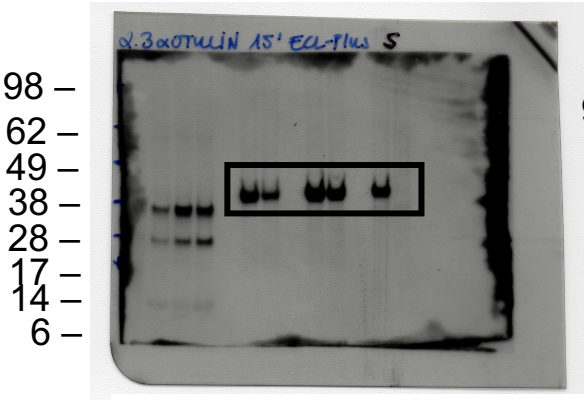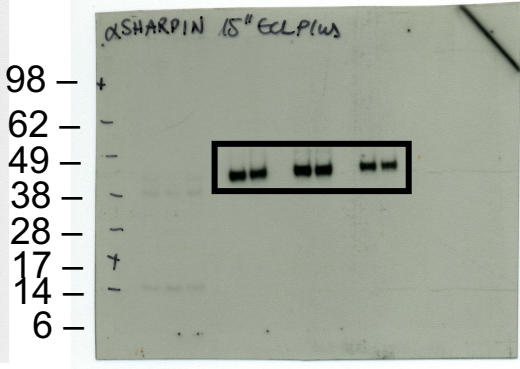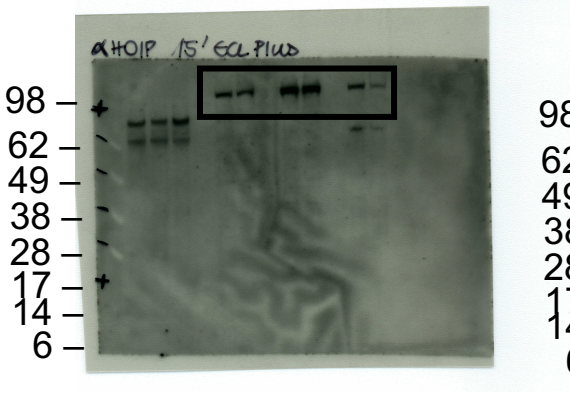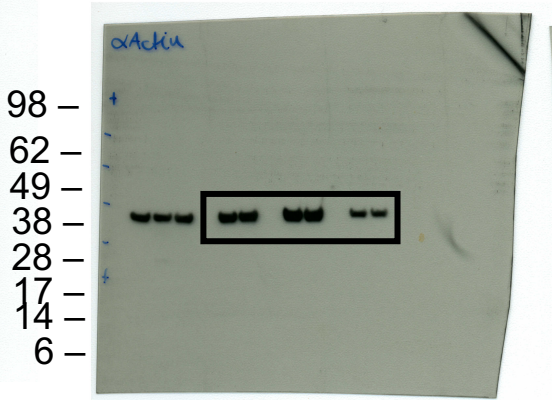

Supplement: Supplementary file 5 — Source Data for Figure 2 [file EMMM-14-e14901-s008.zip › EMM-2021-14901-V3-Figure_2A_Source_Data-sd.pdf]

Fig 2B

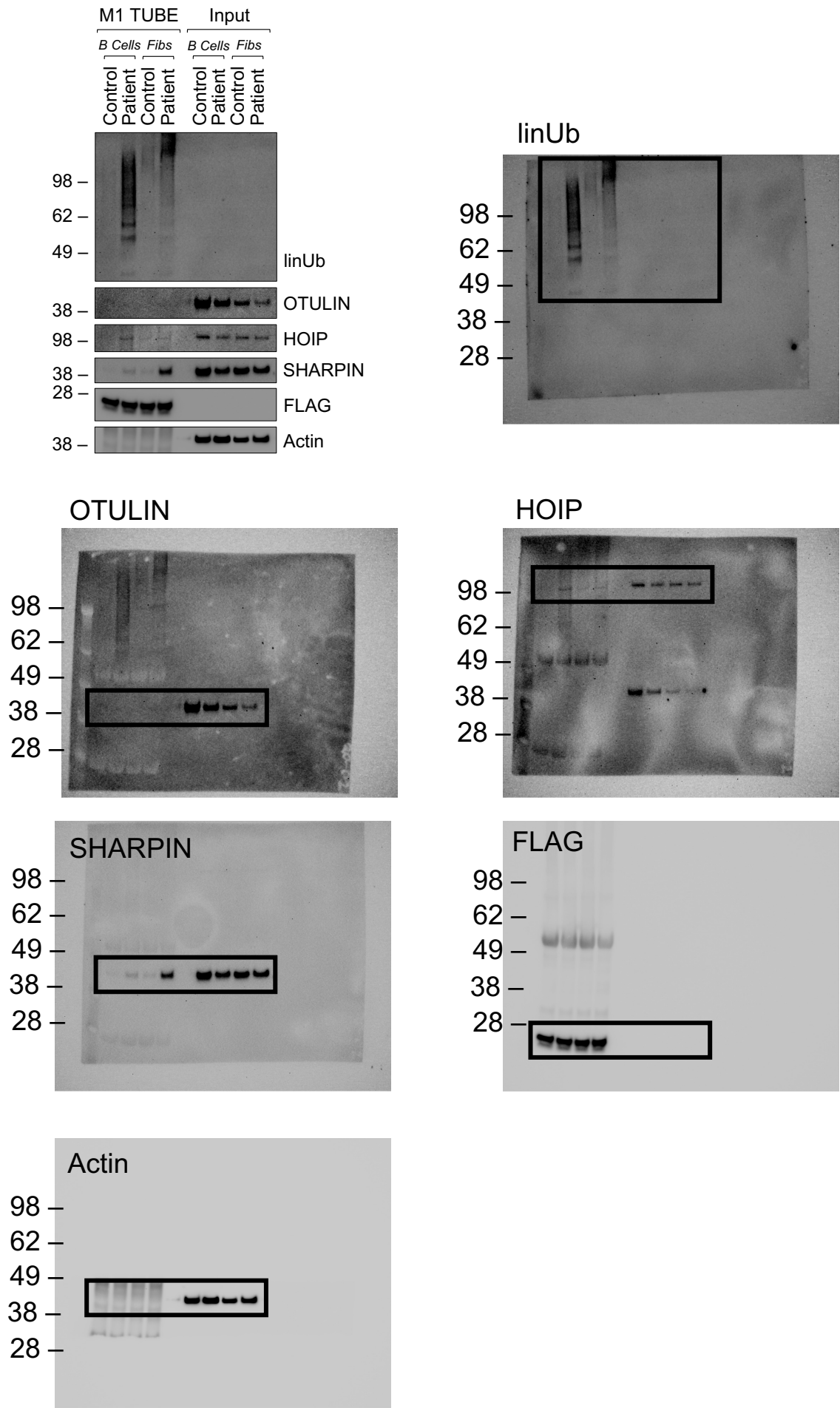

Supplement: Supplementary file 5 — Source Data for Figure 2 [file EMMM-14-e14901-s008.zip › EMM-2021-14901-V3-Figure_2B_Source_Data-sd.pdf]

Fig 5A

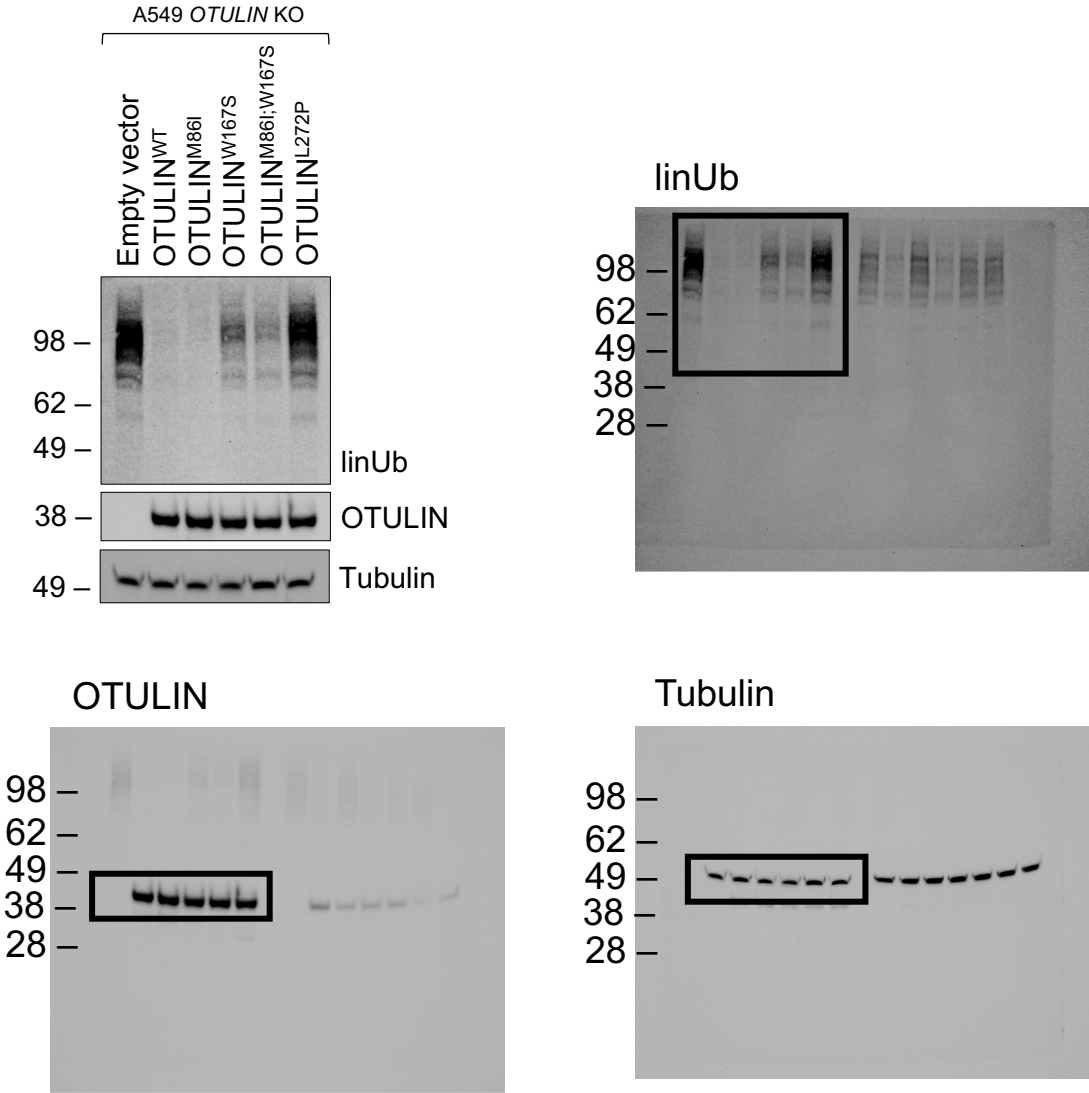

Supplement: Supplementary file 6 — Source Data for Figure 5 [file EMMM-14-e14901-s004.zip › EMM-2021-14901-V3-Figure_5A_Source_Data-sd.pdf]

Fig 5B

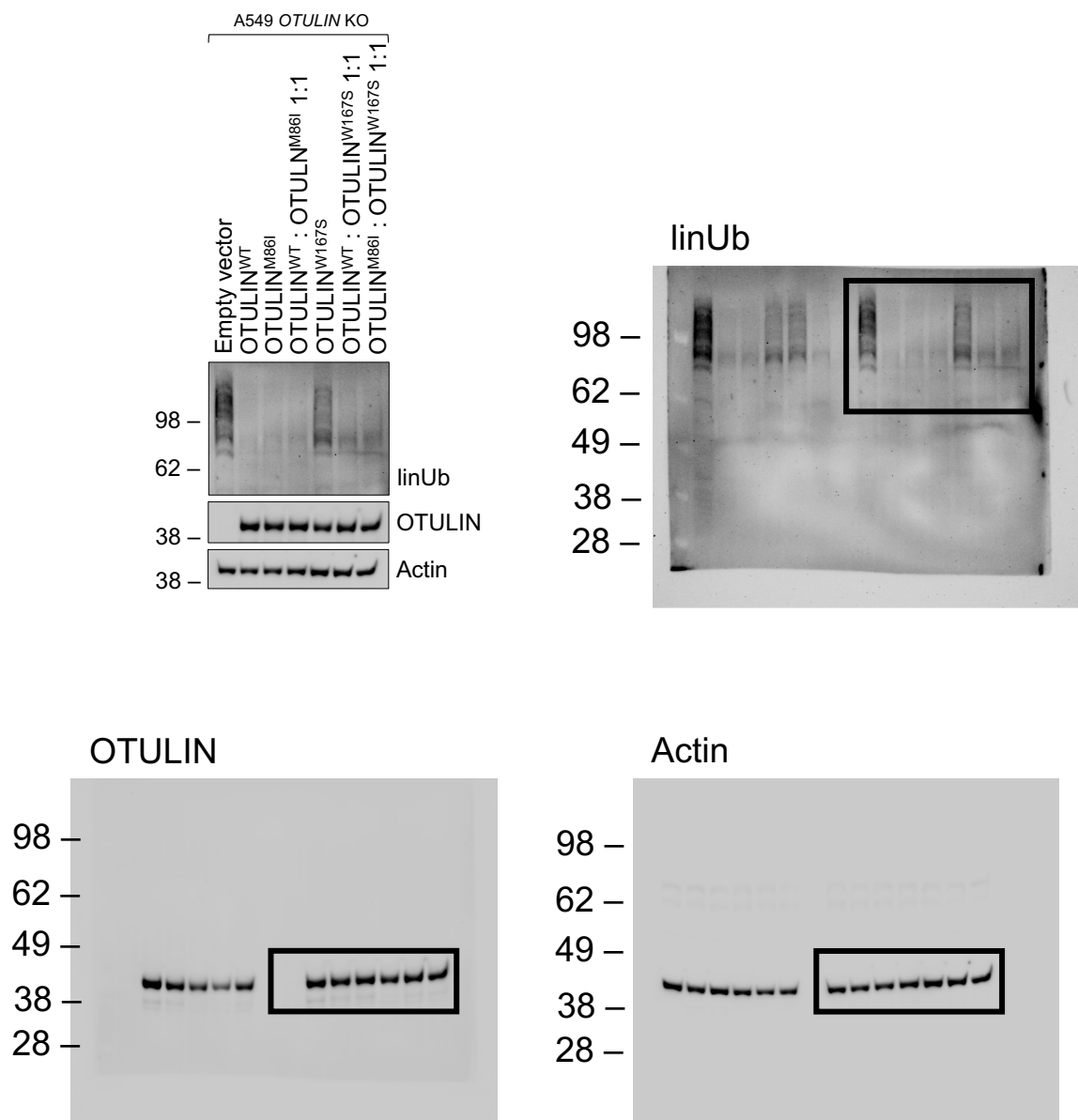

Supplement: Supplementary file 6 — Source Data for Figure 5 [file EMMM-14-e14901-s004.zip › EMM-2021-14901-V3-Figure_5B_Source_Data-sd.pdf]

Fig 5C

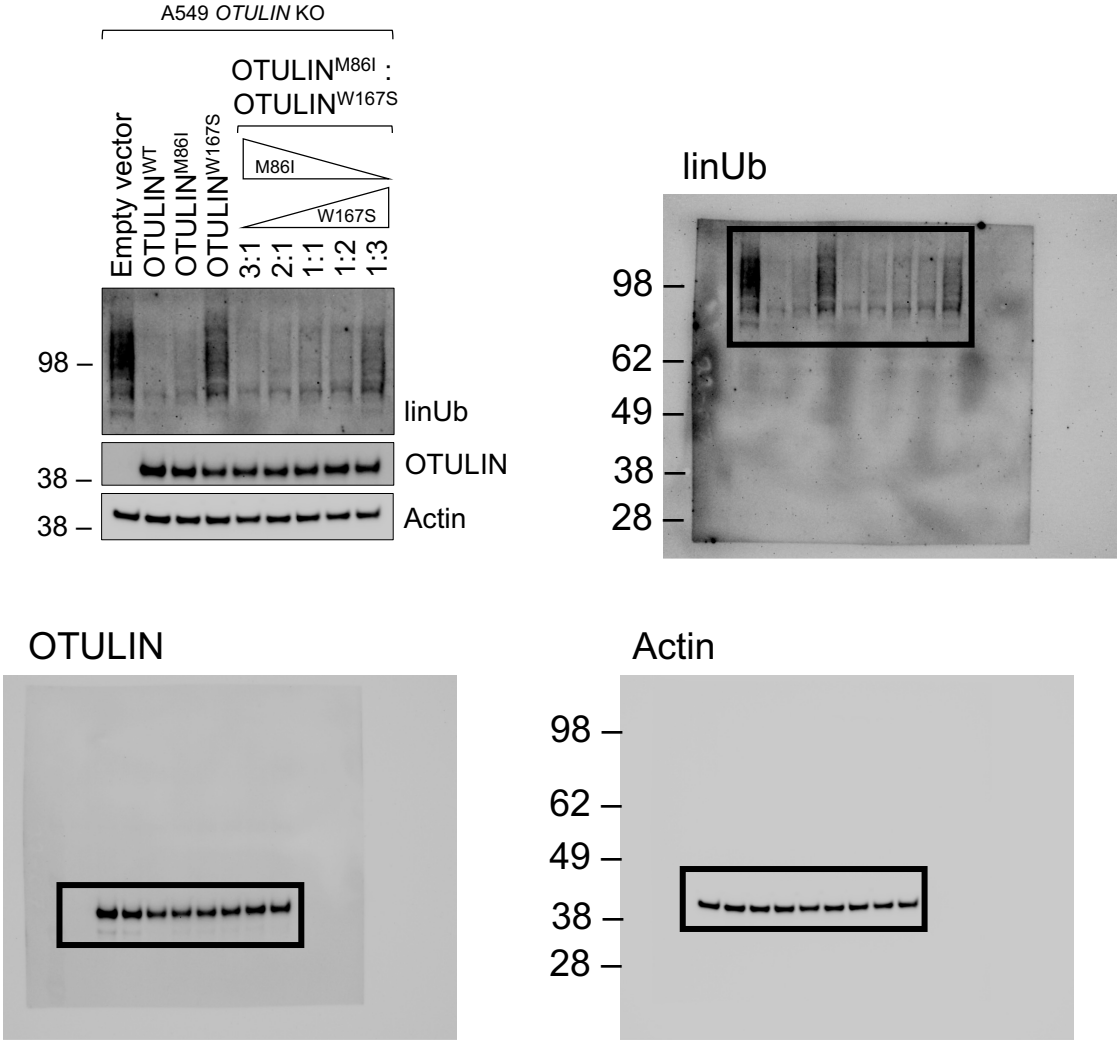

Supplement: Supplementary file 6 — Source Data for Figure 5 [file EMMM-14-e14901-s004.zip › EMM-2021-14901-V3-Figure_5C_Source_Data-sd.pdf]

Fig 5D

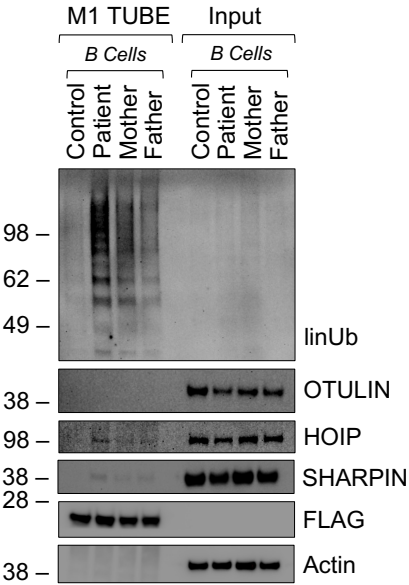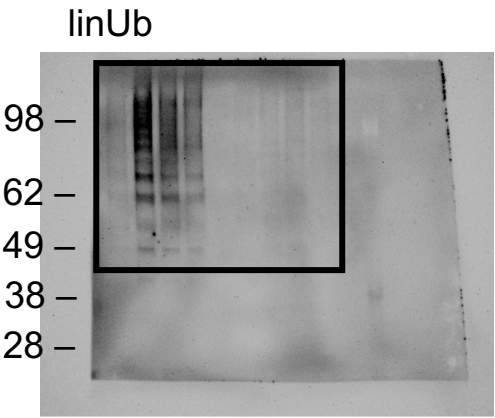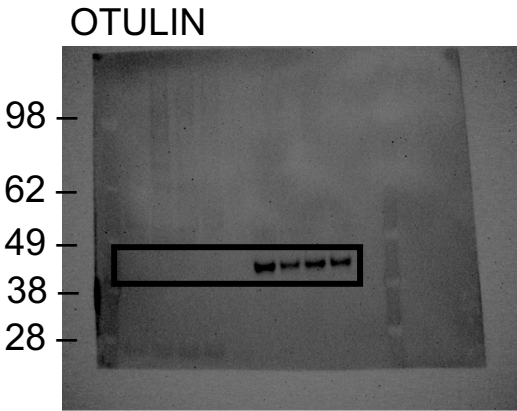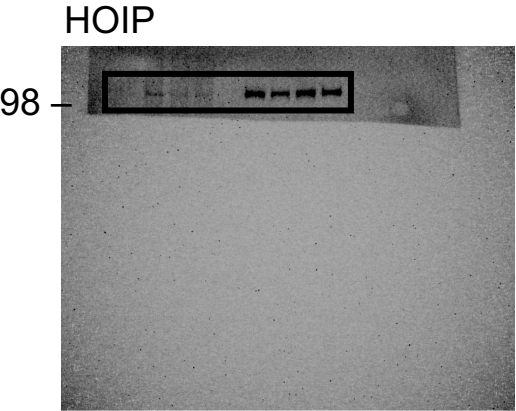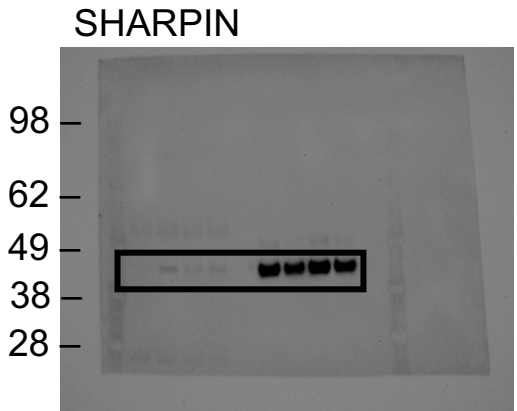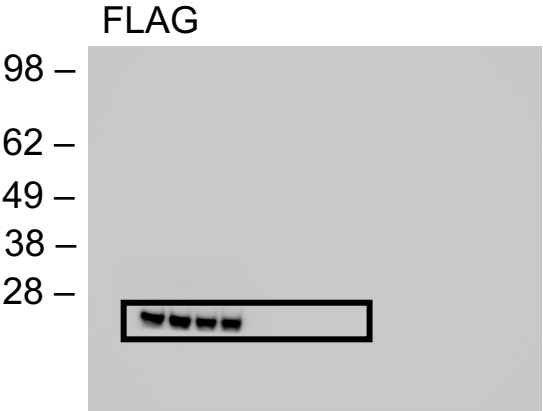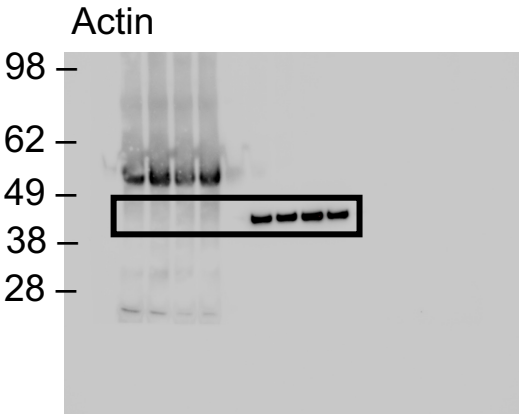

Supplement: Supplementary file 6 — Source Data for Figure 5 [file EMMM-14-e14901-s004.zip › EMM-2021-14901-V3-Figure_5D_Source_Data-sd.pdf]

Fig 6A

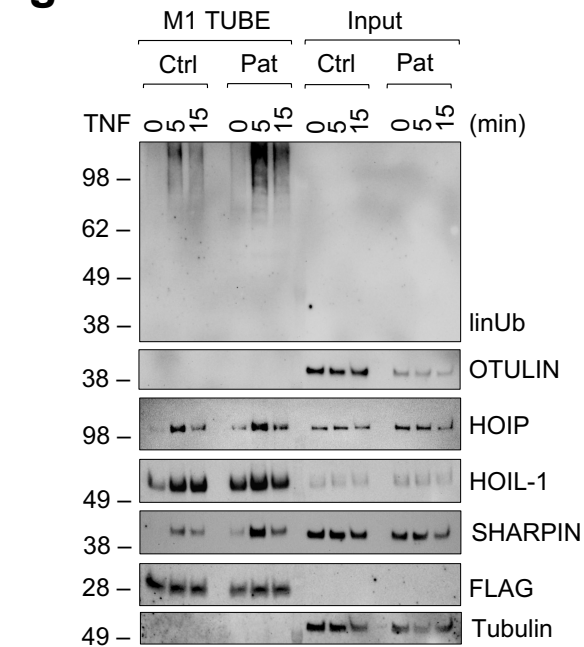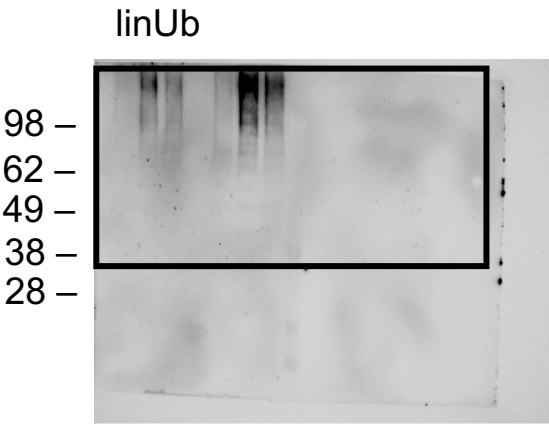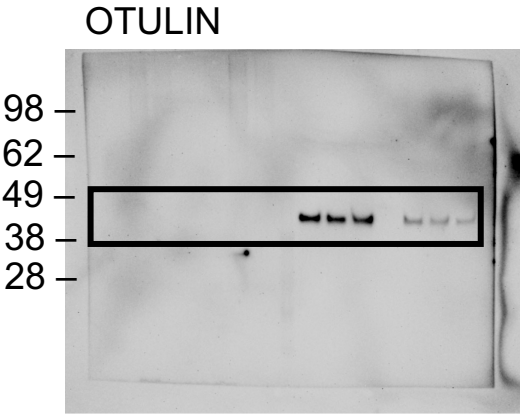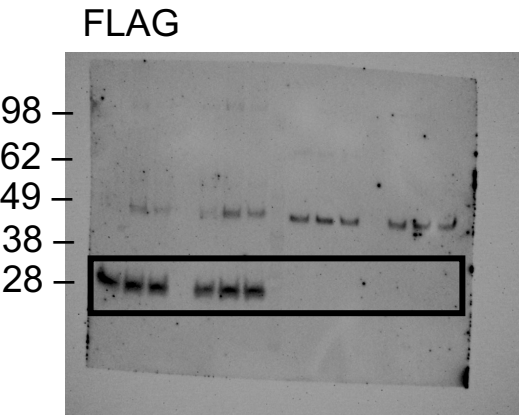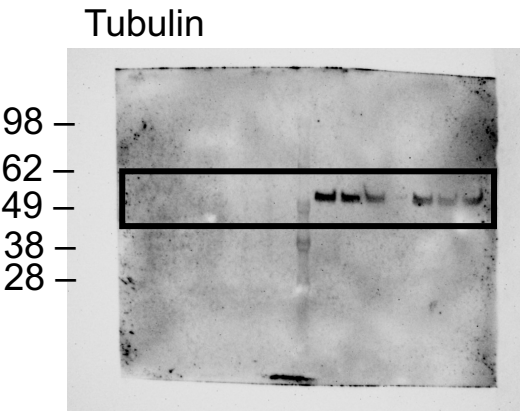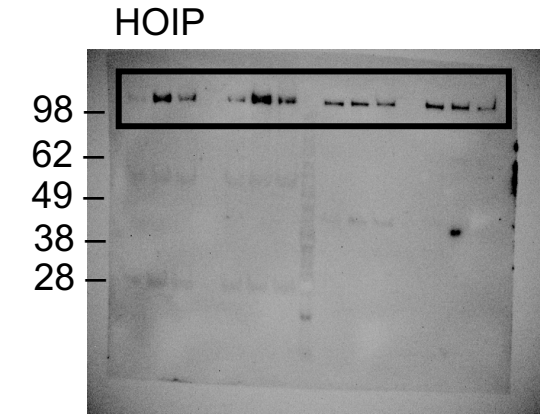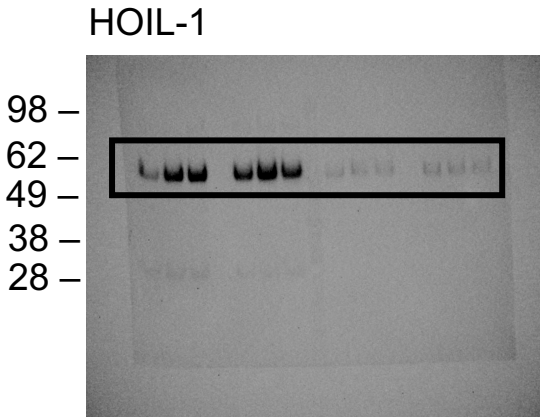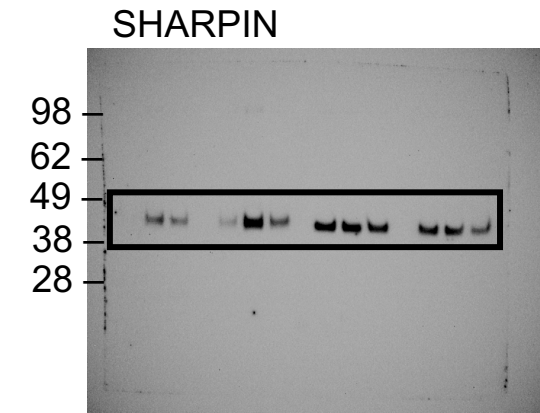

Supplement: Supplementary file 7 — Source Data for Figure 6 [file EMMM-14-e14901-s002.zip › EMM-2021-14901-V3-Figure_6A_Source_Data-sd.pdf]

Fig 6B

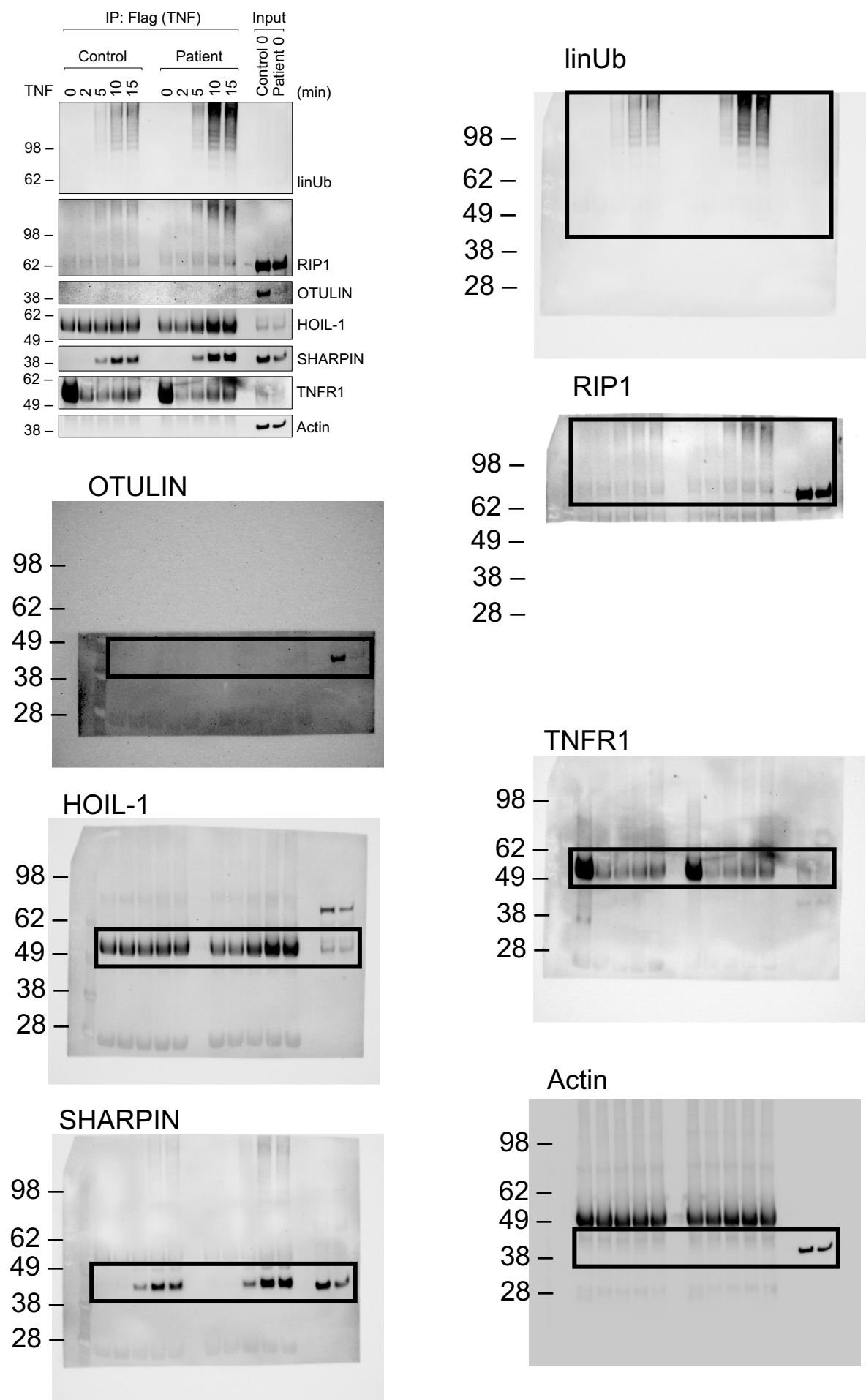

Supplement: Supplementary file 7 — Source Data for Figure 6 [file EMMM-14-e14901-s002.zip › EMM-2021-14901-V3-Figure_6B_Source_Data-sd.pdf]

Fig 6C

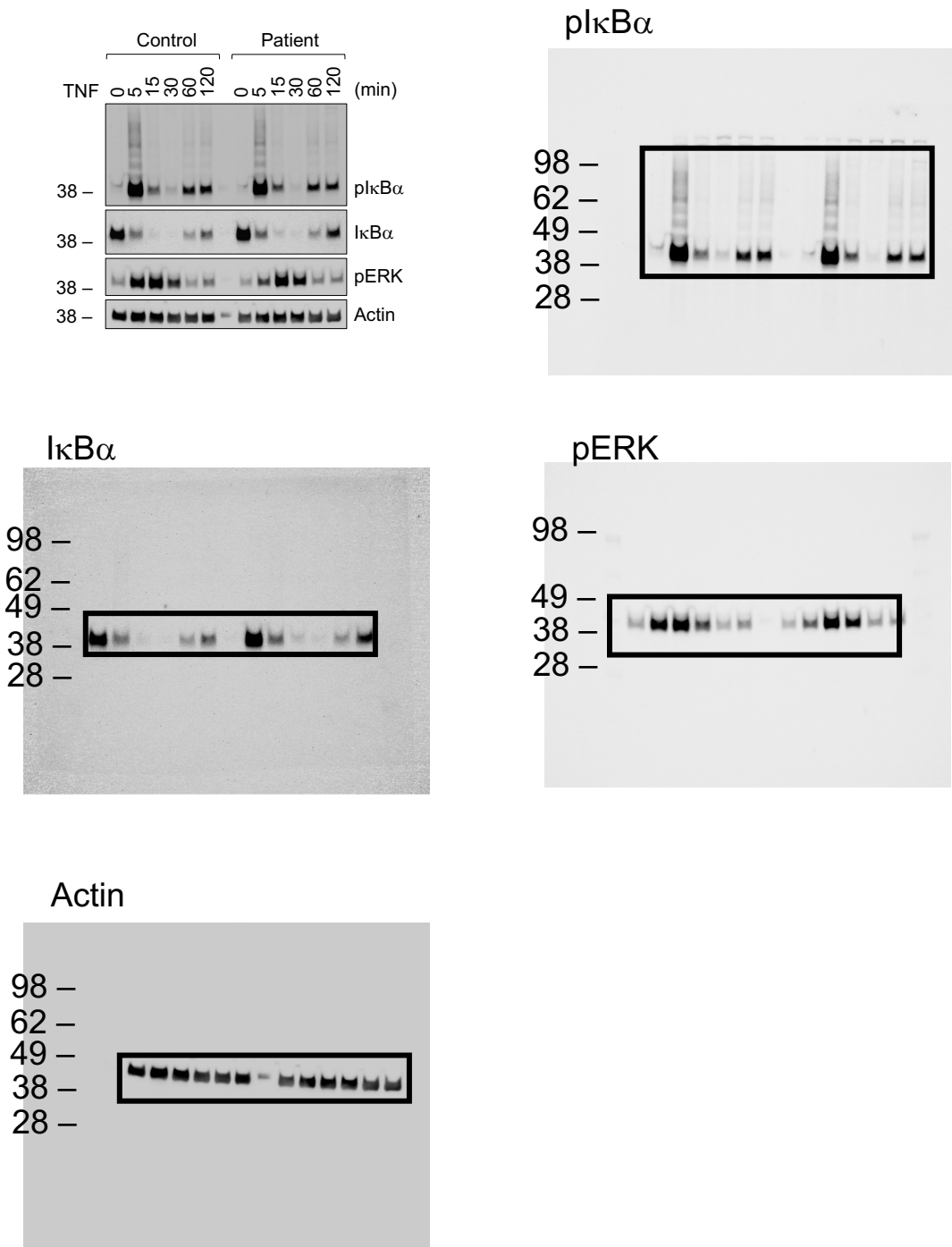

Supplement: Supplementary file 7 — Source Data for Figure 6 [file EMMM-14-e14901-s002.zip › EMM-2021-14901-V3-Figure_6C_Source_Data-sd.pdf]

### Fig 6E

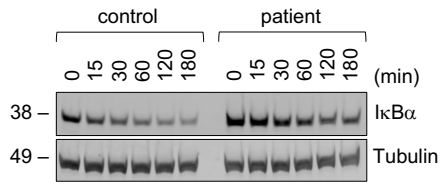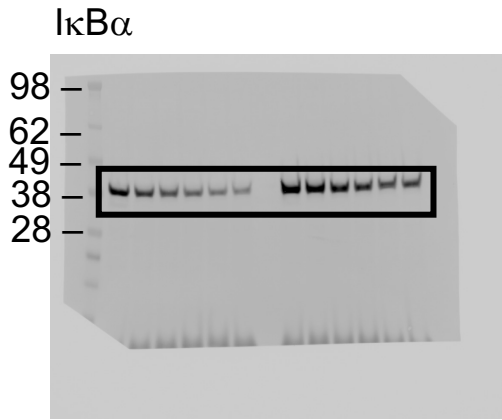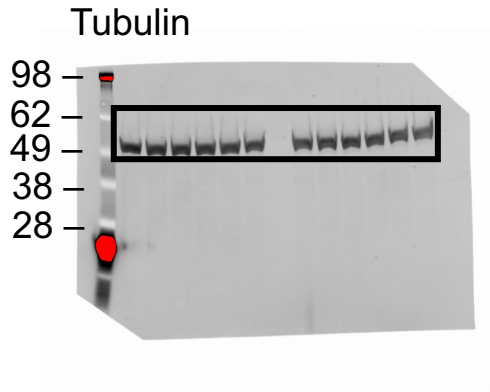

Supplement: Supplementary file 7 — Source Data for Figure 6 [file EMMM-14-e14901-s002.zip › EMM-2021-14901-V3-Figure_6E_Source_Data-sd(1).pdf]
