# Supplementary material for: Compound heterozygous variants in OTULIN are associated with fulminant atypical late‐onset ORAS
Source: EMBO Mol Med. 2022 Feb 16;14(3):e14901. doi: 10.15252/emmm.202114901 (PMC8899767; doi:10.15252/emmm.202114901)
Supplement: Supplementary file 8 [file EMMM-14-e14901-s001.pdf]

EMBO Molecular Medicine

Compound heterozygous variants in OTULIN are associated with fulminant atypical late-onset ORAS

Julia Zinngrebe, Barbara Moepps, Thomas Monecke, Peter Gierschik, Ferdinand Schlichtig, Thomas Barth, Gudrun Strauss, Elena Boldrin, Carsten Posovszky, Ansgar Schulz, Ortraud Beringer, Eva Rieser, Eva-Maria Jacobsen, Myriam Lorenz, Klaus Schwarz, Ulrich Pannicke, Henning Walczak, Dierk Niessing, Catharina Schuetz, Pamela Fischer-Posovszky, and Klaus-Michael Debatin
DOI: 10.15252/emmm.202114901

Corresponding author: Pamela Fischer-Posovszky (pamela.fischer@uniklinik-ulm.de)

Review Timeline:

Submission Date:	26th Jul 21
Editorial Decision:	31st Aug 21
Revision Received:	31st Dec 21
Editorial Decision:	17th Jan 22
Revision Received:	21st Jan 22
Accepted:	24th Jan 22

Editor: Zeljko Durdevic

Transaction Report:

(Note: With the exception of the correction of typographical or spelling errors that could be a source of ambiguity, letters and reports are not edited. Depending on transfer agreements, referee reports obtained elsewhere may or may not be included in this compilation. Referee reports are anonymous unless the Referee chooses to sign their reports.)

31st Aug 2021

Dear Dr. Fischer-Posovszky,

Thank you for the submission of your manuscript to EMBO Molecular Medicine. We have now received feedback from the three reviewers who agreed to evaluate your manuscript. As you will see from the reports below, the referees acknowledge the interest of the study but also raise important critique that should be addressed in a major revision.

Further consideration of a revision that addresses reviewers' concerns in full will entail a second round of review. EMBO Molecular Medicine encourages a single round of revision only and therefore, acceptance or rejection of the manuscript will depend on the completeness of your responses included in the next, final version of the manuscript. For this reason, and to save you from any frustrations in the end, I would strongly advise against returning an incomplete revision.

We would welcome the submission of a revised version within three months for further consideration. However, we realize that the current situation is exceptional on the account of the COVID-19/SARS-CoV-2 pandemic. Please let us know if you require longer to complete the revision.

I look forward to seeing a revised form of your manuscript. Use this link to login to the manuscript system and submit your revision: Link Not Available

Yours sincerely,

Zeljko Durdevic

Zeljko Durdevic
Editor
EMBO Molecular Medicine

***** Reviewer's comments *****

Referee #1 (Remarks for Author):

In this study, the authors use whole-exome sequencing to identify two new OTULIN variants (pW167S and p.M86I) in a compound heterozygous 7-year-old male suffering from an episode of severe autoinflammatory disease. The in silico and functional, cellular analyses of the two new variants revealed a partial loss of function of OTULIN in regulating M1-linked ubiquitin chains and in controlling innate immune signalling and cytokine production, and the authors conclude that the compound heterozygosity and partial loss of OTULIN function leads to an atypical and late-onset type of OTULIN-Related Autoinflammatory Syndrome (ORAS).

The findings in this study are interesting and identify a new ORAS family and two new mutations in OTULIN associated with ORAS. Importantly, it is the first report of atypical late-onset ORAS, which indicates a broader spectrum of clinical manifestations as well as a genotype-to-phenotype relationship for this recently identified, rare inflammatory disease. In addition, the discovery - although minor - that even heterozygous carriers of OTULIN mutations can exhibit signs of dysregulated M1-linked ubiquitin signalling is very intriguing.

I like this study a lot. Generally, it is well-executed and well-written, and the main conclusions are supported by data from well-performed and well-controlled experiments. The main findings in this manuscript expand the clinical/genetic understanding of ORAS and OTULIN. Mechanistically, the manuscript provides few new insights to OTULIN/LUBAC (dys)function, but rather builds on and strengthens mechanisms previously reported by others, primarily Damgaard/Komander, or themselves (Walczak). As there are so few reports of ORAS and its disease mechanisms, the insights from the mechanistic signalling experiments are interesting and an important contribution to the field. The combination of clinical and genetic analyses with insights into molecular disease mechanisms from patient-derived cells makes the manuscript highly relevant to the readership of EMBO Mol Med.

Overall, the study is an important and quite complete story as it stands. However, I have some questions and suggestions that the authors should address to further strengthen the manuscript:

1. In the abstract, p. 3, l. 40, the authors write " (p.W167S/p.M86I) in the OTULIN gene". The authors could consider rewriting this sentence as it is a bit intrinsically contradictory to refer to protein residue mutations in a gene.

2. The introduction is generally well-written, but I think the description of ORAS and the reported clinical manifestations are probably on the shorter side. For instance, there is no mention of the possible liver phenotype reported by Damgaard et al, CDD 2020 (PMID: 32231246) and Verboom et al. Cell Rep 2020 (PMID: 32075762) (although the latter only includes data from mice). This could be interesting to include and discuss considering the obesity in the reported patient. For a more complete introduction to ORAS, the authors could consider citing Jahan et al, CDD 2021 (PMID: 33441937), which includes a detailed review on ORAS.

3. Were any analyses of the patient's liver or liver function performed, e.g. ALT/AST measurements?

4. Are there any indications that the obesity in the reported patient is related to the identified OTULIN mutations or other identified genetic variants?

5. Fig 1A indicates that the patient was effectively treated with adalimumab. However, this is not mentioned or described anywhere in the main text. It is just mentioned that the patient is discharged in good condition after a two-month hospitalisation. It would be useful if the authors could describe the adalimumab treatment of the patient, e.g. dose, frequency, efficiency, etc. Did the autoinflammatory symptoms subside before or after adalimumab treatment?

6. P. 10, ll. 136-138: "WES revealed no other homozygous, compound heterozygous or pathogenic variants likely to explain the observed disease phenotype." The authors should provide a (supplementary) table with the other genetic variants identified in the patient.

7. It is surprising that proteasome inhibition cannot rescue the reduction in OTULIN protein levels observed in the patient cells. The authors used two antibodies to "exclude" that the apparent reduction in OTULIN levels is due to reduced antibody binding. However, without assessing the binding of the antibodies to recombinant OTULIN protein with the patient mutations, it cannot be "excluded", as the authors state, that the mutations impact the binding of the antibodies. I think the authors' experimental approach here is sensible and I think it is unlikely that the mutations would impair binding that the observed extent. However, given that the mutants do not appear to be rescued by proteasome inhibition, I think the authors should either perform the experiments on recombinant protein to fully exclude any impact of the mutations on the antibody binding or alternatively loosen or qualify their statements and conclusions accordingly.

8. Did the authors try to rescue the OTULIN levels using inhibitors of lysosomal degradation, e.g. Bafilomycin A?

9. P. 12, l. 165: "As OTULIN is known to prevent auto-ubiquitination of LUBAC (Draber, Kupka et al., 2015)...". This is true but it is not the only reported function of OTULIN (see point 11 below). I would suggest that the authors consider qualifying this statement to include other reported functions (e.g. see reviews from Weinelt and van Wijk, CDD 2021 (PMID: 33288901) or Verboom et al, Trends in Immunology 2021 (PMID: 34074601)), or alternatively loosening their statement a bit for balance, e.g. "One function of OTULIN is to prevent auto-ubiquitination of LUBAC" or something along those lines.

10. The in silico analysis and discussion of the impact of the identified OTULIN mutations on the structure and function of OTULIN (pp. 12-14) is detailed, well-explained, and generally well-argued. However, the paper would benefit significantly from just some form of experimental evidence to support the analyses and conclusions in this paragraph, e.g. analysis of the effect of the mutations on OTULIN's ability to bind M1-linked ubiquitin.

11. The overall effect of the identified OTULIN mutations on innate immune signalling, including TNF, in fibroblasts, B cells, and monocytes/macrophages are overall in accordance with previous reports on the effect of OTULIN deficiency in these cell types (Damgaard 2016, 2019; Heger 2018). However, one thing the authors do not analyse is the potential increase in basal NF-kB activity as reported in the literature, e.g. by Damgaard (2016, 2019) and van Wijk, Nature Microbiol 2017. To strengthen Fig 5 and Fig S3, the authors could perform an analysis of basal IkBa stability (e.g. CHX chase) in fibroblasts and B cells (and monocytes/macrophages or PBMCs if available) to assess if the increased basal TNF production correlates with increased basal NF-kB activation.

12. In the discussion, the authors focus solely on LUBAC autoubiquitination as the explanation for the observed cellular phenotypes on TNF signalling. But the authors do not provide any evidence in the manuscript that there is increased autoubiquitination of LUBAC components. Actually, they provide evidence of increased ubiquitination of RIPK1 (Fig 5B), but they do not discuss or include this or any of the published reports that OTULIN can regulate ubiquitination of substrates other than LUBAC itself during signalling in their model (e.g. Fiil et al, Mol Cell 2013 or Keusekotten et al, Cell 2013, which report regulation of RIPK1 and TNFR1 ubiquitination by OTULIN). This appears a bit unbalanced, particularly since autoubiquitination of LUBAC due to OTULIN deficiency is reported to lead to marked LUBAC destabilisation and degradation (Heger 2018; Damgaard 2016, 2019; Verboom 2020), which the authors do not observe in the present study. The authors should balance their discussion and take other modes of action of OTULIN beyond LUBAC deubiquitination into account if they cannot provide clearer experimental evidence to support their current model. Similarly, I suggest the authors modify Fig 6 to at least include increased RIPK1 ubiquitination as their own data show.

13. P. 20, l. 360-361: the authors cite Ehsanipour et al for LUBAC deficiency, however, I think this is a miscitation. The authors probably meant to cite Wang et al, Genome Med 2013.

14. The very conclusion of the paper on page 21, lines 370-376, reads a bit strange to me. The fact that the authors, in accordance with other reports, conclude that the driver of ORAS is TNF secreted from active myeloid cells yet mention this as "of note" (as if this was of minor importance) only after describing the effect on the cell death, on which there is little effect and only in the case of the less clinically relevant CHX co-treatment, strikes me as a bit backwards. Why have the authors prioritised their conclusions like this?

Referee #2 (Remarks for Author):

The manuscript by Zinngrebe et al is a straightforward, very interesting and informative, well performed and written, case study of a further case of ORAS (OTULIN-related autoinflammatory syndrome). The authors identify a compound heterozygous carrier of OTULIN mutations, who unlike previous ORAS cases, has a delayed onset of symptoms, which are however highly severe and indeed, 'fulminant'. An explanation is provided as to why the ORAS oset is delayed: While the maternal W167S mutation results in severe rise of M1 chains in cells, the paternal OTULIN M86I mutant seems more subtle, and may retain some function. Together the data show yet again, how tightly controlled and sensitive to disturbance, the M1 ubiquitin chain system is in our cells.

One experiment I was missing, was a slightly deeper investigation into the observed instability of the protein in cells. Both mutations have the potential to destabilise OTULIN protein (the MG132 experiment hardly addresses this). Previous studies have shown the feasibility of biochemical analysis of OTULIN. Specifically, it seems trivial to express and purify OTULIN variant protein and study stability by thermal denaturation studies (as in eg Damgaard EMM 2018). It would be very interesting if the M86I mutant was thermally destabilised; this may compound its relatively low change in catalytic efficiency.

Apart from this, my comments are merely stylistic.

Page 11, ll165: I don't think that autoUb is LUBAC accounts for all M1 chains that are present in the patient cells. This should be rephrased and a updated with a more appropriate reference.

Page 14: expression of linear ubiquitin in cells: change 'expression' to 'amount'

Page 16, ll267: Gyrd-Hansens lab in Oxford has performed many similar studies and should be cited alongside Draber et al.

Page 19, ll328: The authors here describe three very different scenarios, and the discussion at this point, should dive into a few additional, important points.

Firstly, OTULIN loss is fundamentally different from (non-destabilising) OTULIN mutations. LUBAC, via HOIP, not only binds OTULIN, but also interacts with the CYLD/SPATA2 complex (4 papers in 2016, should be cited/included). If OTULIN is absent, the binding site on LUBAC is free to bind SPATA2/CYLD, which may have a functional consequence for LUBAC complexes (eg in terms of localisation, and recruitment to the TNFR-SC).

Second, the C129A mutant of OTULIN converted OTULIN to the highest affinity M1 chains binder present in cells, and since the protein was not destabilised, it took LUBAC out of circulation by reinforcing its autoubiquitination. This seems a special, non-physiological state that does not represent the previous or here described forms of ORAS.

In the scenario present in the here described patient, a non-destabilising OTULIN variant with lower catalytic efficiency, will still be able to bind to LUBAC and compete with CYLD/SPATA, but is unlikely to grossly affect LUBAC autoubiquitination and autoinhibition.

Fig. 3: The quality of the structure figures, esp for depiction of the M86I variant, should be improved. In C, there are sections that seem to float in free space; in B there seems to be unnecessary detail.

Referee #3 (Remarks for Author):

OTULIN is a deubiquitinating enzyme (DUB) that specifically cleaves linear ubiquitin linkage. Patients having mutations in OTULIN genes exhibited autoinflammatory disease called OTULIN-related autoinflammatory syndrome (ORAS). Although previously described ORAS patients have homozygous mutations of OTULIN genes, the authors reported an ORAS patient with compound heterozygous variants (W167S/M86I). The authors showed that cells derived from patient exhibited increased linear ubiquitylation of components of LUBAC, an enzyme complex specifically generate linear ubiquitin chains, and augmented cell death triggered by TNFalpha and cycloheximide, which are the characteristics of cells from ORAS patients. Although symptoms and clinical manifestations are different from those of ORAS patients reported previously, the reported patient can be categorized in ORAS clinically. However, biochemical analyses of the OTULIN mutants were not adequate enough to support the conclusion. Thus, the reviewer feels that this manuscript cannot be include high profile journal such as EMBO molecular medicine in the present form. The comments are listed below.

#1. Since substantial amount of linear ubiquitin chains can be detected in EBV-transformed B cells from patients, both M86I and W167S mutations may affect catalytic activity of OTULIN (Figure 4D). However, OTULIN M86I could cleave linear ubiquitin chains at the comparable level to OTULIN WT when expressed in A549cells (Figure 4A). Thus, it is worth examining the Kcat value of the mutants to show the effect of the mutations on OTULIN DUB activity.

#2. Although the authors claimed that reduction of the amount of OTULIN in patients may not be caused by augmented degradation by the proteasome (Supplementary Fig 1D), it was not convincing. The reviewer recommends the authors to examine the half-life of the OTULIN mutants by treating cells with cycloheximide in the presence or absence of proteasome inhibitors (cycloheximide chase analysis).

#3. It is worth examining whether the mutants can bind to linear ubiquitin or not.

#4. It is interesting to know the amount of OTULIN in EBV-transformed B cells from parents.

#5. The reviewer is curious to know whether linear ubiquitination of HOIL-1 also increased in patient cells.

Response to reviewer's comments

First of all, we would like to thank all referees for careful reading of our manuscript. Their insightful comments and constructive criticism helped us improving our manuscript. We hope that with the included changes we meet the expectations of all three referees.
We address the individual points of the referees in a point-by-point reply below – the reviewer's comments are indicated in black and our answers in blue.

Referee #1 (Remarks for Author):

In this study, the authors use whole-exome sequencing to identify two new OTULIN variants (pW167S and p.M86I) in a compound heterozygous 7-year-old male suffering from an episode of severe autoinflammatory disease. The in silico and functional, cellular analyses of the two new variants revealed a partial loss of function of OTULIN in regulating M1-linked ubiquitin chains and in controlling innate immune signalling and cytokine production, and the authors conclude that the compound heterozygosity and partial loss of OTULIN function leads to an atypical and late-onset type of OTULIN-Related Autoinflammatory Syndrome (ORAS).

The findings in this study are interesting and identify a new ORAS family and two new mutations in OTULIN associated with ORAS. Importantly, it is the first report of atypical late-onset ORAS, which indicates a broader spectrum of clinical manifestations as well as a genotype-to-phenotype relationship for this recently identified, rare inflammatory disease. In addition, the discovery - although minor - that even heterozygous carriers of OTULIN mutations can exhibit signs of dysregulated M1-linked ubiquitin signalling is very intriguing.

I like this study a lot. Generally, it is well-executed and well-written, and the main conclusions are supported by data from well-performed and well-controlled experiments. The main findings in this manuscript expand the clinical/genetic understanding of ORAS and OTULIN. Mechanistically, the manuscript provides few new insights to OTULIN/LUBAC (dys)function, but rather builds on and strengthens mechanisms previously reported by others, primarily Damgaard/Komander, or themselves (Walczak). As there are so few reports of ORAS and its disease mechanisms, the insights from the mechanistic signalling experiments are interesting and an important contribution to the field. The combination of clinical and genetic analyses with insights into molecular disease mechanisms from patient-derived cells makes the manuscript highly relevant to the readership of EMBO Mol Med.

We would like to thank this referee for his/her positive feedback and the in-depth reading of our manuscript!

Overall, the study is an important and quite complete story as it stands. However, I have some questions and suggestions that the authors should address to further strengthen the manuscript:

Point raised by reviewer:
1. In the abstract, p. 3, l. 40, the authors write " (p.W167S/p.M86I) in the OTULIN gene". The authors could consider rewriting this sentence as it is a bit intrinsically contradictory to refer to protein residue mutations in a gene.

Response and change in manuscript:
We changed the respective sentence of the abstract which now reads as follows:
Here, we describe an atypical form of ORAS with distinct clinical manifestation of the disease caused by two new compound heterozygous variants (c.258G>A (p.M86I)/c.500G>C (p.W167S)) in

the OTULIN gene in a seven-year-old affected by a life-threatening autoinflammatory episode with sterile abscess formation.

Point raised by reviewer:
2. The introduction is generally well-written, but I think the description of ORAS and the reported clinical manifestations are probably on the shorter side. For instance, there is no mention of the possible liver phenotype reported by Damgaard et al, CDD 2020 (PMID: 32231246) and Verboom et al. Cell Rep 2020 (PMID: 32075762) (although the latter only includes data from mice). This could be interesting to include and discuss considering the obesity in the reported patient. For a more complete introduction to ORAS, the authors could consider citing Jahan et al, CDD 2021 (PMID: 33441937), which includes a detailed review on ORAS.

Response and change in manuscript:
We thank the reviewer for these helpful suggestions. The liver phenotype described in the above-named publications is now included in the introduction (p. 5, l. 90 and following):
One ORAS patient carrying a homozygous missense mutation in OTULIN (Damgaard, Walker et al., 2016) additionally suffered from steatosis and hepatocyte degeneration with abnormal liver values (Damgaard, Jolin et al., 2020) suggesting that functioning OTULIN is also essential for liver health. This is further supported by the fact that mice with liver-specific deletion of OTULIN show a similar disease phenotype with liver inflammation and apoptosis ultimately leading to formation of hepatocellular carcinoma (Damgaard et al., 2020, Verboom, Martens et al., 2020).

We now also cite Jahan et al., CDD, 2021 in the introduction (p.4, lines 76 and 77):
Dysregulation of linear ubiquitin linkages is associated with numerous human diseases, including immune disorders, cancer, and neurodegeneration (Jahan, Elbaek et al., 2021).

In addition, we included the patient's liver values (Fig EV1A) and ultrasound examination (Fig EV1B) which indeed revealed that the patient had since developed grade II steatosis although the liver parenchyma initially looked normal (see point #3 of reviewer #1).

This is also discussed in the revised version of the manuscript as follows (p. 22, l. 461 and following):
In addition, one ORAS patient was identified to suffer from progressive steatotic liver disease (Damgaard et al., 2020) suggesting that OTULIN is crucial for liver homeostasis. This assumption was confirmed in mice with liver-specific OTULIN deficiency which suffered from steatohepatitis, fibrosis and hepatocellular carcinoma (Damgaard et al., 2020, Verboom et al., 2020). Although our patient showed elevated liver enzymes during his autoinflammatory episode (Figure EV1A), the laboratory values normalized in the further course and always remained within the normal range during follow-up. Routine abdominal ultrasound examination, however, revealed that the patient had since developed sonographic alterations of the liver indicative of grade II hepatic steatosis (Figure EV1B) whilst liver function tests remained normal. Whether the steatosis is caused by the patient's obesity, a risk factor for liver steatosis (Guzzaloni, Grugni et al., 2000), or directly related to his ORAS, and further fueled by his treatment with Adalimumab (Haas, Chevalier et al., 2017), cannot be clarified with certainty at the moment. That hepatocytes are vulnerable to changes in the amount of linear ubiquitination is further highlighted by the fact that liver tumorigenesis is also promoted in mice with liver-specific deletion of HOIP (Shimizu, Peltzer et al., 2017). Future studies will be required to assess how exactly the interplay of OTULIN and LUBAC determines liver health to be able to predict whether OTULIN variants identified in this patient, and others, bear the risk of increased liver tumorigenesis. In any case, ORAS patients should be examined regularly for the development of morphological and functional liver abnormalities.

Point raised by reviewer:
3. Were any analyses of the patient's liver or liver function performed, e.g. ALT/AST measurements?

Response and change in manuscript:
AST (aspartate transaminase), ALT (alanine transaminase), GGT (γ-glutamyltransferase) and AP (alkaline phosphatase) measurements were performed over the course of the autoinflammatory episode and during follow-up of the patient. The data can now be found in Fig EV1A. Liver functionality was not affected at any time.
Ultrasound examination indeed revealed that the patient had since developed grade II steatosis although the liver parenchyma initially looked normal. This data is now included in Fig EV1B.

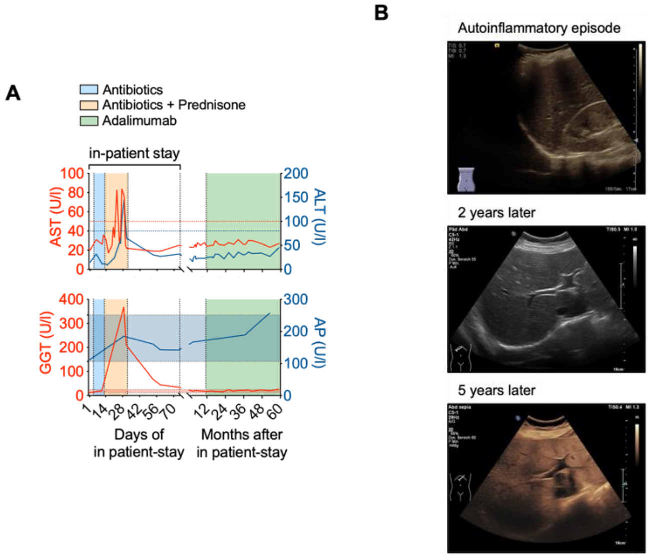

Figure EV1. Abnormal liver function test and development of steatosis hepatis grade II
(A) The course of the patient's liver enzymes AST (aspartate transaminase) and ALT (alanine transaminase) (upper panel) or GGT (g-glutamyltransferase) and AP (alkaline phosphatase) (lower panel) are depicted along the time axis with the upper limit of normal indicated by red and blue lines for AST and ALT and the normal range in shades of red and blue for GGT and AP, respectively. (B) Liver ultrasound B-mode images of the patient during the initial phase of autoinflammation and about 2 and 5 years thereafter display diffuse increase of liver echogenicity and finally slightly impaired appearance of portal vein wall and diaphragm indicative of steatosis hepatis grade II.

Point raised by reviewer:
4. Are there any indications that the obesity in the reported patient is related to the identified OTULIN mutations or other identified genetic variants?

Response:
There is no evidence yet that the patient's obesity is directly linked to his OTULIN variants.
Other identified homozygous and heterozygous single nucleotide variants (SNVs) and homozygous and heterozygous small insertions and deletions (InDel) are now listed in the Appendix Tables S4 – S7). Known obesity-related genes (Appendix Table S8; (Chesi & Grant, 2015, Rouillard, Gundersen et al., 2016, Warner, Jiang et al., 2021)) were not affected in the patient apart from *FAT1* (FAT atypical cadherin 1) and *WWOX* (WW Domain Containing Oxidoreductase) (Appendix Figure S2). The SNVs described to be associated with obesity are rs9923451 (16: 1677509940) and rs925642 (4: 187915860) for *WWOX* and *FAT1*, respectively (Wang, Li et al., 2011). The patient's *WWOX* and *FAT1* genes, however, showed a heterozygous single nucleotide variant (SNV) resulting in a missense mutation of yet unknown clinical impact.
Apart from the genetic aspects, anti-TNF treatment has been recently shown to be associated with excessive weight gain (Haas et al., 2017).

Change in manuscript (p. 21, l. 449 and following):

Whereas recently identified patients with ORAS presented with failure to thrive (Damgaard, Elliott et al., 2019, Damgaard et al., 2020, Damgaard et al., 2016, Zhou, Yu et al., 2016), this study's patient suffers from obesity. Whether the patient's obesity is due to his compound heterozygosity in OTULIN is currently unknown. Known obesity-related genes (Appendix Table S8; (Chesi & Grant, 2015, Rouillard et al., 2016, Warner et al., 2021)) were not affected in the patient apart from *FAT1* (FAT atypical cadherin 1) and *WWOX* (WW Domain Containing Oxidoreductase) (Appendix Figure S2). The single nucleotide variants (SNV) described to be associated with obesity are rs9923451 (16: 1677509940) and rs925642 (4: 187915860) for *WWOX* and *FAT1*, respectively (Wang et al., 2011). The patient's *WWOX* and *FAT1* genes, however, showed a heterozygous SNV resulting in a missense mutation of yet unknown clinical impact. Thus, the underlying cause for the patient's obesity remains currently uncertain.

Point raised by reviewer:
5. Fig 1A indicates that the patient was effectively treated with adalimumab. However, this is not mentioned or described anywhere in the main text. It is just mentioned that the patient is discharged in good condition after a two-month hospitalisation. It would be useful if the authors could describe the adalimumab treatment of the patient, e.g. dose, frequency, efficiency, etc. Did the autoinflammatory symptoms subside before or after adalimumab treatment?

Response:
We apologize for not having included this information in the initial submission. The patient was discharged in good condition after his autoinflammatory episode. Afterwards, WES was performed revealing the compound-heterozygous mutations in OTULIN. Based on this finding and on data from our in-vitro analysis, we decided to treat the patient with Adalimumab (30mg Adalimumab (Humira®) applied subcutaneously every 14 days) to prevent further potentially fatal autoinflammatory episodes. The treatment was started about 1 year after his autoinflammatory episode (indicated in Figure 1A). The dose of Adalimumab was adjusted according to body weight after 1 ½ years to 40mg.
Thus, overt autoinflammatory symptoms subsided before Adalimumab treatment. However, plasma protein levels of the patient differed from that of healthy controls before Adalimumab treatment (Figure EV5A) but, importantly, were reverted to normal after start of Adalimumab treatment.
Adalimumab treatment was monitored by determining the trough levels at regular intervals which remained within the required range of above 5µg/ml that is considered effective in other inflammatory diseases (e.g. inflammatory bowel disease) (Figure EV5B).

Change in manuscript (p. 17, l. 349 and following):
ORAS patients were identified to benefit from TNF blockers (Damgaard et al., 2019, Damgaard et al., 2016, Zhou et al., 2016) underlining the importance of TNF in the pathogenesis of the disease. Thus, this study's patient received 30mg Adalimumab applied subcutaneously every 14 days (Fig 1A) as preventive measure after WES revealed his compound heterozygosity in OTULIN (Fig 1E). The Adalimumab dosage was subsequently adjusted to 40mg. To assess whether Adalimumab treatment had a beneficial effect on the patient's inflammatory state we compared the patient's plasma protein profile before anti-TNF treatment and thereafter with plasma from two healthy controls (Fig EV5A). Whereas the patient's plasma protein profile differed from that of healthy controls before anti-TNF treatment with upregulation of C-X-C motif chemokine ligand (CXCL) 11, C-C motif chemokine ligand (CCL) 5, IL-18 and serpin E1, it was reverted to healthy condition after start of anti-TNF treatment. The Adalimumab concentration in the patient's serum has always been within therapeutic range (Fig EV5B) and importantly, the patient has not encountered any additional autoinflammatory episodes for four years.

Point raised by reviewer:

6. P. 10, ll. 136-138: "WES revealed no other homozygous, compound heterozygous or pathogenic variants likely to explain the observed disease phenotype." The authors should provide a (supplementary) table with the other genetic variants identified in the patient.

Response:

This information is now provided in the Appendix of the paper (Appendix Tables S4 – S7).

Point raised by reviewer:

7. It is surprising that proteasome inhibition cannot rescue the reduction in OTULIN protein levels observed in the patient cells. The authors used two antibodies to "exclude" that the apparent reduction in OTULIN levels is due to reduced antibody binding. However, without assessing the binding of the antibodies to recombinant OTULIN protein with the patient mutations, it cannot be "excluded", as the authors state, that the mutations impact the binding of the antibodies. I think the authors' experimental approach here is sensible and I think it is unlikely that the mutations would impair binding that the observed extent. However, given that the mutants do not appear to be rescued by proteasome inhibition, I think the authors should either perform the experiments on recombinant protein to fully exclude any impact of the mutations on the antibody binding or alternatively loosen or qualify their statements and conclusions accordingly.

Response and change in manuscript:

We expressed OTULIN^{WT}, OTULIN^{M86I} and OTULIN^{W167S} along with empty vector as control in A549 OTULIN KO cells and assessed OTULIN antibody binding on respective lysates of the different conditions. The results of this experiment can be found in Figure EV2B in the updated version of the manuscript. The paragraph was reworded accordingly.

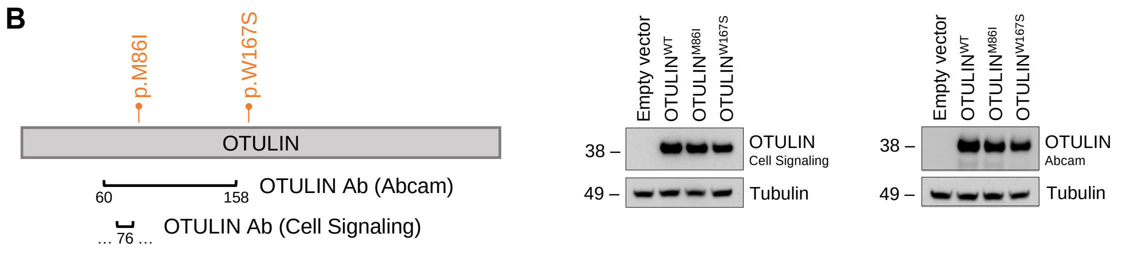

Figure EV2B. OTULIN antibodies by Abcam (antigen corresponding to AA 60 – 158) or by Cell Signaling (recombinant fragment surrounds S76 without spanning M86) were tested in parallel on A549 *OTULIN* KO cells transfected with different OTULIN constructs as indicated. One representative of two independent experiments is shown.

Point raised by reviewer:

8. Did the authors try to rescue the OTULIN levels using inhibitors of lysosomal degradation, e.g. Bafilomycin A?

Response and change in manuscript:

To address this point, we first determined the half-life of recombinant wildtype and variant OTULIN as suggested by Reviewer #3 (Fig EV2D). In a next step, we then used A549 OTULIN KO cells transfected with OTULIN^{WT}, OTULIN^{M86I} or OTULIN^{W167S} and treated the cells with either Cycloheximide (CHX) alone or in combination with the proteasome inhibitor Bortezomib (BTZ) or the inhibitor of lysosomal degradation Bafilomycin A1 (Baf A1). Neither application of BTZ nor of Baf A1 rescued expression of recombinant OTULIN although MCL-1 expression and LC3 expression

used as positive controls were stabilized by BTZ and Baf A1, respectively. The results of this experiment can be found in Fig EV2E.

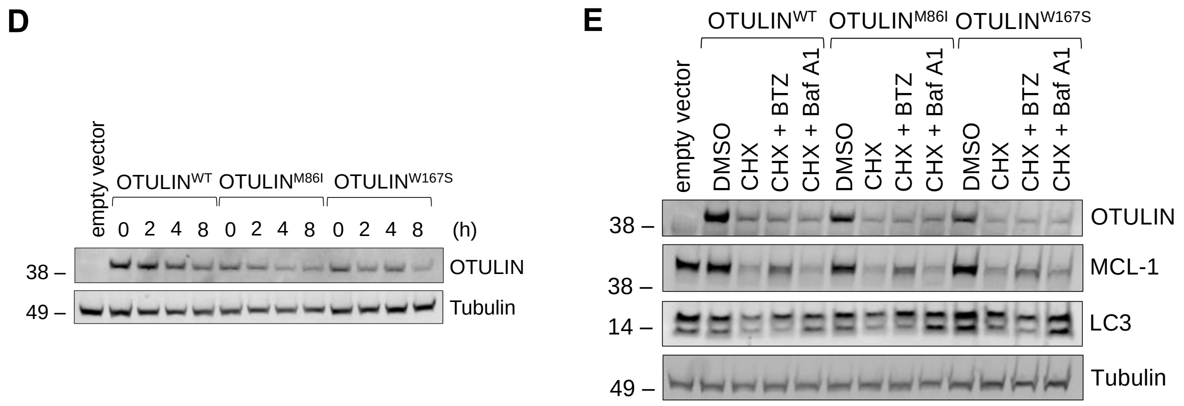

Figure EV2D and EV2E. (D) A549 *OTULIN* KO cells were transfected with the different OTULIN constructs as indicated. The following day, cells were treated with 50µg/ml cycloheximide (CHX) for the indicated times, harvested and analyzed by Western Blot for OTULIN protein expression. Tubulin served as loading control. One representative of three independent experiments is shown. (E) A549 *OTULIN* KO cells were transfected with the different OTULIN constructs as indicated. The following day, cells were treated with 50µg/ml cycloheximide (CHX) alone or in combination with 1µM Bortezomib (BTZ) or 1µM Bafilomycin A1 (Baf A1) for 8 hours or left untreated (DMSO). Expression of the proteins indicated was analyzed by Western Blot. One representative of three independent experiments is shown.

Point raised by reviewer:
9. P. 12, l. 165: "As OTULIN is known to prevent auto-ubiquitination of LUBAC (Draber, Kupka et al., 2015)...". This is true but it is not the only reported function of OTULIN (see point 11 below). I would suggest that the authors consider qualifying this statement to include other reported functions (e.g. see reviews from Weinelt and van Wijk, CDD 2021 (PMID: 33288901) or Verboom et al, Trends in Immunology 2021 (PMID: 34074601)), or alternatively loosening their statement a bit for balance, e.g. "One function of OTULIN is to prevent auto-ubiquitination of LUBAC" or something along those lines.

Response and change in manuscript:
We thank the reviewer for pointing this out. We rephrased the respective paragraph which now reads as follows (p. 10, l. 186 and following):
One function of OTULIN is the cleavage of linear ubiquitin linkages (Verboom, Hoste et al., 2021, Weinelt & van Wijk, 2021). Numerous studies demonstrated that OTULIN downregulation or knockout as well as the expression of certain OTULIN variants lead to accumulation of linear ubiquitin linkages in cells (Damgaard et al., 2016, Draber, Kupka et al., 2015, Elliott, Nielsen et al., 2014, Fiil, Damgaard et al., 2013, Heger, Wickliffe et al., 2018, Hrdinka, Fiil et al., 2016, Keusekotten, Elliott et al., 2013, Rivkin, Almeida et al., 2013, van Wijk, Fricke et al., 2017, Zhou et al., 2016).

Point raised by reviewer:
10. The in silico analysis and discussion of the impact of the identified OTULIN mutations on the structure and function of OTULIN (pp. 12-14) is detailed, well-explained, and generally well-argued. However, the paper would benefit significantly from just some form of experimental evidence to support the analyses and conclusions in this paragraph, e.g. analysis of the effect of the mutations on OTULIN's ability to bind M1-linked ubiquitin.

Response and change in manuscript:
To investigate whether binding of OTULIN to linear di-ubiquitin is compromised by the two variants p.M86I and p.W167S, we generated recombinant catalytically inactive versions of OTULIN^{WT},

OTULIN^{M86I}, and OTULIN^{W167S} by substituting the active-site cysteine at position 129 with alanine (OTULIN^{C129A}, OTULIN$^{C129A;M86I}$ and OTULIN$^{C129A;W167S}$; Appendix Figure S3). Performing a surface plasmon resonance assay we found that both, OTULIN^{M86I} and OTULIN^{W167S}, have a lower affinity to linear di-ubiquitin than OTULIN^{WT} (OTULIN^{C129A}: 3.3µM ± 0.53µM, OTULIN$^{C129A;M86I}$: 4.9µM ± 0.34µM; OTULIN$^{C129A;W167S}$: 5.1µM ± 0.37µM) (Fig 4B).

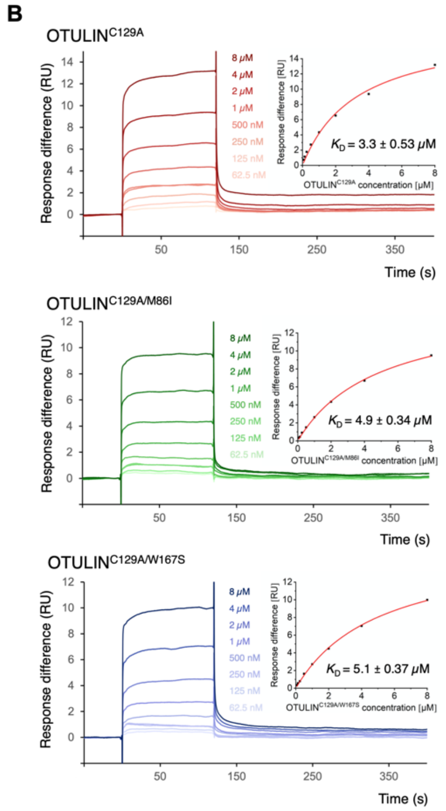

Figure 4B. SPR measurements and steady-state binding curves with calculated dissociation constants (K_d) after injection of a concentration series of OTULIN^{C129A}, OTULIN$^{C129A/M86I}$ or OTULIN$^{C129A/W167S}$ to CM5-immobilized di-ubiquitin chains.

Point raised by reviewer:

11. The overall effect of the identified OTULIN mutations on innate immune signalling, including TNF, in fibroblasts, B cells, and monocytes/macrophages are overall in accordance with previous reports on the effect of OTULIN deficiency in these cell types (Damgaard 2016, 2019; Heger 2018). However, one thing the authors do not analyse is the potential increase in basal NF-kB activity as reported in the literature, e.g. by Damgaard (2016, 2019) and van Wijk, Nature Microbiol 2017. To strengthen Fig 5 and Fig S3, the authors could perform an analysis of basal IkBa stability (e.g. CHX chase) in fibroblasts and B cells (and monocytes/macrophages or PBMCs if available) to assess if the increased basal TNF production correlates with increased basal NF-kB activation.

Response and change in manuscript:

As suggested by this reviewer, we assessed basal NF-κB activity by cycloheximide (CHX) chase analysis. IκBα was more stable in patient-derived B cells and fibroblasts as compared to control (Fig 6E and Fig EV4D). Moreover, we also determined basal TNF secretion in patient-derived and control B cells by ELISA (Fig 6F). Patient-derived B cells secreted less TNF than control B cells further supporting the observations from the CHX chase analysis. In ORAS patients, monocytes are known to produce and secrete more TNF than healthy controls (Damgaard et al., 2019, Damgaard et al., 2016, Zhou et al., 2016). Unfortunately, we could not perform the CHX chase analysis in patient-derived monocytes/macrophages or PBMCs as there was no material left from the time before start of anti-TNF therapy.

E

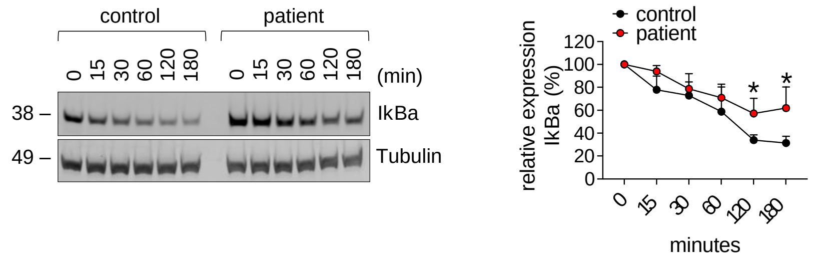

Figure 6E. B cells were incubated with 50µg/ml cycloheximide (CHX) for the indicated times and subjected to analysis by western blot. One representative (left panel) out of 4 individual experiments analyzed by densitometry (right panel) is shown. Data are presented as mean ± SD. *, P = 0.02, unpaired t-test.

D

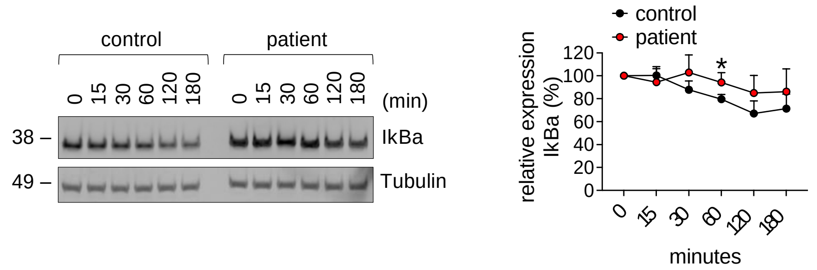

Figure EV4D. Fibroblasts were incubated with 50µg/ml cycloheximide (CHX) for the indicated times and subjected to analysis by western blot. One representative (left panel) out of 4 individual experiments analyzed by densitometry (right panel) is shown. Data are presented as mean ± SD. *, P = 0.02, unpaired t-test.

F

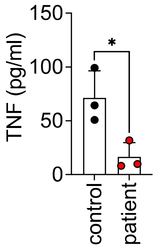

Figure 6F. Patient-derived and control B cells were cultivated for 24 hours and TNF was determined by ELISA. Data are presented as mean ± SD of 3 individual experiments performed in two technical replicates; *, P = 0.028; unpaired t-test.

Point raised by reviewer:

12. In the discussion, the authors focus solely on LUBAC autoubiquitination as the explanation for the observed cellular phenotypes on TNF signalling. But the authors do not provide any evidence in the manuscript that there is increased autoubiquitination of LUBAC components. Actually, they provide evidence of increased ubiquitination of RIPK1 (Fig 5B), but they do not discuss or include this or any of the published reports that OTULIN can regulate ubiquitination of substrates other than LUBAC itself during signalling in their model (e.g. Fiil et al, Mol Cell 2013 or Keusekotten et al, Cell 2013, which report regulation of RIPK1 and TNFR1 ubiquitination by OTULIN). This appears a bit unbalanced, particularly since autoubiquitination of LUBAC due to OTULIN deficiency is reported to lead to marked LUBAC destabilisation and degradation (Heger 2018; Damgaard 2016, 2019; Verboom 2020), which the authors do not observe in the present study. The authors should balance their discussion and take other modes of action of OTULIN beyond LUBAC deubiquitination into account if they cannot provide clearer experimental evidence to support their current model. Similarly, I suggest the authors modify Fig 6 to at least include increased RIPK1 ubiquitination as their own data show.

Response:

We thank this reviewer for the constructive criticism.

As shown in the M1 TUBE experiments in Figures 2B, 4D and 5A, LUBAC components, but not OTULIN, are pulled down together with linear ubiquitin linkages. These results are in line with data published by Heger *et al.*, Nature, 2018 (see Figure 2a), in which they show that catalytically inactive OTULIN^{C129A} results in increased LUBAC autoubiquitination. As we obtained similar results in our system, we conclude that OTULIN variants in patient-derived cells lead to enhanced LUBAC

autoubiquitination. However, this reviewer is right that LUBAC components are not destabilized in patient-derived cells. Therefore, we have taken this criticism on board and rephrased the respective part in the discussion section.

We agree with this reviewer that our finding of increased RIPK1 ubiquitination should be emphasized. We now discuss this in the revised version of the manuscript along with above-mentioned citations, and, in addition, we have included the increased ubiquitination of RIPK1 in the visual abstract (former Figure 6) as suggested by this reviewer.

Change in manuscript (p. 19, l. 398)**:**
OTULIN deficiency or expression of catalytically inactive OTULIN^{C129A} cause increased LUBAC autoubiquitination and degradation (Damgaard et al., 2019, Damgaard et al., 2016, Heger et al., 2018, Verboom et al., 2020). In patient-derived cells, LUBAC components were pulled down together with increased amounts of linear ubiquitin linkages in patient-derived cells (Fig 2B and 6A) suggestive of enhanced LUBAC autoubiquitination in the patient. However, expression levels of LUBAC components were not altered (Fig 2A) and, in addition, we observed augmented LUBAC recruitment to the TNFR1-SC (Fig 6B) along with increased ubiquitination of the TNFR1-SC component RIPK1 (Fig 6B). This is in line with previous reports demonstrating that OTULIN can regulate ubiquitination of substrates other than LUBAC, *e.g.* RIPK1 and TNFR1 (Fiil et al., 2013, Keusekotten et al., 2013).

Point raised by reviewer:
13. P. 20, l. 360-361: the authors cite Ehsanipour et al for LUBAC deficiency, however, I think this is a miscitation. The authors probably meant to cite Wang et al, Genome Med 2013.

Response:
We apologize for this mistake and thank the referee for pointing this out. The miscitation was replaced with the correct one (Wang, Kim et al., 2013).

Point raised by reviewer:
14. The very conclusion of the paper on page 21, lines 370-376, reads a bit strange to me. The fact that the authors, in accordance with other reports, conclude that the driver of ORAS is TNF secreted from active myeloid cells yet mention this as "of note" (as if this was of minor importance) only after describing the effect on the cell death, on which there is little effect and only in the case of the less clinically relevant CHX co-treatment, strikes me as a bit backwards. Why have the authors prioritised their conclusions like this?

Response:
With this formulation we did not want to downplay the importance of TNF production in monocytes, but on the contrary, tried to emphasize it. Since we agree with the reviewer that TNF produced by monocytes is of great importance for the disease pathology and given that our original wording was misleading, we have reworded this paragraph. We hope to thereby meet the expectations of this reviewer.

Change in manuscript (p. 23, l. 486):
Whereas the symptoms identified in the patient are distinct from symptoms identified in patients with classical ORAS, we found similarities between classical and atypical ORAS on the molecular level: Monocytes showed enhanced TNF production as reported previously, confirming that TNF secreted from myeloid cells is the primary driver of the inflammation in patients with ORAS (Damgaard et al., 2019, Damgaard et al., 2016) (Fig 6). Moreover, TNF-induced gene activation was downregulated, but TNF-mediated cell death enhanced in the presence of CHX in patient-derived

fibroblasts, in line with previous reports (Damgaard et al., 2019, Damgaard et al., 2016, Zhou et al., 2016) (Fig 6).

Referee #2 (Remarks for Author):

The manuscript by Zinngrebe et al is a straightforward, very interesting and informative, well performed and written, case study of a further case of ORAS (OTULIN-related autoinflammatory syndrome). The authors identify a compound heterozygous carrier of OTULIN mutations, who unlike previous ORAS cases, has a delayed onset of symptoms, which are however highly severe and indeed, 'fulminant'. An explanation is provided as to why the ORAS oset is delayed: While the maternal W167S mutation results in severe rise of M1 chains in cells, the paternal OTULIN M86I mutant seems more subtle, and may retain some function. Together the data show yet again, how tightly controlled and sensitive to disturbance, the M1 ubiquitin chain system is in our cells.

Point raised by reviewer:
One experiment I was missing, was a slightly deeper investigation into the observed instability of the protein in cells. Both mutations have the potential to destabilise OTULIN protein (the MG132 experiment hardly addresses this). Previous studies have shown the feasibility of biochemical analysis of OTULIN. Specifically, it seems trivial to express and purify OTULIN variant protein and study stability by thermal denaturation studies (as in eg Damgaard EMM 2018). It would be very interesting if the M86I mutant was thermally destabilised; this may compound its relatively low change in catalytic efficiency.

Response:
To assess how the variants p.M86I and p.W167S affect OTULIN's intrinsic stability, we generated recombinant wildtype ($OTULIN^{WT}$) and variant OTULIN ($OTULIN^{M86I}$ and $OTULIN^{W167S}$) (Appendix Figure S3) and assessed their melting temperature (T_m) (Fig 4A). Interestingly, whereas $OTULIN^{WT}$ and $OTULIN^{M86I}$ had a similar T_m ($OTULIN^{WT}$: 53.7°C, $OTULIN^{M86I}$: 54°C), $OTULIN^{W167S}$ was significantly more unstable with a T_m of 47.4°C (Fig 4A).

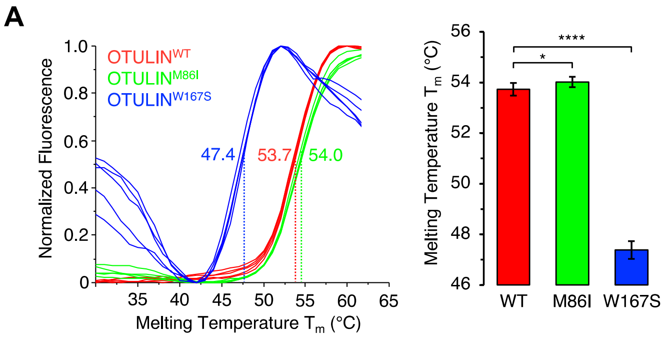

Figure 4A.
(A) DSF measurements with $OTULIN^{WT}$, $OTULIN^{M86I}$ or $OTULIN^{W167S}$. Melting temperatures (T_m) are calculated from five independent experiments with standard deviations.
*, P = 0.011; ****, P = 6.64×10^{-18}, unpaired t-test.

Apart from this, my comments are merely stylistic.

Point raised by reviewer:
Page 11, ll165: I don't think that autoUb is LUBAC accounts for all M1 chains that are present in the patient cells. This should be rephrased and a updated with a more appropriate reference.

Response:
The respective paragraph has been rephrased (see point 9 pf reviewer #1).

Point raised by reviewer:
Page 14: expression of linear ubiquitin in cells: change 'expression' to 'amount'

Response:
The respective sentence was updated accordingly.

Point raised by reviewer:
Page 16, ll267: Gyrd-Hansens lab in Oxford has performed many similar studies and should be cited alongside Draber et al.

Response and change in manuscript:
The studies by Elliott *et al.* and Hrdinka *et al.* have now been included (p. 16, l. 322): "OTULIN itself was not recruited to the TNFR1-SC (Fig 6B) in line with published data (Draber et al., 2015, Elliott, Leske et al., 2016, Hrdinka et al., 2016)."

Point raised by reviewer:
Page 19, ll328: The authors here describe three very different scenarios, and the discussion at this point, should dive into a few additional, important points.

Firstly, OTULIN loss is fundamentally different from (non-destabilising) OTULIN mutations. LUBAC, via HOIP, not only binds OTULIN, but also interacts with the CYLD/SPATA2 complex (4 papers in 2016, should be cited/included). If OTULIN is absent, the binding site on LUBAC is free to bind SPATA2/CYLD, which may have a functional consequence for LUBAC complexes (eg in terms of localisation, and recruitment to the TNFR-SC).

Second, the C129A mutant of OTULIN converted OTULIN to the highest affinity M1 chains binder present in cells, and since the protein was not destabilised, it took LUBAC out of circulation by reinforcing its autoubiquitination. This seems a special, non-physiological state that does not represent the previous or here described forms of ORAS.

In the scenario present in the here described patient, a non-destabilising OTULIN variant with lower catalytic efficiency, will still be able to bind to LUBAC and compete with CYLD/SPATA, but is unlikely to grossly affect LUBAC autoubiquitination and autoinhibition.

Response:
We thank the reviewer for his/her helpful suggestions!

We agree that we do not observe a loss of OTULIN in the patient, however, the expression of OTULIN is diminished in patient-derived cells (e.g. Fig 2A). Thus, we think that a downregulation in OTULIN might also result in increased complex formation of SPATA2/CYLD with LUBAC which might explain the increased LUBAC recruitment which we observe in our cellular setting (Fig 6B).
We also agree with this reviewer that OTULIN^{C129A} is a special, non-physiological case. However, as we pulled down LUBAC components together with the linear ubiquitin linkages in the M1 TUBE assay (Fig 2B and 6A), we think that it is likely that LUBAC autoubiquitination is still increased in patient-derived cells.

We have taken this reviewers criticism on board and rephrased the discussion accordingly to hopefully meet his/her expectations!

Change in manuscript:

We have rephrased the discussion section of the manuscript accordingly which now reads as follows (p. 18, l. 377):

At the molecular level, we demonstrate that patient-derived cells express less OTULIN protein than control cells (Fig 2A) and increased amounts of linear ubiquitin linkages under basal conditions (Fig 2B), and, importantly, also upon stimulation with TNF (Fig 6A), the cytokine identified as primary driver of inflammation in mice and humans with defective OTULIN function (Damgaard et al., 2019, Damgaard et al., 2016, Heger et al., 2018). In addition, we find linear ubiquitin linkages to be more abundant in the TNFR1-SC along with increased presence of LUBAC components as compared to control (Fig 5B). Two separate pools of cytoplasmic LUBAC complexes were identified as abundant in cells (Draber et al., 2015): (i) LUBAC in complex with OTULIN, and (ii) LUBAC in complex with CYLD, a DUB known to antagonize K63- and linear ubiquitin linkages in SCs (Kovalenko, Chable-Bessia et al., 2003), and spermatogenesis-associated protein (SPATA) 2 (Elliott et al., 2016, Kupka, De Miguel et al., 2016, Schlicher, Wissler et al., 2016, Wagner, Satpathy et al., 2016). The latter complex can be recruited to SCs, including the TNFR1-SC (Elliott et al., 2016, Kupka et al., 2016, Schlicher et al., 2016, Wagner et al., 2016). The binding of SPATA2-CYLD or OTULIN to HOIP was shown to be mutually exclusive (Draber et al., 2015, Schlicher et al., 2016). Thus, a downregulation in OTULIN expression levels, potentially due to reduced thermal stability of OTULIN^{W167S} (Fig 4A), as observed in this patient, might result in increased LUBAC-SPATA2-CYLD complex formation, and, in turn, increased recruitment of LUBAC to the TNFR1-SC (Fig 6B). This is an intriguing finding as linear ubiquitin linkages in the TNFR1-SC were unaffected by a complete absence of OTULIN (Draber et al., 2015), and expression of the catalytically inactive OTULIN^{C129A} resulted in even reduced presence of LUBAC and linear ubiquitin linkages in the TNFR1-SC (Heger et al., 2018). OTULIN deficiency or expression of catalytically inactive OTULIN^{C129A} cause increased LUBAC autoubiquitination and degradation (Damgaard et al., 2019, Damgaard et al., 2016, Heger et al., 2018, Verboom et al., 2020). In patient-derived cells, LUBAC components were pulled down together with increased amounts of linear ubiquitin linkages in patient-derived cells (Fig 2B and 6A) suggestive of enhanced LUBAC autoubiquitination in the patient. However, expression levels of LUBAC components were not altered (Fig 2A) and, in addition, we observed augmented LUBAC recruitment to the TNFR1-SC (Fig 6B) along with increased ubiquitination of the TNFR1-SC component RIPK1 (Fig 6B). This is in line with previous reports demonstrating that OTULIN can regulate ubiquitination of substrates other than LUBAC, *e.g.* RIPK1 and TNFR1 (Fiil et al., 2013, Keusekotten et al., 2013).

Thus, even subtle changes in the expression of OTULIN, LUBAC, and probably also CYLD and SPATA2, and their catalytic activities may affect the composition of the TNFR1-SC and its signaling output.

Point raised by reviewer:

Fig. 3: The quality of the structure figures, esp for depiction of the M86I variant, should be improved. In C, there are sections that seem to float in free space; in B there seems to be unnecessary detail.

The figure has been revised in accordance with the reviewer's comment.

Referee #3 (Remarks for Author):

OTULIN is a deubiquitinating enzyme (DUB) that specifically cleaves linear ubiquitin linkage. Patients having mutations in OTULIN genes exhibited autoinflammatory disease called OTULIN-related autoinflammatory syndrome (ORAS). Although previously described ORAS patients have homozygous mutations of OTULIN genes, the authors reported an ORAS patient with compound heterozygous variants (W167S/M86I). The authors showed that cells derived from patient exhibited

increased linear ubiquitylation of components of LUBAC, an enzyme complex specifically generate linear ubiquitin chains, and augmented cell death triggered by TNFalpha and cycloheximide, which are the characteristics of cells from ORAS patients. Although symptoms and clinical manifestations are different from those of ORAS patients reported previously, the reported patient can be categorized in ORAS clinically. However, biochemical analyses of the OTULIN mutants were not adequate enough to support the conclusion. Thus, the reviewer feels that this manuscript cannot be include high profile journal such as EMBO molecular medicine in the present form. The comments are listed below.

Point raised by reviewer:
#1. Since substantial amount of linear ubiquitin chains can be detected in EBV-transformed B cells from patients, both M86I and W167S mutations may affect catalytic activity of OTULIN (Figure 4D). However, OTULIN M86I could cleave linear ubiquitin chains at the comparable level to OTULIN WT when expressed in A549cells (Figure 4A). Thus, it is worth examining the Kcat value of the mutants to show the effect of the mutations on OTULIN DUB activity.

Response and change in manuscript:
To further evaluate to which extent OTULIN's catalytic activity was compromised by the two variants, we performed a DUB assay using recombinant OTULIN^{WT}, OTULIN^{M86I} and OTULIN^{W167S} on endogenous linear ubiquitin linkages isolated from A549 *OTULIN* KO cells (Fig 4C). Whereas OTULIN^{WT} was able to cleave all linear ubiquitin chains, residual amounts of linkages were still present in samples incubated with OTULIN^{W167S}. Moreover, also in samples treated with OTULIN^{M86I} we were able to detect remains of linear ubiquitin linkages, although to a lesser extent than in OTULIN^{W167S}-treated samples.

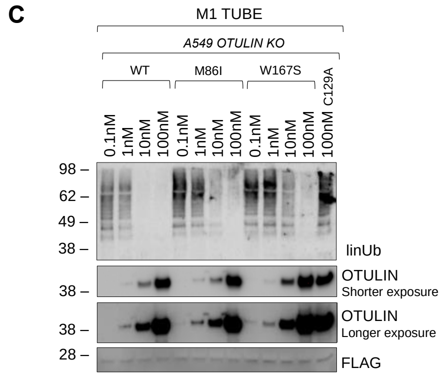

Figure 4C. (C) Linear ubiquitin linkages isolated from A549 OTULIN KO cells by M1 TUBE assay were incubated with increasing concentrations of recombinant OTULIN^{WT}, OTULIN^{M86I} and OTULIN^{W167S} or catalytically inactive OTULIN^{C129A} as control for 1h. Afterwards, samples were subjected to analysis by western blot for the indicated proteins. Images are representative of three independent experiments.

Point raised by reviewer:
#2. Although the authors claimed that reduction of the amount of OTULIN in patients may not be caused by augmented degradation by the proteasome (Supplementary Fig 1D), it was not convincing. The reviewer recommends the authors to examine the half-life of the OTULIN mutants by treating cells with cycloheximide in the presence or absence of proteasome inhibitors (cycloheximide chase analysis).

Response:
We determined the half-life of OTULIN^{WT}, OTULIN^{M86I} and OTULIN^{W167S} as suggested by this reviewer (Fig EV2D). Next, we determined whether OTULIN protein expression was stabilized in the presence of either Bortezomib (BTZ) or Bafilomycin A1 (Fig EV2E; for results see also point #8 of reviewer #1). Results indicate that OTULIN protein is neither stabilized by the addition of BTZ nor Bafilomycin A1.

Point raised by reviewer:

#3. It is worth examining whether the mutants can bind to linear ubiquitin or not.

Response:
This is a valid point! Please see our answer to point #10 of reviewer #1.

Point raised by reviewer:
#4. It is interesting to know the amount of OTULIN in EBV-transformed B cells from parents.

Response and change in manuscript:
This information was provided in Figure 5D (M1 TUBE assay in B cells from healthy control, patient, mother and father). As this information was apparently not visible enough to the reader, we now included a separate analysis of OTULIN protein expression in healthy control, patient, father and mother. In addition, we also included data on the mRNA expression of OTULIN in maternal and paternal B cells. Whereas we did not observe any changes in OTULIN mRNA expression in B cells from healthy control, patient, mother and father, we observed a downregulation of OTULIN protein expression not only in B cells from the patient but also in B cells from mother and father as quantified in a densitometric analysis (n=5 independent experiments).

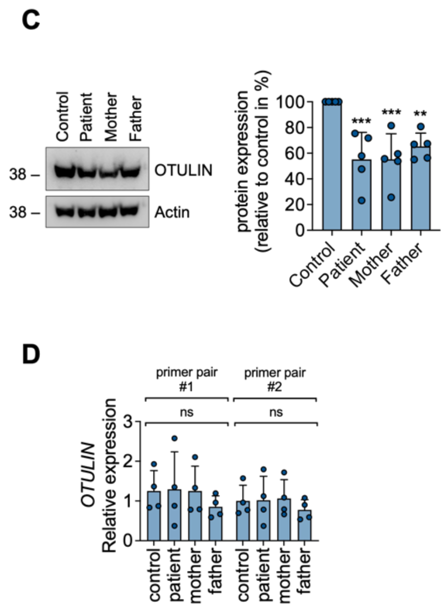

Figure EV3C and D. (C) OTULIN protein expression in B cells derived from control, patient, mother, and father is shown. Blots (left panel) are representative of five independent experiments analyzed by densitometry (right panel). Data are presented as mean ± SD (dots represent individual experiments); ***, P = 0.0008, **, P < 0.0069, ordinary one-way ANOVA, Dunnett's multiple comparisons test. (D) Relative mRNA expression of OTULIN in B cells derived from control, patient, mother, and father determined with two different primer pairs is depicted. Data are presented as mean ± SD of four independent experiments; dots represent individual experiments performed in three technical replicates; ns, non-significant, ordinary one-way ANOVA, Dunnett's multiple comparisons test.

Point raised by reviewer:
#5. The reviewer is curious to know whether linear ubiquitination of HOIL-1 also increased in patient cells.

Response:
As can be seen in Fig 6A (M1-TUBE assay in control and patient-derived fibroblasts upon stimulation with TNF) increased amounts of HOIL-1 are pulled down with linear ubiquitin linkages in patient-derived fibroblasts as compared to control suggesting increased linear ubiquitination of HOIL-1. Detection of HOIL-1 in immunoprecipitation or pull-down experiments is complicated by the fact that the heavy chain of the antibodies used for immunoprecipitation or pull-down is running at approximately the same size of HOIL-1. This is why detection of HOIL-1 was only successful in some of the experiments.

References:

Chesi A, Grant SFA (2015) The Genetics of Pediatric Obesity. *Trends Endocrinol Metab* 26: 711-721

Damgaard RB, Elliott PR, Swatek KN, Maher ER, Stepensky P, Elpeleg O, Komander D, Berkun Y (2019) OTULIN deficiency in ORAS causes cell type-specific LUBAC degradation, dysregulated TNF signalling and cell death. *EMBO Mol Med* 11

Damgaard RB, Jolin HE, Allison MED, Davies SE, Titheradge HL, McKenzie ANJ, Komander D (2020) OTULIN protects the liver against cell death, inflammation, fibrosis, and cancer. *Cell Death Differ* 27: 1457-1474

Damgaard RB, Walker JA, Marco-Casanova P, Morgan NV, Titheradge HL, Elliott PR, McHale D, Maher ER, McKenzie ANJ, Komander D (2016) The Deubiquitinase OTULIN Is an Essential Negative Regulator of Inflammation and Autoimmunity. *Cell* 166: 1215-1230 e20

Draber P, Kupka S, Reichert M, Draberova H, Lafont E, de Miguel D, Spilgies L, Surinova S, Taraborrelli L, Hartwig T, Rieser E, Martino L, Rittinger K, Walczak H (2015) LUBAC-Recruited CYLD and A20 Regulate Gene Activation and Cell Death by Exerting Opposing Effects on Linear Ubiquitin in Signaling Complexes. *Cell Rep* 13: 2258-72

Elliott PR, Leske D, Hrdinka M, Bagola K, Fiil BK, McLaughlin SH, Wagstaff J, Volkmar N, Christianson JC, Kessler BM, Freund SM, Komander D, Gyrd-Hansen M (2016) SPATA2 Links CYLD to LUBAC, Activates CYLD, and Controls LUBAC Signaling. *Mol Cell* 63: 990-1005

Elliott PR, Nielsen SV, Marco-Casanova P, Fiil BK, Keusekotten K, Mailand N, Freund SM, Gyrd-Hansen M, Komander D (2014) Molecular basis and regulation of OTULIN-LUBAC interaction. *Mol Cell* 54: 335-48

Fiil BK, Damgaard RB, Wagner SA, Keusekotten K, Fritsch M, Bekker-Jensen S, Mailand N, Choudhary C, Komander D, Gyrd-Hansen M (2013) OTULIN restricts Met1-linked ubiquitination to control innate immune signaling. *Mol Cell* 50: 818-830

Guzzaloni G, Grugni G, Minocci A, Moro D, Morabito F (2000) Liver steatosis in juvenile obesity: correlations with lipid profile, hepatic biochemical parameters and glycemic and insulinemic responses to an oral glucose tolerance test. *Int J Obes Relat Metab Disord* 24: 772-6

Haas L, Chevalier R, Major BT, Enders F, Kumar S, Tung J (2017) Biologic Agents Are Associated with Excessive Weight Gain in Children with Inflammatory Bowel Disease. *Dig Dis Sci* 62: 3110-3116

Heger K, Wickliffe KE, Ndoja A, Zhang J, Murthy A, Dugger DL, Maltzman A, de Sousa EMF, Hung J, Zeng Y, Verschueren E, Kirkpatrick DS, Vucic D, Lee WP, Roose-Girma M, Newman RJ, Warming S, Hsiao YC, Komuves LG, Webster JD et al. (2018) OTULIN limits cell death and inflammation by deubiquitinating LUBAC. *Nature* 559: 120-124

Hrdinka M, Fiil BK, Zucca M, Leske D, Bagola K, Yabal M, Elliott PR, Damgaard RB, Komander D, Jost PJ, Gyrd-Hansen M (2016) CYLD Limits Lys63- and Met1-Linked Ubiquitin at Receptor Complexes to Regulate Innate Immune Signaling. *Cell Rep* 14: 2846-58

Jahan AS, Elbaek CR, Damgaard RB (2021) Met1-linked ubiquitin signalling in health and disease: inflammation, immunity, cancer, and beyond. *Cell Death Differ* 28: 473-492

Keusekotten K, Elliott PR, Glockner L, Fiil BK, Damgaard RB, Kulathu Y, Wauer T, Hospenthal MK, Gyrd-Hansen M, Krappmann D, Hofmann K, Komander D (2013) OTULIN antagonizes LUBAC signaling by specifically hydrolyzing Met1-linked polyubiquitin. *Cell* 153: 1312-26

Kovalenko A, Chable-Bessia C, Cantarella G, Israel A, Wallach D, Courtois G (2003) The tumour suppressor CYLD negatively regulates NF-kappaB signalling by deubiquitination. *Nature* 424: 801-5
Kupka S, De Miguel D, Draber P, Martino L, Surinova S, Rittinger K, Walczak H (2016) SPATA2-Mediated Binding of CYLD to HOIP Enables CYLD Recruitment to Signaling Complexes. *Cell Rep* 16: 2271-80
Rivkin E, Almeida SM, Ceccarelli DF, Juang YC, MacLean TA, Srikumar T, Huang H, Dunham WH, Fukumura R, Xie G, Gondo Y, Raught B, Gingras AC, Sicheri F, Cordes SP (2013) The linear ubiquitin-specific deubiquitinase gumby regulates angiogenesis. *Nature* 498: 318-24
Rouillard AD, Gundersen GW, Fernandez NF, Wang Z, Monteiro CD, McDermott MG, Ma'ayan A (2016) The harmonizome: a collection of processed datasets gathered to serve and mine knowledge about genes and proteins. *Database (Oxford)* 2016
Schlicher L, Wissler M, Preiss F, Brauns-Schubert P, Jakob C, Dumit V, Borner C, Dengjel J, Maurer U (2016) SPATA2 promotes CYLD activity and regulates TNF-induced NF-kappaB signaling and cell death. *EMBO Rep* 17: 1485-1497
Shimizu Y, Peltzer N, Sevko A, Lafont E, Sarr A, Draberova H, Walczak H (2017) The Linear ubiquitin chain assembly complex acts as a liver tumor suppressor and inhibits hepatocyte apoptosis and hepatitis. *Hepatology* 65: 1963-1978
van Wijk SJL, Fricke F, Herhaus L, Gupta J, Hotte K, Pampaloni F, Grumati P, Kaulich M, Sou YS, Komatsu M, Greten FR, Fulda S, Heilemann M, Dikic I (2017) Linear ubiquitination of cytosolic Salmonella Typhimurium activates NF-kappaB and restricts bacterial proliferation. *Nat Microbiol* 2: 17066
Verboom L, Hoste E, van Loo G (2021) OTULIN in NF-kappaB signaling, cell death, and disease. *Trends Immunol* 42: 590-603
Verboom L, Martens A, Priem D, Hoste E, Sze M, Vikkula H, Van Hove L, Voet S, Roels J, Maelfait J, Bongiovanni L, de Bruin A, Scott CL, Saeys Y, Pasparakis M, Bertrand MJM, van Loo G (2020) OTULIN Prevents Liver Inflammation and Hepatocellular Carcinoma by Inhibiting FADD- and RIPK1 Kinase-Mediated Hepatocyte Apoptosis. *Cell Rep* 30: 2237-2247 e6
Wagner SA, Satpathy S, Beli P, Choudhary C (2016) SPATA2 links CYLD to the TNF-alpha receptor signaling complex and modulates the receptor signaling outcomes. *EMBO J* 35: 1868-84
Wang K, Kim C, Bradfield J, Guo Y, Toskala E, Otieno FG, Hou C, Thomas K, Cardinale C, Lyon GJ, Golhar R, Hakonarson H (2013) Whole-genome DNA/RNA sequencing identifies truncating mutations in RBCK1 in a novel Mendelian disease with neuromuscular and cardiac involvement. *Genome Med* 5: 67
Wang K, Li WD, Zhang CK, Wang Z, Glessner JT, Grant SF, Zhao H, Hakonarson H, Price RA (2011) A genome-wide association study on obesity and obesity-related traits. *PLoS One* 6: e18939
Warner ET, Jiang L, Adjei DN, Turman C, Gordon W, Wang L, Tamimi R, Kraft P, Lindstrom S (2021) A Genome-Wide Association Study of Childhood Body Fatness. *Obesity (Silver Spring)* 29: 446-453
Weinelt N, van Wijk SJL (2021) Ubiquitin-dependent and -independent functions of OTULIN in cell fate control and beyond. *Cell Death Differ* 28: 493-504
Zhou Q, Yu X, Demirkaya E, Deuitch N, Stone D, Tsai WL, Kuehn HS, Wang H, Yang D, Park YH, Ombrello AK, Blake M, Romeo T, Remmers EF, Chae JJ, Mullikin JC, Guzel F, Milner JD, Boehm M, Rosenzweig SD et al. (2016) Biallelic hypomorphic mutations in a linear

deubiquitinase define otulipenia, an early-onset autoinflammatory disease. *Proc Natl Acad Sci U S A* 113: 10127-32

1st Revision - Editorial Decision 17th Jan 2022

17th Jan 2022

Dear Dr. Fischer-Posovszky,

Thank you for the submission of your revised manuscript to EMBO Molecular Medicine. I am pleased to inform you that we will be able to accept your manuscript pending the following final amendments:

1) Please address the point raised by the referee #1.
2) Figures: Please submit all EV Figures as separate, high-resolution files.
3) In the main manuscript file, please do the following:
- Make sure that all special characters display well.
- Add callouts for Figure 6H, EV Figure 5H and Appendix Tables S5-7.
- Remove Table EV1 and uploaded it as a separate file.
- Remove data not shown (p.39).
- In M&M, provide the antibody dilutions that were used for each antibody.
- In M&M, a statistical paragraph that should reflect all information that you have filled in the Authors Checklist, especially regarding randomization, blinding, replication.
- Correct the reference citation. In the reference list, citations should be listed in alphabetical order. Where there are more than 10 authors on a paper, 10 will be listed, followed by "et al.". Please check "Author Guidelines" for more information.
https://www.embopress.org/page/journal/17574684/authorguide#referencesformat
- Raw data from large-scale datasets (WES) should be deposited in one of the relevant databases and made freely available prior the publication of the manuscript. Use the following format to report the accession number of your data:

The datasets produced in this study are available in the following databases:
[data type]: [full name of the resource] [accession number/identifier] ([doi or URL or identifiers.org/DATABASE:ACCESSION])

Please check "Author Guidelines" for more information.
https://www.embopress.org/page/journal/17574684/authorguide#availabilityofpublishedmaterial
4) Appendix: Move "Appendix Supplementary Methods" to the "Materials and Methods" section in the main manuscript file.
5) Conflict of interest: We updated our journal's competing interests policy in January 2022 and request authors to consider both actual and perceived competing interests. Please review the policy https://www.embopress.org/competing-interests and update your competing interests if necessary
6) Source data: Please upload one file per figure (zipp were appropriate) for the main Figures as .pdf or .xlsx (.pptx is not appropriate format), and, if provided, zipp source data for all EV Figures as one file.
7) Synopsis: Please check your synopsis text and image, revise them if necessary and submit their final versions with your revised manuscript. Please be aware that in the proof stage minor corrections only are allowed (e.g., typos).
8) As part of the EMBO Publications transparent editorial process initiative (see our Editorial at http://embomolmed.embopress.org/content/2/9/329), EMBO Molecular Medicine will publish online a Review Process File (RPF) to accompany accepted manuscripts. This file will be published in conjunction with your paper and will include the anonymous referee reports, your point-by-point response and all pertinent correspondence relating to the manuscript. Let us know whether you agree with the publication of the RPF and as here, if you want to remove or not any figures from it prior to publication. Please note that the Authors checklist will be published at the end of the RPF.
9) Please provide a point-by-point letter INCLUDING my comments as well as the reviewer's reports and your detailed responses (as Word file).

I look forward to reading a new revised version of your manuscript as soon as possible.

Yours sincerely,

Zeljko Durdevic

Zeljko Durdevic
Editor
EMBO Molecular Medicine

*

***** Reviewer's comments *****

Referee #1 (Comments on Novelty/Model System for Author):

Experiments performed in relevant patient cells and cell lines

Referee #1 (Remarks for Author):

The authors have done a very good job revising this manuscript, for which they should be commended. They have included a variety of new data to support their conclusions and clarify points raised by the referees. Overall, this satisfactorily addresses the points and questions I raised.

I only have one point that I would like the authors to consider:

In my original point 12), I asked the authors to reconsider their discussion and focus on LUBAC autoubiquitination as the main functional deficiency and explanation of their observed phenotype. In the rebuttal, the authors reply that "As shown in the M1 TUBE experiments in Figures 2B, 4D and 5A, LUBAC components, but not OTULIN, are pulled down together with linear ubiquitin linkages. These results are in line with data published by Heger et al., Nature, 2018 (see Figure 2a), in which they show that catalytically inactive OTULINC129A results in increased LUBAC autoubiquitination". First, I agree with the authors that LUBAC components are increased in their M1-pulldowns in Figs 2B, 4D and 5A. However, I will argue that this is not evidence of these components being ubiquitinated. The molecular weight of these components is not increased. The molecular weights are identical to those in the input blots and therefore cannot be ubiquitinated. Rather, the pulldowns in Figs 2, 4 and 5 show that LUBAC is associated with, recruited to, or in complex with more M1-ubiquitin in patient cells compared with controls, but not increased in ubiquitnation, however inexplicable or unlikely this may seem. The authors argue that their data are consistent with those of Heger et al, Nature 2018. I would argue they are not. Heger and colleagues show that HOIP is modified by M1-Ub, as is SHARPIN, in their M1-Ub pulldowns (Fig 1a). This is evident as higher molecular weight signals in the blots for these components, as is also observed by Damgaard, Hrdinka, Verboom, and others in the absence of OTULIN. It is not impossible that LUBAC components are more ubiquitinated in patient cells in this study. I just do not think the authors provide any actual evidence of this, and their model of LUBAC autoubiquitination being the driver of pathology is therefore speculative. The authors have performed key additional experiments to assess basal signalling and NF-kB activation, which are also not increased (rather decreased) in the patient. So it is not clear what mechanisms exactly drives disease. Perhaps it is a combination of slightly increased susceptibility to cell death, dysregulated signalling and increased RIPK1 ubiquitination, or some aspect that is not investigated. Regardless, I think the authors should acknowledge that there is no evidence for their LUBAC ubiquitnation model and indicate in the text that this is speculative and inferred from other studies. I acknowledge this is partly achieved in their revised text. But I think the word "suggestive" in the sentence "In patient-derived cells, LUBAC components were pulled down together with increased amounts of linear ubiquitin linkages in patient-derived cells (Fig 2B and 6A) suggestive of enhanced LUBAC autoubiquitination in the patient" indicates that there is some data supporting this. And I really do not think such data has been presented.

Besides this, I have no further comments or questions. I think it is a very good manuscript that deserves publication.

Referee #3 (Remarks for Author):

The reviewer was surprised that binding of both OTULIN mutants to M1-ubiquitin is only marginally affected. Also, they did not evaluate Kcat value of the OUTLIN mutants. The reviewer feels that biochemical analyses provided in this manuscript may not explain the phenotypical symptoms of the patient. However, the analyses themselves are well-performed and informative. The Authors satisfactorily addressed the concerns raised by the reviewer, and that this manuscript is worth including EMBO Molecular Medicine.

Response to editorial requests

1) Please address the point raised by the referee #1.
We have addressed the point of referee #1 (see below) as requested.

2) Figures: Please submit all EV Figures as separate, high-resolution files.
We have prepared high-resolution files of all EV figures.

3) In the main manuscript file, please do the following:

- Make sure that all special characters display well.
We carefully checked the manuscript to make sure that this is the case.

- Add callouts for Figure 6H, EV Figure 5H and Appendix Tables S5-7.
We apologize for not having included these callouts and included them accordingly for Figures 6H and EV**4**H. The callout for Appendix Tables S4 – S7 was already included in the first revision (p. 9, l. 155).

- Remove Table EV1 and uploaded it as a separate file.
This has been done.

- Remove data not shown (p.39).
We removed "Not shown: distal ubiquitin." from the legend of Figure 3.

- In M&M, provide the antibody dilutions that were used for each antibody.
The respective information was added.

- In M&M, a statistical paragraph that should reflect all information that you have filled in the Authors Checklist, especially regarding randomization, blinding, replication.
A paragraph reflecting all information of the Authors Checklist has been included in the Material & Methods.

- Correct the reference citation. In the reference list, citations should be listed in alphabetical order. Where there are more than 10 authors on a paper, 10 will be listed, followed by "et al.". Please check "Author Guidelines" for more information.
https://www.embopress.org/page/journal/17574684/authorguide#referencesformat
The reference list has been updated.

- Raw data from large-scale datasets (WES) should be deposited in one of the relevant databases and made freely available prior the publication of the manuscript. Use the following format to report the accession number of your data:

The datasets produced in this study are available in the following databases: [data type]: [full name of the resource] [accession number/identifier] ([doi or URL or identifiers.org/DATABASE:ACCESSION])

Please check "Author Guidelines" for more information.
https://www.embopress.org/page/journal/17574684/authorguide#availabilityofpublishedmaterial
Explicit consent for disclosure of entire WES data sets in a public data base had not been obtained. Therefore, there are no such data deposited in external repositories. A corresponding statement has been included in the manuscript.

4) Appendix: Move "Appendix Supplementary Methods" to the "Materials and Methods" section in the main manuscript file.
This has been done.

5) Conflict of interest: We updated our journal's competing interests policy in January 2022 and request authors to consider both actual and perceived competing interests. Please review the policy https://www.embopress.org/competing-interests and update your competing interests if necessary
We reviewed the policy and still declare that the authors have no relevant conflicts of interest.

6) Source data: Please upload one file per figure (zipp were appropriate) for the main Figures as .pdf or .xlsx (.pptx is not appropriate format), and, if provided, zipp source data for all EV Figures as one file.
This has been done.

7) Synopsis: Please check your synopsis text and image, revise them if necessary and submit their final versions with your revised manuscript. Please be aware that in the proof stage minor corrections only are allowed (e.g., typos).

Synopsis

The OTULIN-Related Autoinflammatory Syndrome (ORAS) is known to be caused by homozygous variants in the *OTULIN* gene. This study discovers that two new compound-heterozygous variants in *OTULIN* are associated with an atypical, but potentially fatal, late-onset form of ORAS.

- Atypical ORAS is characterized by multiorgan abscess formation and bears the risk of being clinically inapparent.
- OTULIN variants p.M86I and p.W167S disrupt OTULIN function leading to increased levels of linear ubiquitin linkages in patient-derived fibroblasts and B cells.
- Molecularly, atypical ORAS resembles classical ORAS with enhanced TNF production in monocytes, but diminished TNF-induced gene activation and sensitization to TNF-mediated cell death (in the presence of cycloheximide) in fibroblasts.
- Anti-TNF therapy with Adalimumab resulted in normalization of the patient's inflammatory plasma protein profile suggesting that patients with atypical ORAS might benefit from TNF-blocking agents as seen in patients with classical ORAS.

8) As part of the EMBO Publications transparent editorial process initiative (see our Editorial at http://embomolmed.embopress.org/content/2/9/329), EMBO Molecular Medicine will publish online a Review Process File (RPF) to accompany accepted manuscripts. This file will be published in conjunction with your paper and will include the anonymous referee reports, your point-by-point response and all pertinent correspondence relating to the manuscript. Let us know whether you agree with the publication of the RPF and as here, if you want to remove or not any figures from it prior to publication. Please note that the Authors checklist will be published at the end of the RPF.
We agree with the publication of the RPF and do not wish to remove any figures from it prior to publication.

9) Please provide a point-by-point letter INCLUDING my comments as well as the reviewer's reports and your detailed responses (as Word file).
This has been done.

Response to reviewers' requests

Referee #1 (Comments on Novelty/Model System for Author):

Experiments performed in relevant patient cells and cell lines

Referee #1 (Remarks for Author):

The authors have done a very good job revising this manuscript, for which they should be commended. They have included a variety of new data to support their conclusions and clarify points raised by the referees. Overall, this satisfactorily addresses the points and questions I raised.

We thank this reviewer again for his constructive criticism and positive evaluation of our work!

I only have one point that I would like the authors to consider:

Point raised by reviewer:
In my original point 12), I asked the authors to reconsider their discussion and focus on LUBAC autoubiquitination as the main functional deficiency and explanation of their observed phenotype. In the rebuttal, the authors reply that "As shown in the M1 TUBE experiments in Figures 2B, 4D and 5A, LUBAC components, but not OTULIN, are pulled down together with linear ubiquitin linkages. These results are in line with data published by Heger et al., Nature, 2018 (see Figure 2a), in which they show that catalytically inactive OTULINC129A results in increased LUBAC autoubiquitination". First, I agree with the authors that LUBAC components are increased in their M1-pulldowns in Figs 2B, 4D and 5A. However, I will argue that this is not evidence of these components being ubiquitinated. The molecular weight of these components is not increased. The molecular weights are identical to those in the input blots and therefore cannot be ubiquitinated. Rather, the pulldowns in Figs 2, 4 and 5 show that LUBAC is associated with, recruited to, or in complex with more M1-ubiquitin in patient cells compared with controls, but not increased in ubiquitnation, however inexplicable or unlikely this may seem. The authors argue that their data are consistent with those of Heger et al, Nature 2018. I would argue they are not. Heger and colleagues show that HOIP is modified by M1-Ub, as is SHARPIN, in their M1-Ub pulldowns (Fig 1a). This is evident as higher molecular weight signals in the blots for these components, as is also observed by Damgaard, Hrdinka, Verboom, and others in the absence of OTULIN. It is not impossible that LUBAC components are more ubiquitinated in patient cells in this study. I just do not think the authors provide any actual evidence of this, and their model of LUBAC autoubiquitination being the driver of pathology is therefore speculative. The authors have performed key additional experiments to assess basal signalling and NF-kB activation, which are also not increased (rather decreased) in the patient. So it is not clear what mechanisms exactly drives disease. Perhaps it is a combination of slightly increased susceptibility to cell death, dysregulated signalling and increased RIPK1 ubiquitination, or some aspect that is not investigated. Regardless, I think the authors should acknowledge that there is no evidence for their LUBAC ubiquitnation model and indicate in the text that this is speculative and inferred from other studies. I acknowledge this is partly achieved in their revised text. But I think the word "suggestive" in the sentence "In patient-derived cells, LUBAC components were pulled down together with increased amounts of linear ubiquitin linkages in patient-derived cells (Fig 2B and 6A) suggestive of enhanced LUBAC autoubiquitination in the patient" indicates that there is some data supporting this. And I really do not think such data has been presented.

Answer and change in manuscript:
We thank this reviewer for his careful reading of our manuscript and his critique.

We think that the lack of laddering on top of the LUBAC components in our samples might be due to the mild phenotype of the patient with only a slight increase in linear ubiquitin linkages as compared to a cellular setting with OTULIN^{C129A} as in Heger *et al.*, Nature, 2018. Also Heger and colleagues show in their M1 TUBE assay (Figure 2a) that the main proportion of the protein pulled down with the M1 TUBE is unmodified. Thus, we think it might well be that the proportion of linearly modified LUBAC components in our assay is simply below detection limit.
In any case, we agree with this reviewer that we have not provided ultimate proof of linear ubiquitination of LUBAC components in patient-derived cells. By using the phrase "suggestive" we did not mean to imply that our data proved this nor are we of the opinion that LUBAC autoubiquitination is the driver of the pathology observed in the patient. Thus, we have removed the respective part of the sentence. Instead, we have added the following sentence: "Whether LUBAC components are modified by linear ubiquitin linkages in patient-derived cells, however, requires further investigation."

Besides this, I have no further comments or questions. I think it is a very good manuscript that deserves publication.

Referee #3 (Remarks for Author):

The reviewer was surprised that binding of both OTULIN mutants to M1-ubiquitin is only marginally affected. Also, they did not evaluate Kcat value of the OUTLIN mutants. The reviewer feels that biochemical analyses provided in this manuscript may not explain the phenotypical symptoms of the patient. However, the analyses themselves are well-performed and informative. The Authors satisfactorily addressed the concerns raised by the reviewer, and that this manuscript is worth including EMBO Molecular Medicine.

We thank this reviewer for his feedback on our work!

We are pleased to inform you that your manuscript is accepted for publication and is now being sent to our publisher to be included in the next available issue of EMBO Molecular Medicine.

EMBO PRESS

YOU MUST COMPLETE ALL CELLS WITH A PINK BACKGROUND ↓

PLEASE NOTE THAT THIS CHECKLIST WILL BE PUBLISHED ALONGSIDE YOUR PAPER

Corresponding Author Name: Pamela Fischer-Posovszky
Journal Submitted to: EMBO Molecular Medicine
Manuscript Number: EMM-2021-14901

USEFUL LINKS FOR COMPLETING THIS FORM

http://www.antibodypedia.com
http://1degreebio.org
http://www.equator-network.org/reporting-guidelines/improving-bioscience-research-repor
http://grants.nih.gov/grants/olaw/olaw.htm
http://www.mrc.ac.uk/Ourresearch/Ethicsresearchguidance/Useofanimals/index.htm
http://ClinicalTrials.gov
http://www.consort-statement.org
http://www.consort-statement.org/checklists/view/32-consort/66-title
http://www.equator-network.org/reporting-guidelines/reporting-recommendations-for-tumo
http://datadryad.org
http://figshare.com
http://www.ncbi.nlm.nih.gov/gap
http://www.ebi.ac.uk/ega
http://biomodels.net/
http://biomodels.net/miriam/
http://jjj.biochem.sun.ac.za
https://osp.od.nih.gov/biosafety-biosecurity-and-emerging-biotechnology/
http://www.selectagents.gov/

Reporting Checklist For Life Sciences Articles (Rev. June 2017)

This checklist is used to ensure good reporting standards and to improve the reproducibility of published results. These guidelines are consistent with the Principles and Guidelines for Reporting Preclinical Research issued by the NIH in 2014. Please follow the journal's authorship guidelines in preparing your manuscript.

A- Figures

1. Data

The data shown in figures should satisfy the following conditions:

- the data were obtained and processed according to the field's best practice and are presented to reflect the results of the experiments in an accurate and unbiased manner.
- figure panels include only data points, measurements or observations that can be compared to each other in a scientifically meaningful way.
- graphs include clearly labeled error bars for independent experiments and sample sizes. Unless justified, error bars should not be shown for technical replicates.
- if n< 5, the individual data points from each experiment should be plotted and any statistical test employed should be justified
- Source Data should be included to report the data underlying graphs. Please follow the guidelines set out in the author ship guidelines on Data Presentation.

2. Captions

Each figure caption should contain the following information, for each panel where they are relevant:

- a specification of the experimental system investigated (eg cell line, species name).
- the assay(s) and method(s) used to carry out the reported observations and measurements
- an explicit mention of the biological and chemical entity(ies) that are being measured.
- an explicit mention of the biological and chemical entity(ies) that are altered/varied/perturbed in a controlled manner.

- the exact sample size (n) for each experimental group/condition, given as a number, not a range;
- a description of the sample collection allowing the reader to understand whether the samples represent technical or biological replicates (including how many animals, litters, cultures, etc.).
- a statement of how many times the experiment shown was independently replicated in the laboratory.
- definitions of statistical methods and measures:
  - common tests, such as t-test (please specify whether paired vs. unpaired), simple χ^2 tests, Wilcoxon and Mann-Whitney tests, can be unambiguously identified by name only, but more complex techniques should be described in the methods section;
  - are tests one-sided or two-sided?
  - are there adjustments for multiple comparisons?
  - exact statistical test results, e.g., P values = x but not P values < x;
  - definition of 'center values' as median or average;
  - definition of error bars as s.d. or s.e.m.

Any descriptions too long for the figure legend should be included in the methods section and/or with the source data.

In the pink boxes below, please ensure that the answers to the following questions are reported in the manuscript itself. Every question should be answered. If the question is not relevant to your research, please write NA (non applicable). We encourage you to include a specific subsection in the methods section for statistics, reagents, animal models and human subjects.

B- Statistics and general methods

Please fill out these boxes ↓ (Do not worry if you cannot see all your text once you press return)

1.a. How was the sample size chosen to ensure adequate power to detect a pre-specified effect size?	NA
1.b. For animal studies, include a statement about sample size estimate even if no statistical methods were used.	NA
2. Describe inclusion/exclusion criteria if samples or animals were excluded from the analysis. Were the criteria pre-established?	NA
3. Were any steps taken to minimize the effects of subjective bias when allocating animals/samples to treatment (e.g. randomization procedure)? If yes, please describe.	NA
For animal studies, include a statement about randomization even if no randomization was used.	NA
4.a. Were any steps taken to minimize the effects of subjective bias during group allocation or/and when assessing results (e.g. blinding of the investigator)? If yes please describe.	NA
4.b. For animal studies, include a statement about blinding even if no blinding was done	NA
5. For every figure, are statistical tests justified as appropriate?	This information can be found in the Materials and Methods in the paragraph called "Statistical analysis".
Do the data meet the assumptions of the tests (e.g., normal distribution)? Describe any methods used to assess it.	This information can be found in the Materials and Methods in the paragraph called "Statistical analysis".
Is there an estimate of variation within each group of data?	This information can be found in the Materials and Methods in the paragraph called "Statistical analysis".

Is the variance similar between the groups that are being statistically compared?	This information can be found in the Materials and Methods in the paragraph called "Statistical analysis".

C- Reagents

6. To show that antibodies were profiled for use in the system under study (assay and species), provide a citation, catalog number and/or clone number, supplementary information or reference to an antibody validation profile. e.g., Antibodypedia (see link list at top right), 1DegreeBio (see link list at top right).	This information can be found in the Materials and Methods in the paragraph called "Antibodies".
7. Identify the source of cell lines and report if they were recently authenticated (e.g., by STR profiling) and tested for mycoplasma contamination.	This information can be found in the Materials and Methods in the paragraph called "Cell lines and materials".

* for all hyperlinks, please see the table at the top right of the document

D- Animal Models

8. Report species, strain, gender, age of animals and genetic modification status where applicable. Please detail housing and husbandry conditions and the source of animals.	NA
9. For experiments involving live vertebrates, include a statement of compliance with ethical regulations and identify the committee(s) approving the experiments.	NA
10. We recommend consulting the ARRIVE guidelines (see link list at top right) (PLoS Biol. 8(6), e1000412, 2010) to ensure that other relevant aspects of animal studies are adequately reported. See author guidelines, under 'Reporting Guidelines'. See also: NIH (see link list at top right) and MRC (see link list at top right) recommendations. Please confirm compliance.	NA

E- Human Subjects

11. Identify the committee(s) approving the study protocol.	This information can be found in the Materials and Methods in the paragraph called "Cell lines and materials".
12. Include a statement confirming that informed consent was obtained from all subjects and that the experiments conformed to the principles set out in the WMA Declaration of Helsinki and the Department of Health and Human Services Belmont Report.	This information can be found in the Materials and Methods in the paragraph called "Cell lines and materials".
13. For publication of patient photos, include a statement confirming that consent to publish was obtained.	NA
14. Report any restrictions on the availability (and/or on the use) of human data or samples.	This information can be found in the Materials and Methods in the paragraph called "Cell lines and materials".
15. Report the clinical trial registration number (at ClinicalTrials.gov or equivalent), where applicable.	NA
16. For phase II and III randomized controlled trials, please refer to the CONSORT flow diagram (see link list at top right) and submit the CONSORT checklist (see link list at top right) with your submission. See author guidelines, under 'Reporting Guidelines'. Please confirm you have submitted this list.	NA
17. For tumor marker prognostic studies, we recommend that you follow the REMARK reporting guidelines (see link list at top right). See author guidelines, under 'Reporting Guidelines'. Please confirm you have followed these guidelines.	NA

F- Data Accessibility

18: Provide a "Data Availability" section at the end of the Materials & Methods, listing the accession codes for data generated in this study and deposited in a public database (e.g. RNA-Seq data: Gene Expression Omnibus GSE39462, Proteomics data: PRIDE PXD000208 etc.) Please refer to our author guidelines for 'Data Deposition'.<br><br>Data deposition in a public repository is mandatory for:<br>a. Protein, DNA and RNA sequences<br>b. Macromolecular structures<br>c. Crystallographic data for small molecules<br>d. Functional genomics data<br>e. Proteomics and molecular interactions	NA
19. Deposition is strongly recommended for any datasets that are central and integral to the study; please consider the journal's data policy. If no structured public repository exists for a given data type, we encourage the provision of datasets in the manuscript as a Supplementary Document (see author guidelines under 'Expanded View' or in unstructured repositories such as Dryad (see link list at top right) or Figshare (see link list at top right).	NA
20. Access to human clinical and genomic datasets should be provided with as few restrictions as possible while respecting ethical obligations to the patients and relevant medical and legal issues. If practically possible and compatible with the individual consent agreement used in the study, such data should be deposited in one of the major public access-controlled repositories such as dbGAP (see link list at top right) or EGA (see link list at top right).	This information can be found in the Materials and Methods in the paragraph called "Data availability".
21. Computational models that are central and integral to a study should be shared without restrictions and provided in a machine-readable form. The relevant accession numbers or links should be provided. When possible, standardized format (SBML, CellML) should be used instead of scripts (e.g. MATLAB). Authors are strongly encouraged to follow the MIRIAM guidelines (see link list at top right) and deposit their model in a public database such as Biomodels (see link list at top right) or JWS Online (see link list at top right). If computer source code is provided with the paper, it should be deposited in a public repository or included in supplementary information.	NA

G- Dual use research of concern

22. Could your study fall under dual use research restrictions? Please check biosecurity documents (see link list at top right) and list of select agents and toxins (APHIS/CDC) (see link list at top right). According to our biosecurity guidelines, provide a statement only if it could.	NA
